# Improving Aqueous Solubility and In Vitro Pharmacokinetic Properties of the 3-Nitroimidazo[1,2-*a*]pyridine Antileishmanial Pharmacophore

**DOI:** 10.3390/ph15080998

**Published:** 2022-08-13

**Authors:** Romain Paoli-Lombardo, Nicolas Primas, Sandra Bourgeade-Delmas, Sébastien Hutter, Alix Sournia-Saquet, Clotilde Boudot, Emilie Brenot, Caroline Castera-Ducros, Sophie Corvaisier, Marc Since, Aurélie Malzert-Fréon, Bertrand Courtioux, Alexis Valentin, Pierre Verhaeghe, Nadine Azas, Pascal Rathelot, Patrice Vanelle

**Affiliations:** 1 CNRS, ICR UMR 7273, Team Pharmaco-Chimie Radicalaire, Faculté de Pharmacie, Aix Marseille University, 27 Boulevard Jean Moulin, CS30064, CEDEX 05, 13385 Marseille, France; 2 Service Central de la Qualité et de l’Information Pharmaceutiques, Hôpital de la Conception, AP-HM, 13005 Marseille, France; 3 UMR 152 PHARMA-DEV, IRD, UPS, Université de Toulouse, 31062 Toulouse, France; 4 IHU Méditerranée Infection, UMR VITROME-Tropical Eukaryotic Pathogens, Aix Marseille University, 19–21 Boulevard Jean Moulin, 13005 Marseille, France; 5 CNRS, UPS, LCC-CNRS, Université de Toulouse, 31077 Toulouse, France; 6 UMR Inserm 1094, Neuroépidémiologie Tropicale, Faculté de Pharmacie, Université de Limoges, 2 Rue Du Dr. Marcland, 87025 Limoges, France; 7 UNICAEN, CERMN, Normandie University, 14000 Caen, France; 8 Service de Pharmacie, CHU de Nîmes, 30029 Nîmes, France

**Keywords:** Imidazo[1,2-*a*]pyridine, nitroaromatic, nitroreductases, *Leishmania* spp., structure-activity relationships, thermodynamic solubility, microsomal stability, gastrointestinal permeability

## Abstract

An antileishmanial structure–activity relationship (SAR) study focused on positions 2 and 8 of the imidazo[1,2-*a*]pyridine ring was conducted through the synthesis of 22 new derivatives. After being screened on the promatigote and axenic amastigote stages of *Leishmania donovani* and *L. infantum,* the best compounds were tested against the intracellular amastigote stage of *L. infantum* and evaluated regarding their in vitro physicochemical and pharmacokinetic properties, leading to the discovery of a new antileishmanial6-chloro-3-nitro-8-(pyridin-4-yl)-2-[(3,3,3-trifluoropropylsulfonyl)methyl]imidazo[1,2-*a*]pyridine hit. It displayed low cytotoxicities on both HepG2 and THP1 cell lines (CC_50_ > 100 µM) associated with a good activity against the intracellular amastigote stage of *L. infantum* (EC_50_ = 3.7 µM *versus* 0.4 and 15.9 µM for miltefosine and fexinidazole, used as antileishmanial drug references). Moreover, in comparison with previously reported derivatives in the studied series, this compound displayed greatly improved aqueous solubility, good mouse microsomal stability (T_1/2_ > 40 min) and high gastrointestinal permeability in a PAMPA model, making it an ideal candidate for further in vivo studies on an infectious mouse model.

## 1. Introduction

Neglected tropical diseases (NTDs) are defined by the World Health Organization (WHO) as a heterogeneous group of infections mainly found in developing countries, especially in tropical and subtropical environments, and are of little interest to the pharmaceutical industry [1,2]. Among these NTDs, leishmaniases are vector-borne parasitic diseases caused by several species of flagellated protozoa of the genus *Leishmania*. More than 1 billion people in 98 countries are at risk of infection, and nearly 1 million new cases occur annually [3]. There are three main forms of the disease, cutaneous leishmaniasis (CL), mucocutaneous leishmaniasis (MCL) and visceral leishmaniasis (VL), mainly affecting populations associated with poor living conditions, malnutrition, lack of resources and a weakened immune system (*Leishmania*-HIV co-infection). With an estimated 50,000–90,000 new cases each year, life-threatening VL, caused by *Leishmania donovani* in Asia and Africa and *L*. *infantum* in the Mediterranean Basin and Latin America, is the most serious form of the disease, fatal within 2 years if left untreated and which causes more than 30,000 deaths annually [4]. Unfortunately, in the absence of a vaccine [5], treatment of VL is exclusively based on a small number of drugs. Meglumine antimoniate (Glucantime^®^) and sodium stibogluconate (Pentostam^®^), two pentavalent antimonials, are still used in East Africa as cheap treatments for visceral leishmaniasis [6]. However, the emergence of resistance in India associated with major cardiotoxicity, nephrotoxicity and hepatotoxicity, considerably limit their applications [7,8]. Amphotericin B has been used as an antileishmanial drug for many years, even though it can be responsible for severe side effects such as nephrotoxicity [9]. The liposomal form of amphotericin B (Ambisome^®^) shows a reduced renal toxicity and is very effective, but is also very expensive and requires parenteral administration [10]. Miltefosine is the only oral drug available for treating VL; it is rather well tolerated, apart some gastrointestinal side effects, but displays teratogenic properties leading to main use restrictions [11]. Moreover, only six new chemical entities have reached clinical trials in phases I or II, according to the DNDi portfolio [12]. Thus, new effective, safe and inexpensive oral antileishmanial drugs are urgently needed to strengthen the antileishmanial drug-pipeline.

**Figure 1:**
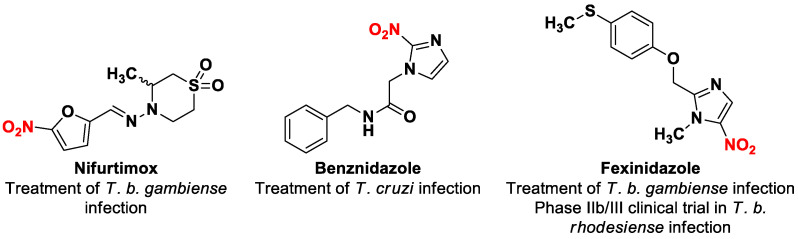
Structures of nitroheterocyclic drug-compounds that are substrates of the parasitic type 1 nitroreductases.

Nitroaromatic compounds have been used as anti-infective agents for more than 70 years, especially targeting anaerobic bacteria (but also *M. tuberculosis*) and numerous protozoan parasites (*Entamoeba* spp., *Giardia* spp.*, Trichomonas* spp., *Leishmania* spp., *Trypanosoma* spp.). Indeed, nifurtimox and benznidazole, respectively a 5-nitrofuran and a 2-nitroimidazole, have been the only two available treatments against Chagas disease since the 1960s (Figure 1) [13]. More recently, fexinidazole, a 5-nitroimidazole, became the first all-oral treatment against human African trypanosomiasis due to *Trypanosoma brucei gambiense* [14,15], leading to a renewed interest in the development of new nitroheterocyclic compounds targeting kinetoplastids such as *Leishmania*. Anti-infective nitroheterocycles act as prodrugs that need to be bioactivated by the parasitic type 1 nitroreductases (NTRs) [16,17]. This enzyme, essential for *Leishmania* and *Trypanosoma* but absent in mammalian cells, can catalyze the reduction in the nitro group in the parasite, producing nitroso and hydroxylamine metabolites which are reactive entities able to form covalent adducts with nucleophilic species such as DNA, leading to the death of the parasite cell [18]. However, a lack of crystal structure for kinetoplastid NTRs and their low degree of homology with bacterial NTRs, rule out the molecular docking approach, thereby limiting rational drug design of new nitro drugs. For these reasons, phenotypic screening and hit-to-lead development remain key to renew the therapeutic arsenal.

In order to develop new original nitroheterocyclic antikinetoplastid molecules, our team identified a first antileishmanial hit compound in 8-halogeno-3-nitroimidazo[1,2-*a*]pyridine series (Hit **A**) [19]. Introduction of a 4-chlorophenylthioether moiety at position 8 led to a new derivative (Hit **B**) with improved in vitro antileishmanial activity but showing a poor aqueous solubility (thermodynamic solubility = 1.4 µM) and a poor mouse liver microsomal stability (T_1/2_ = 3 min) (Table 1) [20]. Its sulfoxide and sulfone metabolites were identified and appeared active, but were also rapidly metabolized in vitro (T_1/2_ = 3 min). The *para* position of the phenyl ring at position 2 was also identified as potentially oxidized.

With a view to identify an optimal antileishmanial derivative which could reach in vivo studies, we explored the structure–activity relationships, investigating the influence of the substituents at position 2 of the 3-nitroimidazo[1,2-*a*]pyridine scaffold, including structures of probable oxidized metabolites and by synthesizing analogues bearing analogues with metabolic blockers at *para* position. Attempts were also made to replace the sulfone group by a sulfoximine group.

## 2. Results and Discussion

### 2.1. Synthesis

**Scheme 1:**
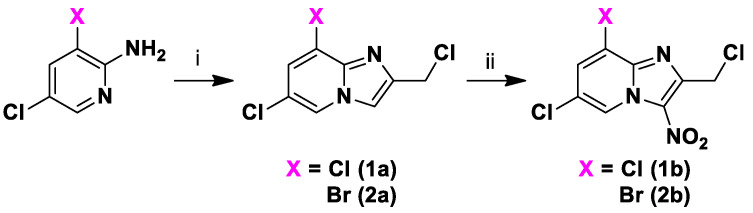
Synthesis of compounds **1a**,**b** to **2a**,**b**. Reagents and conditions: (i) 1,3-Dichloroacetone 2 equivalent, EtOH, 80 °C, 59–60%; (ii) HNO_3_ 68% 6 equivalent, H_2_SO_4_, 0 °C→RT, 60–91%.

Using a previously reported synthetic route developed in our lab [16,17], intermediates **1a** and **2a**, were obtained by the cyclocondensation of 1,3-dichloroacetone with commercially available 2-amino-5-chloropyridine in refluxing ethanol, followed by a nitration reaction at position 3, giving the 2-chloromethyl-3-nitro-8-halogeno-imidazo[1,2-*a]*pyridines **1b** and **2b** (Scheme 1).

**Scheme 2:**
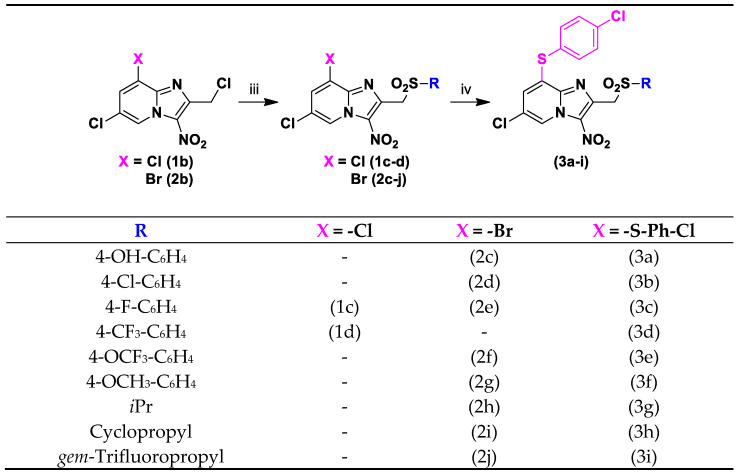
Synthesis of compounds **1c**-**d**, **2c**-**j** and **3a**-**i**. Reagents and conditions: (iii) Sodium sulfinate 3 equivalent, DMSO, RT, 15 h, 42–94%; (iv) 4-Chlorothiophenol 2 equivalent, NaH 60% 2 equivalent, DMSO, N_2_, RT, 15 h, 35–94%.

Key substrates **1b** and **2b** were engaged in S_N_2 reactions with appropriate sodium sulfinates to obtain 2-sulfonylmethyl-3-nitro-8-halogeno-[1,2-*a*]pyridine derivatives **1c**-**d** and **2c**-**j** (Scheme 2). The *para* position of the phenyl ring at position 2 was modulated with a hydroxyl group to obtain the probable oxidized metabolite **2c**, and with metabolic blockers such as chlorine and fluorine atoms (compounds **1c**–**d** and **2d**–**g**) to prevent the microsomal oxidation of CYP450 at this position. Moreover, non-aromatic substituents were introduced instead of the phenyl ring (compounds **2h**–**j**). Finally, nine derivatives **3a**–**i** bearing a 4-chlorophenylthioether moiety at position 8 were obtained, using a protocol similar to those previously described.

**Scheme 3:**
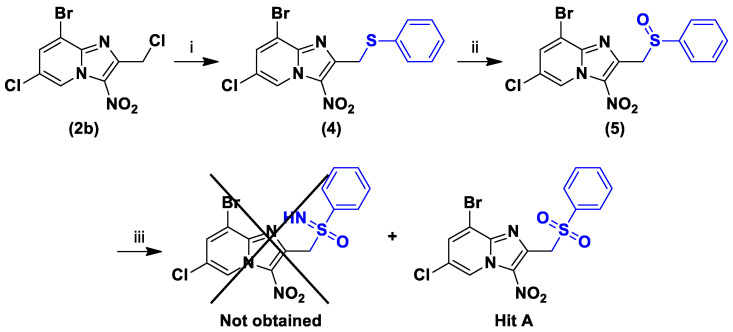
Synthesis of compounds **4** to **5**. Reagents and conditions: (i) Thiophenol 1 equivalent, NaH 1.1 equivalent, DMSO, N_2_, RT, 45 min, 60%; (ii) *m*-CPBA 1 equivalent, DCM, 0 °C, 1 h, 58%; (iii) NaN_3_ 2 equivalent, Eaton’s reagent, 50 °C, 4 h.

The increasingly common incorporation of sulfoximine in antineoplastic and anti-infective agents in clinical trials often enhances potency and improves both physicochemical and pharmacokinetic properties [21]. The attempts to synthesize new sulfoximine analogues either by starting from sulfide substrate **4**, using phenyliodine diacetate and ammonium carbamate [22], or by starting from sulfoxide substrate **5**, using sodium azide and Eaton’s reagent [23], were not successful. Only the sulfone derivative (corresponding to Hit **A**) was obtained with the second reaction instead of the sulfoximine, because of sulfur’s strong propensity to be oxidized (Scheme 3).

**Scheme 4:**
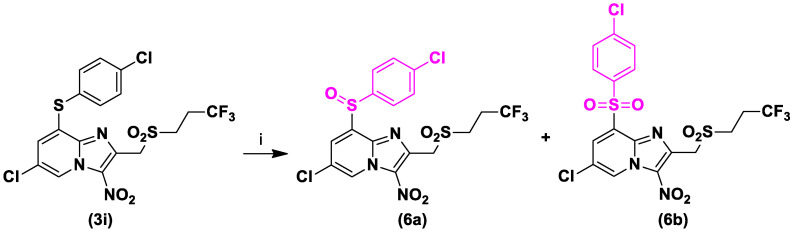
Synthesis of compounds **6a** and **6b**. Reagents and conditions: (i) *m*-CPBA 2 equivalent, DCM, RT, 15 h, 44% and 33%, respectively.

Previous works showed that the sulfur atom of the 4-chlorophenylthioether moiety was easily oxidized by CYP450 [20]. As compound **3i** showed improved, but not yet sufficient, microsomal stability, sulfoxide **6a** and sulfone **6b** metabolites were then synthesized by partial and complete oxidation of the sulfur atom, in the same reaction, using *m*-CPBA at room temperature (Scheme 4).

**Scheme 5:**
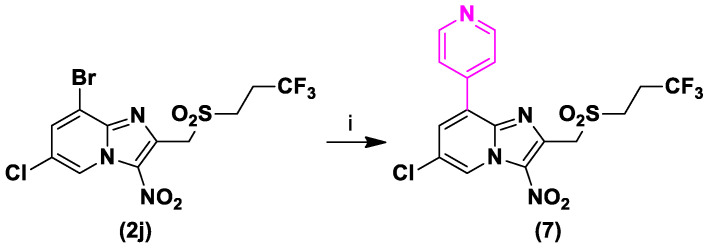
Synthesis of compound **7**. Reagents and conditions: (i) 4-Pyridinylboronic acid 1.5 equivalent, Pd(dppf)Cl_2_ 0.1 equivalent, K_2_CO_3_ 5 equivalent, THF, N_2_, 120 °C, MW, 1 h, 80%.

Finally, in order to improve microsomal stability, aqueous solubility and gastrointestinal permeability, we replaced the 4-chlorophenylthioether moiety by a pyridin-4-yl substituent at position 8 of the *gem*-trifluoropropyl derivative. This group was already introduced at this position before, on a 2-phenylsulfonylmethyl derivative [24], showing poor, but 2 times better aqueous solubility than Hit **B** (thermodynamic solubility = 3.3 µM), good mouse microsomal stability (T_1/2_ > 40 min) and high gastrointestinal permeability in a PAMPA model (PAMPA GIT = 452 nm/s). Under conditions adapted from a previously reported Suzuki-Miyaura coupling reaction in our lab, compound **7** was prepared in good yield starting from **2j** in presence of 4-pyridinylboronic acid, Pd(dppf)Cl_2_ as catalyst and potassium carbonate as base, under microwave (MW) heating, in a sealed tube (Scheme 5).

### 2.2. Biological Results

#### 2.2.1. Antileishmanial Structure-Activity Relationships Study

The antileishmanial activities of the twenty-two new derivatives synthesized were evaluated in vitro against the promastigote form of *L. donovani* and against both the promastigote and axenic amastigote forms of *L. infantum* (measured through effective concentration 50% = EC_50_). Their influence on cell viability (cytotoxic concentration 50% = CC_50_) was assessed on the HepG2 and THP1 cell lines, using doxorubicin as a positive control. The best compounds were also assessed in vitro against the intramacrophage (intra-THP1) amastigote form of *L. infantum*. Selectivity indices (SI) were calculated as SI = CC_50_/EC_50_, for both HepG2 and THP1 cell lines. Their antileishmanial activities were compared to the ones of previous hit molecules (Hit **A** and Hit **B**) and to reference drugs (amphotericin B, miltefosine, fexinidazole and fexinidazole sulfone). For all molecules, redox potentials (E_0_) of the nitro group were measured using cyclic voltammetry, and the values were corrected versus Normal Hydrogen Electrode (NHE). Weighted clogP were also computed by the SwissADME online tool [25] (Table 2).

Of the ten 8-halogeno derivatives tested, hydroxyl derivative **2c** and some of the analogues with metabolic blockers **1c**, **1d** and **2e** showed poor solubility in the culture media and a loss of activity against the axenic amastigote form of *L. infantum*. Compound **2d**, with a chlorine atom, showed good EC_50_ values on the promastigote form of *L. donovani* and against both the promastigote and axenic amastigote forms of *L. infantum*, but at the cost of a loss of solubility in the tested culture media. Derivative **2g** with a methoxy group displayed a good anti-leishmanial activity but was cytotoxic on the HepG2 cell line. Compound **2f** with a trifluoromethoxy group presented a good biological profile compared to Hit **A**, with comparable cell viability and activity against both the promastigote form of *L. donovani* and *L. infantum*, even with a better activity on the axenic amastigote form of *L. infantum* and a better solubility in the aqueous culture media. Compounds **2h** and **2i**, bearing an isopropyl- and a cyclopropyl-sulfonylmethyl group at position 2, respectively, proved to be inactive on both the promastigote form of *L. donovani* and *L. infantum*, and toward *L. infantum* axenic amastigotes. Finally, the *gem*-trifluoropropyl derivative **2j** displayed a better activity than Hit **A** against *L. infantum* promastigotes, but lower activities against *L. donovani* promastigotes and *L. infantum* axenic amastigotes. These results were promising, indicating for the first time that a compound with an alkyl substituent at this position could be active against *Leishmania* spp., since the isopropyl derivative **2h**, the cyclopropyl derivative **2i** and also the methyl derivative previously described [26] did not show any activity.

All new 2-subsituted derivatives bearing a 4-chlorophenylthioether moiety at position 8 showed good EC_50_ values, similar to or even better than Hit **B** (Table 2). Compound **3a**, which was confirmed to be a metabolite of Hit **B** by LC-MS/MS study after in vitro mouse microsomal incubation [20], maintained its promising activity against the promastigote form of *L. donovani* and the axenic amastigote form of *L. infantum*. Furthermore, except for compounds **3b** and **3g**, all the derivatives showed satisfactory CC_50_ values on the THP1 cell line (>50 μM), indicating both a low cytotoxicity and a good solubility in the culture medium. Unfortunately, on the HepG2 cell line, all these derivatives showed lower CC_50_ values than Hit **B**, some displaying significantly increased cytotoxicity (compound **3b**) and some with a slightly increased cytotoxicity although remaining weakly cytotoxic in comparison with doxorubicine (compounds **3a**, **3g** and **3h**). Others (compounds **3c**, **3d**, **3e**, **3f**, **3i**) had poor solubility in the aqueous culture medium (compounds **3c**, **3d**, **3e**, **3f**, **3i**). In contrast to 8-halogeno derivatives, compounds **3g** and **3h**, with the isopropyl- and the cyclopropyl-sulfonylmethyl group, respectively, appeared to be active on the promastigote form of *L. donovani* and against both the promastigote and axenic amastigote forms of *L. infantum*. Finally, the sulfinyl **6a** and sulfonyl **6b** metabolites of **3i**, as well as the 4-pyridyl derivative **7,** showed a loss of activity against the promastigote and axenic amastigote forms of *L. infantum* but remained active on the promastigote form of *L. donovani*. Moreover, compound **7** showed the best CC_50_ values of this series on both HepG2 and THP1 cell lines, better than Hits **A** and **B** values. All these data confirm that the substituent at position 8 played an essential role in the antileishmanial activity. 

In order to find the most active compounds of this series, only derivatives with low cytotoxicity on the THP1 cell line, good solubility in THP1 culture medium, and displaying better activity than Hit **A** against *L. infantum* axenic amastigotes (EC_50_ < 16 µM) were tested against the intramacrophage (obtained from THP1 monocytes) amastigote form of *L. infantum* (Table 2). Compared to Hit **A** and Hit **B**, hydroxy-metabolite **3a** and trifluoromethyl-derivative **3d** showed low activities, together with low selectivity indices. While chlorine derivative **3b** was the most active compound of this series on the intracellular amastigote form of *L. infantum*, its selectivity indices were low, due to its cytotoxicity on the HepG2 cell line and to a low solubility in THP1 aqueous culture medium. The selectivity indices of derivatives **2f**, **2g**, **3b**, **3c**, **3e**, **3f**, **3h** and **3i** were roughly comparable to those of hit compounds and to fexinidazole, except on the HepG2 cell line, where their selectivity indices were below 10. The sulfinyl **6a** and sulfonyl **6b** metabolites of **3i** presented a slight loss of activity against the *L. infantum* intracellular amastigotes form, especially for **6b**, but remained active on this form. In the end, only the 4-pyridyl derivative **7** showed comparable or better selectivity indices than the hit compounds and fexinidazole, with selectivity indices on the HepG2 cell line of above 27, together with renewed activity against the intracellular amastigote form of *L. infantum*, a selectivity index on THP1 better than the ones of the two hit compounds, fexinidazole and miltefosine, and the best selectivity indices on the HepG2 cell line of all the newly synthesized molecules.

Another objective was to study how the substituent at position 2 might affect the reduction potential (E_0_) of the nitro group. The lowest the reduction potential, the lowest is the risk of the nitro group to be reduced by host enzymes and be responsible for genotoxicity. It should be noted that it was not possible to determine the E_0_ of compounds **2c** and **3a**. Unfortunately, these modifications did not significantly decrease the reduction potential of the nitro group, the values remaining quite close to those of Hit **A** (E_0_ = −0.65 V) and **B** (E_0_ = −0.63 V)**.** The variations produced by these modifications depended on the substituent at position 8: the E_0_ of the 8-halogeno derivatives was between −0.60 for compounds **1c**, **1d** and **2j** and −0.65 V for compound **2h**, that of the 4-chlorothiophenyl derivatives was between −0.56 for the trifluoromethyl- derivative **3d** and −0.68 V for the chlorine derivative **3b**, while the introduction of a 4-pyridyl group (compound **7**) led to a E_0_ of −0.61 V.

Finally, the clogP of the synthesized compounds, Hit **A**, Hit **B** and the reference drugs were computed by the SwissADME (Table 2). The antileishmanial activity of the new derivatives and clogP were compared, but no correlation was observed.

#### 2.2.2. Anti-*Trypanosoma brucei* Structure-Activity Relationship Study

Hit **A** and Hit **B** were previously evaluated in vitro on the trypomastigote blood stream form (BSF) of *Trypanosoma brucei brucei*, showing good activity (EC_50_ = 1.4 µM and EC_50_ = 0.95 µM, respectively) [20]. However, with marketing authorization for Fexinidazole in late 2018 as the first all-oral treatment for *T. b. gambiense*, the search for new antitrypanosomal molecules is of much less interest. Thus, EC_50_ values of only four derivatives were determined (Table 3).

Submicromolar activities (0.40 ≥ EC_50_ ≥ 0.89 µM) of compounds **2c**, **3a**, **3c** and **3d** were better than the one of the reference drug fexinidazole, and better than those of Hit **A** and Hit **B**, evidence that a *para*-substituent on the phenyl ring improved antitrypanosomal activity for both 8-bromo and 8-phenylthio derivatives. However, as seen before, **2c**, **3c** and **3d** showed low solubility in HepG2 aqueous culture medium, restricting their selectivity indices.

#### 2.2.3. In Vitro Physicochemical and Pharmacokinetic Study of Selected Key Compounds

In vitro physicochemical and pharmacokinetic properties of selected key compounds were evaluated (Table 4). First, thermodynamic aqueous solubility at pH = 7.4 was measured. Despite good solubility values in aqueous culture media, Hit **A** (thermodynamic solubility = 6.9 µM) and especially Hit **B** (thermodynamic solubility = 1.4 µM) showed low thermodynamic solubility at pH = 7.4. Of all the synthesized derivatives, only three compounds showed a good thermodynamic aqueous solubility (>10 µM): compounds **3i** and **7** both with a *gem*-trifluoropropyl chain and bearing a 4-chlorophenylthioether moiety and a 4-pyridyl group at position 8, respectively (thermodynamic solubility = 12.4 µM and 31.1 µM, respectively), and compound **3e** with a trifluoromethoxy group (thermodynamic solubility = 64.7 µM). As expected, the replacement of the 4-chlorophenylthioether moiety at position 8 by a 4-pyridyl group resulted in an improved thermodynamic solubility, which is correlated to the solubility values obtained in HepG2 and THP1 culture media. Surprisingly, the substitution of the *para* position on the phenyl ring by a trifluoromethoxy group also greatly improved solubility compared to Hit **B** without any substituent and compared to compound **3d** bearing a trifluoromethyl group.

In vitro mouse microsomal stability (Table 4) did not appear to be significantly improved by introducing metabolic blockers (compounds **3b** and **3c**, T_1/2_ = 4.5 min and 5.7 min, respectively) when compared to Hit **B** (T_1/2_ = 3.0 min), which suggested that other phase 1 metabolism reactions may be involved, such as oxidation at other positions of the phenyl ring. Fortunately, the replacement of the phenyl ring at position 2 by the *gem*-trifluoropropyl chain (compound **3i**) resulted in a slight improvement in microsomal stability (T_1/2_ = 9.1 min); *gem*-Trifluoropropyl derivative **6b**, with complete oxidation of the sulfur atom of the 4-chlorophenylthioether moiety, led to poor microsomal stability (T_1/2_ = 7.9 min), implying that other positions were metabolized, probably on the 4-chlorophenyl ring. In the end, replacement of metabolically labile phenylthioether moiety by the electron-deficient pyridine (compound **7**) addressed the metabolic liability (T_1/2_ > 40 min).

In addition to water solubility and microsomal stability issues with hit compounds **A** and **B**, permeability across the gastrointestinal tract, a critical parameter in oral formulations, presented another challenge. Despite Hit **A**’s good permeability (PAMPA GIT = 173.2 nm/s), the introduction of the 4-chlorophenylthioether moiety (Hit **B**) led to a significant permeability decrease (PAMPA GIT = 4.0 nm/s). Modifying position 2 did not improve these values (compounds **3i**, PAMPA GIT = 6.3 nm/s). Fortunately, the 4-pyridine derivative **7** showed very good permeability (twice as good as Hit **A** and 100 times better than Hit **B**), confirming that 4-chlorophenylthioether is a restraining moiety for both microsomal stability and gastrointestinal permeability.

The last pharmacokinetic parameter investigated was the percentage of human albumin binding. This parameter was not the most critical parameter (many drugs highly bound to albumin (>99%) were marketed [27]) but it was worth trying to decrease albumin binding, to increase the proportion of the unbound fraction exerting pharmacological activity. Once again, the introduction of a 4-pyridyl group led to a significant decrease in the percentage of albumin binding from 99.8% for compound **3i** to 88.6% for compound **7**.

To be considered as an antileishmanial hit, a molecule must meet several criteria which were defined by a panel of drug experts convened by the Japanese Global Health Innovative Technology (GHIT) Fund [28]. Thus, compound **7**, by validating all key hit selection criteria, appeared as a new antileishmanial hit compound (Table 5).

## 3. Materials and Methods

### 3.1. Chemistry

#### 3.1.1. General

Melting points were determined on a Köfler melting point apparatus (Wagner & Munz GmbH, München, Germany) and were uncorrected. Elemental analyses were carried out at the Spectropole, Faculté des Sciences de Saint-Jérôme (Marseille) with a Thermo Finnigan EA1112 analyzer (Thermo Finnigan, San Jose, CA, USA). NMR spectra were recorded on a Bruker Avance NEO 400MHz NanoBay spectrometer at the Faculté de Pharmacie of Marseille or on a Bruker Avance III nanobay 400 MHz spectrometer at the Spectropole, Faculté des Sciences de Saint-Jérôme (Marseille). (^1^H NMR: reference CHCl_3_ δ = 7.26 ppm, reference DMSO-*d*_6_ δ = 2.50 ppm and ^13^C NMR: reference CHCl_3_ δ = 76.9 ppm, reference DMSO-*d*_6_ δ = 39.52 ppm). The following adsorbent was used for column chromatography: silica gel 60 (Merck KGaA, Darmstadt, Germany, particle size 0.063–0.200 mm, 70–230 mesh ASTM). TLC was performed on 5 cm x 10 cm aluminum plates coated with silica gel 60F-254 (Merck) in an appropriate eluent. Visualization was performed with ultraviolet light (234 nm). Purity determination of synthesized compounds was checked by LC/MS analyses, which were realized at the Faculté de Pharmacie of Marseille with a Thermo Scientific Accela High Speed LC System^®^ (Waltham, MA, USA) coupled using a single quadrupole mass spectrometer Thermo MSQ Plus^®^ (Waltham, MA, USA). The purity of all the synthesized compounds was > 95%, except for compounds **3c**, **6a** and **6b** for which the purity was > 90%. The RP-HPLC column is a Thermo Hypersil Gold^®^ (Waltham, MA, USA) 50 × 2.1 mm (C_18_ bounded), with particles of a diameter of 1.9 mm. The volume of sample injected on the column was 1 µL. Chromatographic analysis, total duration of 8 min, was on the gradient of the following solvents: t = 0 min, methanol/water 50:50; 0 < t < 4 min, linear increase in the proportion of methanol to a methanol/water ratio of 95:5; 4 < t < 6 min, methanol/water 95:5; 6 < t < 7 min, linear decrease in the proportion of methanol to return to a methanol/water ratio of 50:50; 6 < t < 7 min, methanol/water 50:50. The water used was buffered with ammonium acetate 5 mM. The flow rate of the mobile phase was 0.3 mL/min. The retention times (tR) of the molecules analyzed were indicated in min. Microwave reactions were performed using monomode reactors: Biotage Initiator^®^ classic (Uppsala, Sweden) in sealed vials with a power output of 0 to 400 W. Reagents were purchased from Sigma-Aldrich or Fluorochem and used without further purification. Compound **4** was synthesized according to the previous reported procedure and its physical characteristics agreed with the published data [26]. Copies of ^1^H and ^13^C NMR spectra are available in the Supplementary Materials.

#### 3.1.2. General Procedure for the Preparation of Sodium Sulfinate

Appropriate sulfonyl chloride (1 equivalent), sodium sulfite (2 equivalent) and sodium bicarbonate (2 equivalent) were dissolved in distilled water (8 mL). The reaction mixture was stirred at 80 °C for 6 h and was then cooled to room temperature. Water was removed under vacuum and the desired sodium sulfinate was obtained and used in the next step without further purification.

#### 3.1.3. General Procedure for the Preparation of 8-Chloro-2-Sulfonylmethyl[1,2-*a*]pyridine derivatives (**1c**-**1d**)

To a solution of 6,8-dichloro-2-chloromethyl-3-nitroimidazo[1,2-*a*]pyridine (**1b**) (0.5 g, 1.78 mmol, 1 equivalent) in dimethylsulfoxide (30 mL), the appropriate sodium sulfinate (5.34 mmol, 3 equivalent) was added. The reaction mixture was stirred for 15 h at room temperature.

##### 6,8-Dichloro-2-[(4-fluorophenylsulfonyl)methyl]-3-nitroimidazo[1,2-*a*]pyridine (**1c**)

Starting from sodium 4-fluorobenzenesulfinate (0.972 g), the mixture was slowly poured into an ice-water mixture and precipitated. The solid was collected by filtration and dried under reduced pressure. Compound **1c** was obtained after purification by chromatography on silica gel (eluent: dichloromethane) as a yellow solid in 42% yield (0.303 g). mp 224 °C. ^1^H NMR (400 MHz, CDCl_3_) δ: 9.41 (d, *J* = 1.6 Hz, 1H), 7.90 (dd, *J* = 8.8, 5.0 Hz, 2H), 7.74 (d, *J* = 1.6 Hz, 1H), 7.22 (d, *J* = 8.7 Hz, 2H), 5.15 (s, 2H). ^13^C NMR (101 MHz, CDCl_3_) δ: 166.4 (d, *J* = 257.5 Hz), 140.7, 139.6, 135.3 (d, *J* = 2.9 Hz), 131.7 (d, *J* = 9.7 Hz, 2C), 131.1 (2C), 125.4, 125.3, 124.4, 116.8 (d, *J* = 22.7 Hz, 2C), 56.9. LC/MS ESI+ t_R_ 1.24, (*m*/*z*) [M+H]^+^ 402.31/404.34/406.30. HRMS (+ESI): 403.9670 [M+H]^+^. Calculated for C_14_H_9_Cl_2_FN_3_O_4_S: 403.9669.

##### 6,8-Dichloro-3-Nitro-2-[(4-Trifluoromethylphenylsulfonyl)methyl]imidazo[1,2-*a*]pyridine (**1d**)

Starting from sodium 4-trifluoromethylbenzenesulfinate (1.24 g), the mixture was slowly poured into an ice-water mixture and precipitated. The solid was collected by filtration and dried under reduced pressure. Compound **1d** was obtained after purification by chromatography on silica gel (eluent: dichloromethane) as a white solid in 46% yield (0.373 g). mp 196 °C. ^1^H NMR (400 MHz, DMSO-*d*_6_) δ: 9.32 (d, *J* = 1.8 Hz, 1H), 8.33 (d, *J* = 1.8 Hz, 1H), 7.99 (q, *J* = 8.7 Hz, 4H), 5.38 (s, 2H).^13^C NMR (100 MHz, DMSO-*d*_6_) δ: 142.3, 140.0, 138.8, 133.8 (q, *J* = 32.4 Hz), 131.8, 131.2, 129.7 (2C), 126.4 (d, *J* = 3.6 Hz, 2C), 125.2, 123.4 (q, *J* = 273.1 Hz), 123.5, 123.1, 55.6. LC/MS ESI+ t_R_ 3.13, (*m*/*z*) [M+H]^+^ 453.84/455.78/457.76. HRMS (+ESI): 453.9638 [M+H]^+^. Calculated for C_15_H_9_Cl_2_F_3_N_3_O_4_S: 453.9637.

#### 3.1.4. General Procedure for the Preparation of 8-Bromo-2-Sulfonylmethyl[1,2-a]pyridine derivatives (**2c**-**2j**)

To a solution 8-bromo-6-chloro-2-chloromethyl-3-nitroimidazo[1,2-*a*]pyridine (**2b**) (0.5 g, 1.54 mmol, 1 equivalent) in dimethylsulfoxide (30 mL), the appropriate sodium sulfinate (4.62 mmol, 3 equivalent) was added. The reaction mixture was stirred for 15 h at room temperature.

##### 4-{[(8-Bromo-6-Chloro-3-Nitroimidazo[1,2-*a*]pyridin-2-yl)methyl]sulfonyl}phenol (**2c**)

Starting from sodium 4-acetoxybenzenesulfinate (0.832 g), the mixture was slowly poured into an ice-water mixture and precipitated. The solid was collected by filtration and dried under reduced pressure to give the product **2c** as a yellow solid in 70% yield (0.478 g). mp 242 °C. ^1^H NMR (400 MHz, DMSO-*d*_6_) δ: 10.64 (s, 1H), 9.34 (d, *J* = 1.7 Hz, 1H), 8.43 (d, *J* = 1.7 Hz, 1H), 7.54 (d, *J* = 8.8 Hz, 2H), 6.87 (d, *J* = 8.8 Hz, 2H), 5.14 (s, 2H). ^13^C NMR (101 MHz, DMSO-*d*_6_) δ: 162.5, 140.7, 139.7, 134.0, 131.7, 130.6 (2C), 128.4, 125.4, 123.6, 115.6 (2C), 112.0, 56.3. LC/MS ESI+ t_R_ 1.55, (*m*/*z*) [M+H]^+^ 445.59/447.72/449.62. HRMS (+ESI): 447.9184 [M+H]^+^. Calculated for C_14_H_10_BrClN_3_O_5_S: 447.9186.

##### 8-Bromo-6-Chloro-2-[(4-Chlorophenylsulfonyl)methyl]-3-Nitroimidazo[1,2-*a*]pyridine (**2d**)

Starting from sodium 4-chlorobenzenesulfinate (0.917 g), the mixture was slowly poured into an ice-water mixture and precipitated. The solid was collected by filtration and dried under reduced pressure. Compound **2d** was obtained after purification by chromatography on silica gel (eluent: cyclohexane-dichloromethane 3:7) as a white solid in 52% yield (0.372 g). mp 222 °C. ^1^H NMR (400 MHz, DMSO-*d*_6_) δ: 9.35 (d, *J* = 1.8 Hz, 1H), 8.43 (d, *J* = 1.8 Hz, 1H), 7.78 (d, *J* = 8.7 Hz, 2H), 7.67 (d, *J* = 8.7 Hz, 2H), 5.31 (s, 2H). ^13^C NMR (101 MHz, DMSO-*d*_6_) δ: 140.7, 139.4, 139.1, 137.4, 134.2, 131.8, 130.5 (2C), 129.4 (2C), 125.5, 123.7, 112.1, 55.8. LC/MS ESI+ t_R_ 5.65, (*m*/*z*) [M+H]^+^ 463.63/465.56/467.59. HRMS (+ESI): 465.8846 [M+H]^+^. Calculated for C_14_H_9_N_3_O_4_SBrCl_2_: 465.8845.

##### 8-Bromo-6-Chloro-2-[(4-Fluorophenylsulfonyl)methyl]-3-Nitroimidazo[1,2-*a*]pyridine (**2e**)

Starting from sodium 4-fluorobenzenesulfinate (0.842 g), the mixture was slowly poured into an ice-water mixture and precipitated. The solid was collected by filtration and dried under reduced pressure. Compound **2e** was obtained after purification by chromatography on silica gel (eluent: dichloromethane) as a white solid in 81% yield (0.559 g). mp 222 °C. ^1^H NMR (400 MHz, DMSO-*d*_6_) δ: 9.35 (d, *J* = 1.8 Hz, 1H), 8.44 (d, *J* = 1.8 Hz, 1H), 7.89–7.79 (m, 2H), 7.50–7.36 (m, 2H), 5.29 (s, 2H). ^13^C NMR (50 MHz, DMSO-*d*_6_) δ: 165.4 (d, *J* = 253.4 Hz), 140.7, 139.3, 134.9 (d, *J* = 2.8 Hz), 134.1, 131.8 (d, *J* = 10.0 Hz, 2C), 131.8, 125.5, 123.7, 116.4 (d, *J* = 22.8 Hz, 2C), 112.1, 55.9. LC/MS ESI+ t_R_ 5.31, (*m*/*z*) [M+H]^+^ 447.68/449.69/451.68. HRMS (+ESI): 449.9139 [M+H]^+^. Calculated for C_14_H_9_BrClFN_3_O_4_S: 449.9142.

##### 8-Bromo-6-Chloro-3-Nitro-2-[(4-Trifluoromethoxyphenylsulfonyl)methyl]imidazo[1,2-*a*] pyridine (**2f**)

Starting from sodium 4-trifluoromethoxybenzenesulfinate (1.15 g), the mixture was slowly poured into an ice-water mixture and precipitated. The solid was collected by filtration and dried under reduced pressure. Compound **2f** was obtained after purification by chromatography on silica gel (eluent: cyclohexane-ethyl acetate 8:2) as a beige solid in 94% yield (0.612 g). mp 178 °C. ^1^H NMR (400 MHz, DMSO-*d*_6_) δ: 9.35 (d, *J* = 1.8 Hz, 1H), 8.43 (d, *J* = 1.8 Hz, 1H), 7.97–7.85 (m, 2H), 7.64–7.52 (m, 2H), 5.34 (s, 2H). ^13^C NMR (101 MHz, DMSO-*d*_6_) δ: 152.2, 140.7, 139.1, 137.3, 134.2, 131.8, 131.4 (2C), 125.5, 123.7, 121.3 (2C), 119.8 (q, *J* = 259.6, CF_3_), 112.0, 55.7. LC/MS ESI+ t_R_ 5.31, (*m*/*z*) [M+H]^+^ 513.65/515.62/517.69. HRMS (+ESI): 515.9059 [M+H]^+^. Calculated for C_15_H_9_BrClF_3_N_3_O_5_S: 515.9060.

##### 8-Bromo-6-Chloro-2-[(4-Methoxyphenylsulfonyl)methyl]-3-Nitroimidazo[1,2-*a*]pyridine (**2g**)

Starting from sodium 4-methoxybenzenesulfinate (0.897 g), the mixture was slowly poured into an ice-water mixture and precipitated. The solid was collected by filtration and dried under reduced pressure. Compound **2g** was obtained after trituration in acetonitrile as a yellow solid in 57% yield (0.404 g). mp 226 °C. ^1^H NMR (400 MHz, DMSO-*d*_6_) δ: 9.34 (d, *J* = 1.4 Hz, 1H), 8.42 (d, *J* = 1.4 Hz, 1H), 7.68 (d, *J* = 8.8 Hz, 2H), 7.09 (d, *J* = 8.8 Hz, 2H), 5.19 (s, 2H), 3.85 (s, 3H). ^13^C NMR (101 MHz, CDCl_3_) δ: 163.6, 140.7, 139.6, 134.1, 131.8, 130.6 (2C), 130.2, 125.5, 123.6, 114.4 (2C), 112.1, 56.2, 55.9. LC/MS ESI+ t_R_ 2.46, (*m*/*z*) [M+H]^+^ 459.72/461.20/462.59. HRMS (+ESI): 461.9341 [M+H]^+^. Calculated for C_15_H_12_BrClN_3_O_5_S: 461.9342.

##### 8-Bromo-6-Chloro-2-[(isopropylsulfonyl)methyl]-3-Nitroimidazo[1,2-*a*]pyridine (**2h**)

Starting from sodium propane-2-sulfinate (0.601 g), the mixture was slowly poured into an ice-water mixture and precipitated. The solid was collected by filtration and dried under reduced pressure. Compound **2h** was obtained after purification by chromatography on silica gel (eluent: dichloromethane) as a beige solid in 80% yield. mp 183 °C (0.488 g). ^1^H NMR (400 MHz, DMSO-*d*_6_) δ: 9.39 (d, *J* = 1.8 Hz, 1H), 8.45 (d, *J* = 1.8 Hz, 1H), 5.12 (s, 2H), 3.53–3.44 (m, 1H), 1.35 (d, *J* = 6.8 Hz, 6H). ^13^C NMR (101 MHz, DMSO-*d*_6_) δ: 140.8, 139.8, 134.2, 132.1, 125.5, 123.6, 112.0, 53.2, 50.0, 14.8 (2C). LC/MS ESI+ t_R_ 1.16, (*m*/*z*) [M+H]^+^ 395.90/398.70. HRMS (+ESI): 397.9391 [M+H]^+^. Calculated for C_11_H_12_N_3_O_4_SBrCl: 397.9393.

##### 8-Bromo-6-Chloro-2-[(cyclopropylsulfonyl]methyl)-3-Nitroimidazo[1,2-*a*]pyridine (**2i**)

Starting from sodium cyclopropanesulfinate (0.592 g), the mixture was slowly poured into an ice-water mixture and precipitated. The solid was collected by filtration and dried under reduced pressure. Compound **2i** was obtained after purification by chromatography on silica gel (eluent: dichloromethane) as a beige solid in 57% yield (0.346 g). mp 250 °C. ^1^H NMR (400 MHz, DMSO-*d*_6_) δ: 9.39 (d, *J* = 1.7 Hz, 1H), 8.45 (d, *J* = 1.7 Hz, 1H), 5.17 (s, 2H), 2.98–2.78 (m, 1H), 1.15–0.87 (m, 4H). ^13^C NMR (101 MHz, DMSO-*d*_6_) δ: 140.8, 139.9, 134.1, 131.9, 125.5, 123.6, 112.0, 53.9, 30.2, 4.7 (2C). LC/MS ESI+ t_R_ 4.47, (*m*/*z*) [M+H]^+^ 393.84/395.78/397.84. HRMS (+ESI): 395.9235 [M+H]^+^. Calculated for C_11_H_10_N_3_O_4_SBrCl: 395.9236.

##### 8-Bromo-6-Chloro-3-Nitro-2-[(3,3,3-Trifluoropropylsulfonyl)methyl]imidazo[1,2-*a*]pyridine (**2j**)

Starting from sodium 3,3,3-trifluoropropane-1-sulfinate (0.851 g), the mixture was slowly poured into an ice-water mixture and precipitated. The solid was collected by filtration and dried under reduced pressure. Compound **2j** was obtained after purification by chromatography on silica gel (eluent: dichloromethane) as a pale yellow solid in 88% yield (0.610 g). mp 181 °C. ^1^H NMR (400 MHz, DMSO-*d*_6_) δ: 9.39 (d, *J* = 1.7 Hz, 1H), 8.47 (d, *J* = 1.8 Hz, 1H), 5.29 (s, 2H), 3.70–3.61 (m, 2H), 2.94–2.78 (m, 2H). ^13^C NMR (101 MHz, DMSO-*d*_6_) δ: 140.9, 139.3, 134.3, 132.0, 126.2 (q, *J* = 276.9 Hz), 125.5, 123.7, 112.0, 52.7, 45.8 (q, *J* = 2.8 Hz), 26.4 (q, *J* = 30.4 Hz). LC/MS ESI+ t_R_ 5.27, (*m*/*z*) [M+H]^+^ 449.61/451.65/453.67. HRMS (+ESI): 451.9110 [M+H]^+^. Calculated for C_11_H_9_BrClF_3_N_3_O_4_S: 451.9110.

#### 3.1.5. General Procedure for the Preparation of 8-Phenylthioimidazo[1,2-*a*]pyridine Derivatives (**3a**-**3i**)

To a sealed 20 mL flask containing sodium hydride (60% dispersion in mineral oil) (2 equivalent) in dimethylsulfoxide (2 mL), 4-chlorobenzenethiol (2 equivalent) was added under N_2_ atmosphere. The reaction mixture was stirred at room temperature for 30 min. Then a solution of appropriate 8-halogeno-6-chloro-3-nitro-2-([sulfonyl]methyl)imidazo[1,2-*a*]pyridine (**1c**-**1d**; **2c**-**2j**) (1 equivalent) in dimethylsulfoxide (10 mL) was injected. The reaction mixture was stirred for 15 h at room temperature.

##### 4-{[(6-Chloro-8-[(4-Chlorophenyl)thio]-3-Nitroimidazo[1,2-*a*]pyridin-2-yl)methyl]sulfonyl}phenol (**3a**)

Starting from compound **2c** (0.2 g), the mixture was slowly poured into an ice-water mixture. The mixture was extracted three times with ethyl acetate, then the organic layer was washed four times with brine, dried over anhydrous Na_2_SO_4_, filtered and evaporated. Compound **3a** was obtained after purification by chromatography on silica gel (eluent: dichloromethane-ethyl acetate 9:1) as an orange solid in 35% yield (0.08 g). mp 241 °C. ^1^H NMR (400 MHz, DMSO-*d*_6_) δ: 10.64 (s, 1H), 9.14 (d, *J* = 1.7 Hz, 1H), 7.70–7.61 (m, 4H), 7.55 (d, *J* = 8.8 Hz, 2H), 7.01 (d, *J* = 1.7 Hz, 1H), 6.89 (d, *J* = 8.8 Hz, 2H), 5.14 (s, 2H). ^13^C NMR (101 MHz, DMSO-*d*_6_) δ: 162.5, 139.7, 139.0, 136.2 (2C), 135.2, 131.3, 130.6 (2C), 130.4 (2C), 130.1, 128.4, 127.0, 126.9, 123.9, 123.0, 115.7 (2C), 56.2. LC/MS ESI+ t_R_ 3.80, (*m*/*z*) [M+H]^+^ 509.62/511.61. HRMS (+ESI): 509.9746 [M+H]^+^. Calculated for C_20_H_14_Cl_2_N_3_O_5_S_2_: 509.9746.

##### 6-Chloro-2-[(4-Chlorophenylsulfonyl)methyl]-8-[(4-Chlorophenyl)thio]-3-Nitroimidazo[1,2-*a*]pyridine (**3b**)

Starting from compound **2d** (0.2 g), the mixture was slowly poured into an ice-water mixture. The mixture was extracted three times with ethyl acetate, then the organic layer was washed four times with brine, dried over anhydrous Na_2_SO_4_, filtered and evaporated. Compound **3b** was obtained after purification by chromatography on silica gel (eluent: cyclohexane-dichloromethane 2:8) as a yellow solid in 70% yield (0.16 g). mp 221 °C. ^1^H NMR (400 MHz, DMSO-*d*_6_) δ: 9.16 (d, *J* = 1.8 Hz, 1H), 7.81–7.75 (m, 2H), 7.69–7.65 (m, 2H), 7.63 (s, 4H), 7.04 (d, *J* = 1.8 Hz, 1H), 5.30 (s, 2H). ^13^C NMR (101 MHz, DMSO-*d*_6_) δ: 139.8, 139.4, 138.5, 137.4, 136.0 (2C), 135.1, 131.3, 130.5 (2C), 130.4 (2C), 129.9, 129.4 (2C), 127.3, 127.2, 124.1, 123.2, 55.7. LC/MS ESI+ t_R_ 6.64, (*m*/*z*) [M+H]^+^ 527.64/529.67/531.74. HRMS (+ESI): 529.9383 [M+H]^+^. Calculated for C_20_H_13_N_3_O_4_S_2_Cl_3_: 529.9380.

##### 6-Chloro-8-[(4-Chlorophenyl)thio]-2-[(4-Fluorophenylsulfonyl)methyl]-3-Nitroimidazo[1,2-*a*]pyridine (**3c**)

Starting from compound **1c** (0.2 g), the mixture was slowly poured into an ice-water mixture. The mixture was extracted three times with ethyl acetate, then the organic layer was washed four times with brine, dried over anhydrous Na_2_SO_4_, filtered and evaporated. Compound **3c** was obtained after purification by chromatography on silica gel (eluent: dichloromethane) as a yellow solid in 56% yield (0.142 g). mp 197 °C. ^1^H NMR (400 MHz, CDCl_3_) δ: 9.18 (d, *J* = 1.7 Hz, 1H), 7.92 (dd, *J* = 8.8, 5.0 Hz, 2H), 7.55 (dt, *J* = 10.7, 7.5 Hz, 4H), 7.23 (d, *J* = 8.6 Hz, 2H), 6.76 (d, *J* = 1.7 Hz, 1H), 5.16 (s, 2H). ^13^C NMR (100 MHz, CDCl_3_) δ: 166.3 (d, *J* = 257.2 Hz), 139.9, 138.8, 137.6, 137.2 (2C), 135.4 (d, *J* = 3.1 Hz), 133.2, 131.8 (d, *J* = 9.7 Hz, 2C), 131.0 (2C), 130.3, 126.2, 126.1, 125.9, 121.8, 116.7 (d, *J* = 22.7 Hz, 2C), 56.9. LC/MS ESI+ t_R_ 4.23, (*m*/*z*) [M+H]^+^ 512.63/514.39/516.79. HRMS (+ESI): 511.9703 [M+H]^+^. Calculated for C_20_H_13_Cl_2_FN_3_O_4_S_2_: 511.9703.

##### 6-Chloro-8-[(4-Chlorophenyl)thio]-3-Nitro-2-[(4-Trifluoromethylphenylsulfonyl)methyl]imidazo[1,2-*a*]pyridine (**3d**)

Starting from compound **1d** (0.2 g), the mixture was slowly poured into an ice-water mixture. The mixture was extracted three times with ethyl acetate, then the organic layer was washed four times with brine, dried over anhydrous Na_2_SO_4_, filtered and evaporated. Compound **3d** was obtained after purification by chromatography on silica gel (eluent: cyclohexane-dichloromethane 1:9) as a yellow solid in 63% yield (0.156 g). mp 222 °C. ^1^H NMR (400 MHz, DMSO-*d*_6_) δ: 9.16 (d, *J* = 1.4 Hz, 1H), 8.11–7.90 (m, 4H), 7.68–7.56 (m, 4H), 7.01 (d, *J* = 1.4 Hz, 1H), 5.38 (s, 2H). ^13^C NMR (101 MHz, DMSO-*d*_6_) δ: 142.4, 139.7, 138.2, 136.0 (2C), 135.1, 133.7 (q, *J* = 31.9 Hz), 131.4, 130.5 (2C), 129.9, 129.6 (2C), 127.3, 127.1, 126.4 (d, *J* = 3.7 Hz, 2C), 124.1, 123.4 (d, *J* = 273.2 Hz), 123.2, 55.5. LC/MS ESI+ t_R_ 4.65, (*m*/*z*) [M+H]^+^ 561.69/563.80/565.83. HRMS (+ESI): 561.9675 [M+H]^+^. Calculated for C_21_H_13_Cl_2_F_3_N_3_O_4_S_2_: 561.9671.

##### 6-Chloro-8-[(4-Chlorophenyl)thio]-3-Nitro-2-[(4-Trifluoromethoxyphenylsulfonyl)methyl]imidazo[1,2-*a*]pyridine (**3e**)

Starting from compound **2f** (0.2 g), the mixture was slowly poured into an ice-water mixture and precipitated. The solid was collected by filtration and dried under reduced pressure. Compound **3e** was obtained after purification by chromatography on silica gel (eluent: cyclohexane-ethyl acetate 8:2) as a yellow solid in 94% yield (0.211 g). mp 173 °C. ^1^H NMR (400 MHz, DMSO-*d*_6_) δ: 9.15 (d, *J* = 1.7 Hz, 1H), 7.92 (d, *J* = 8.8 Hz, 2H), 7.67–7.53 (m, 6H), 7.01 (d, *J* = 1.7 Hz, 1H), 5.34 (s, 2H). ^13^C NMR (101 MHz, DMSO-*d*_6_) δ: 152.2, 139.7, 138.5, 137.4, 136.0 (2C), 135.2, 131.4, 131.3 (2C), 130.5 (2C), 130.0, 127.2, 127.1, 124.1, 123.1, 121.3 (2C), 119.8 (d, *J* = 258.6 Hz), 55.7. LC/MS ESI+ t_R_ 6.76, (*m*/*z*) [M+H]^+^ 577.75/579.77. HRMS (+ESI): 577.9620 [M+H]^+^. Calculated for C_21_H_13_Cl_2_F_3_N_3_O_5_S_2_: 577.9620.

##### 6-Chloro-8-[(4-Chlorophenyl)thio]-2-[(4-Methoxyphenylsulfonyl)methyl]-3-Nitroimidazo[1,2-*a*]pyridine (**3f**)

Starting from compound **2g** (0.2 g), the mixture was slowly poured into an ice-water mixture. The mixture was extracted three times with ethyl acetate, then the organic layer was washed four times with brine, dried over anhydrous Na_2_SO_4_, filtered and evaporated. Compound **3f** was obtained after purification by chromatography on silica gel (eluent: dichloromethane-ethyl acetate 9:1) as a yellow solid in 44% yield (0.100 g). mp 214 °C. ^1^H NMR (400 MHz, DMSO-*d*_6_) δ: ^1^H NMR (400 MHz, DMSO) δ 9.15 (d, *J* = 1.7 Hz, 1H), 7.71–7.60 (m, 6H), 7.10 (d, *J* = 8.9 Hz, 2H), 7.02 (d, *J* = 1.7 Hz, 1H), 5.19 (s, 2H), 3.85 (s, 3H). ^13^C NMR (101 MHz, DMSO-*d*_6_) δ: 163.6, 139.7, 138.9, 136.1 (2C), 135.2, 131.4, 130.5 (2C), 130.4 (2C), 130.3, 130.0, 127.1 (2C), 124.0, 123.1, 114.5 (2C), 56.1, 55.8. LC/MS ESI+ t_R_ 4.39, (*m*/*z*) [M+H]^+^ 523.74/524.50/527.76. HRMS (+ESI): 523.9903 [M+H]^+^. Calculated for C_21_H_16_Cl_2_N_3_O_5_S_2_: 523.9903.

##### 6-Chloro-8-[(4-Chlorophenyl)thio]-2-[(isopropylsulfonyl)methyl]-3-Nitroimidazo[1,2-*a*]pyridine (**3g**)

Starting from compound **2h** (0.2 g), the mixture was slowly poured into an ice-water mixture and precipitated. The solid was collected by filtration and dried under reduced pressure. Compound **3g** was obtained after purification by chromatography on silica gel (eluent: cyclohexane-dichloromethane 8:2) as a yellow solid in 67% yield (0.156 g). mp 208 °C. ^1^H NMR (400 MHz, DMSO-*d*_6_) δ: 9.25 (d, *J* = 1.8 Hz, 1H), 7.62–7.50 (m, 4H), 7.40 (d, *J* = 1.8 Hz, 1H), 5.07 (s, 2H), 3.22 (dt, *J* = 13.6, 6.8 Hz, 1H), 1.26 (t, *J* = 7.0 Hz, 6H). ^13^C NMR (101 MHz, DMSO-*d*_6_) δ: 140.3, 139.3, 134.6 (2C), 134.2, 131.6, 130.1 (2C), 129.8, 128.7, 128.0, 124.1, 123.8, 52.4, 49.8, 14.6 (2C). LC/MS ESI+ t_R_ 6.17, (*m*/*z*) [M+H]^+^ 459.81/461.84/463.75. HRMS (+ESI): 459.9952 [M+H]^+^. Calculated for C_17_H_16_N_3_O_4_S_2_Cl_2_: 459.9954.

##### 6-Chloro-8-[(4-Chlorophenyl)thio]-2-[(cyclopropylsulfonyl)methyl]-3-Nitroimidazo[1,2-*a*]pyridine (**3h**)

Starting from compound **2i** (0.2 g), the mixture was slowly poured into an ice-water mixture and precipitated. The solid was collected by filtration and dried under reduced pressure. Compound **3h** was obtained after purification by chromatography on silica gel (eluent: cyclohexane-dichloromethane 8:2) as a yellow solid in 70% yield (0.163 g). mp 196 °C. ^1^H NMR (400 MHz, DMSO-*d*_6_) δ: 9.24 (d, *J* = 1.8 Hz, 1H), 7.69–7.51 (m, 4H), 7.24 (d, *J* = 1.8 Hz, 1H), 5.13 (s, 2H), 2.78–2.63 (m, 1H), 1.04–0.86 (m, 4H). ^13^C NMR (101 MHz, DMSO-*d*_6_) δ: 140.1, 139.3, 135.3 (2C), 134.6, 131.5, 130.2 (2C), 128.9, 128.6, 128.0, 123.9, 123.7, 53.7, 30.0, 4.7 (2C). LC/MS ESI+ t_R_ 6.07, (*m*/*z*) [M+H]^+^ 457.77/459.78/461.60. HRMS (+ESI): 457.9797 [M+H]^+^. Calculated for C_17_H_13_N_3_O_4_S_2_Cl_2_: 457.9797.

##### 6-Chloro-8-[(4-Chlorophenyl)thio]-3-Nitro-2-[(3,3,3-Trifluoropropylsulfonyl)methyl]imidazo[1,2-*a*]pyridine (**3i**)

Starting from compound **2j** (0.2 g), the mixture was slowly poured into an ice-water mixture and precipitated. The solid was collected by filtration and dried under reduced pressure. Compound **3i** was obtained after purification by chromatography on silica gel (eluent: dichloromethane) as a yellow solid in 66% yield (0.161 g). mp 183 °C. ^1^H NMR (400 MHz, DMSO-*d*_6_) δ: 9.22 (d, *J* = 1.7 Hz, 1H), 7.62 (dd, *J* = 20.6, 8.6 Hz, 4H), 7.14 (d, *J* = 1.7 Hz, 1H), 5.28 (s, 2H), 3.68–3.56 (m, 2H), 2.93–2.76 (m, 2H). ^13^C NMR (101 MHz, DMSO-*d*_6_) δ: 140.0, 138.7, 135.7 (2C), 135.0, 131.6, 130.4 (2C), 129.5, 128.0, 127.3, 126.2 (q, *J* = 276.7 Hz), 124.1, 123.4, 52.6, 45.8 (q, *J* = 2.9 Hz), 26.3 (q, *J* = 30.3 Hz). LC/MS ESI+ t_R_ 6.44, (*m*/*z*) [M+H]^+^ 513.70/515.84/517.68. HRMS (+ESI): 513.9675 [M+H]^+^. Calculated for C_17_H_13_Cl_2_F_3_N_3_O_4_S_2_: 513.9671.

#### 3.1.6. 8-Bromo-6-Chloro-3-Nitro-2-[(phenylsulfinyl)methyl]imidazo[1,2-*a*]pyridine (**5**)

To a solution of 8-bromo-6-chloro-3-nitro-2-(phenylthiomethyl)imidazo[1,2-*a*]pyridine (**4**) (0.5 g, 1.25 mmol, 1 equivalent) in dichloromethane (40 mL) cooled by an ice-water bath, *m*-CPBA (≈70%) (0.31 g, 1.25 mmol, 1 equivalent) was added. The reaction mixture was stirred for 1 h at 0 °C. Then, the mixture was washed three times with H_2_O, dried over anhydrous Na_2_SO_4_, filtered and evaporated. Compound **5** was obtained after purification by chromatography on silica gel (eluent: dichloromethane-ethyl acetate 8:2) as a yellow solid in 58% yield (0.302 g). mp 198 °C. ^1^H NMR (400 MHz, DMSO-*d*_6_) δ: 9.35 (d, *J* = 1.8 Hz, 1H), 8.42 (d, *J* = 1.8 Hz, 1H), 7.66–7.50 (m, 5H), 4.75 (q, *J* = 31.9, 12.7 Hz, 2H). ^13^C NMR (101 MHz, DMSO-*d*_6_) δ: 143.6, 142.2, 140.8, 134.0, 131.9, 131.4, 129.2 (2C), 125.4, 124.0 (2C), 123.4, 111.8, 57.3. LC/MS ESI+ t_R_ 2.19, (*m*/*z*) [M+H]^+^ 413.66/415.82/417.72. HRMS (+ESI): 415.9279 [M+H]^+^. Calculated for C_14_H_9_BrClN_3_O_3_S: 415.9287.

#### 3.1.7. 6-Chloro-8-[(4-Chlorophenyl)sulfinyl]-3-Nitro-2-[(3,3,3-Trifluoropropylsulfonyl)methyl] imidazo[1,2-*a*]pyridine (**6a**)

To a solution of 6-chloro-8-[(4-chlorophenyl)thio]-3-nitro-2-[(3,3,3-trifluoropropylsulfonyl)methyl]imidazo[1,2-*a*]pyridine (**3i**) (0.2 g, 0.39 mmol, 1 equivalent) in dichloromethane (20 mL), *m*-CPBA (≈70%) (0.19 g, 0.78 mmol, 2 equivalent) was added. The reaction mixture was stirred for 15 h at room temperature. Then, the mixture was washed three times with H_2_O, dried over anhydrous Na_2_SO_4_, filtered and evaporated. Compound **6a** was obtained after purification by chromatography on silica gel (eluent: dichloromethane-ethyl acetate 95:5) as a white solid in 44% yield (0.09 g). mp 221 °C. ^1^H NMR (400 MHz, DMSO-*d*_6_) δ: 9.40 (d, *J* = 1.9 Hz, 1H), 8.19 (d, *J* = 1.9 Hz, 1H), 7.99–7.95 (m, 2H), 7.58–7.53 (m, 2H), 5.27 (q, *J* = 14.4 Hz, 2H), 3.66–3.49 (m, 2H), 2.93–2.78 (m, 2H). ^13^C NMR (101 MHz, DMSO-*d*_6_) δ: 141.6, 139.0, 137.8, 136.9, 135.2, 131.8, 129.5 (2C), 127.9, 127.2 (2C), 126.6, 126.2 (d, *J* = 276.5 Hz), 124.1, 52.6, 45.7 (d, *J* = 2.8 Hz), 26.2 (q, *J* = 30.4 Hz). LC/MS ESI+ t_R_ 5.89, (*m*/*z*) [M+H]^+^ 529.74/531.77. HRMS (+ESI): 529.9625 [M+H]^+^. Calculated for C_17_H_13_Cl_2_F_3_N_3_O_5_S_2_: 529.9620.

#### 3.1.8. 6-Chloro-8-[(4-Chlorophenyl)sulfonyl]-3-Nitro-2-[(3,3,3-Trifluoropropylsulfonyl)methyl] imidazo[1,2-*a*]pyridine (**6b**)

To a solution of 6-chloro-8-[(4-chlorophenyl)thio]-3-nitro-2-[(3,3,3-trifluoropropylsulfonyl)methyl]imidazo[1,2-*a*]pyridine (**3i**) (0.2 g, 0.39 mmol, 1 equivalent) in dichloromethane (20 mL), *m*-CPBA (≈70%) (0.19 g, 0.78 mmol, 2 equivalent) was added. The reaction mixture was stirred for 15 h at room temperature. Then, the mixture was washed three times with H_2_O, dried over anhydrous Na_2_SO_4_, filtered and evaporated. Compound **6b** was obtained after purification by chromatography on silica gel (eluent: dichloromethane) as a beige solid in 33% yield (0.07 g). mp 233 °C. ^1^H NMR (400 MHz, CDCl_3_) δ: 9.62 (d, *J* = 1.9 Hz, 1H), 8.47 (d, *J* = 1.9 Hz, 1H), 8.27–8.13 (m, 2H), 7.59–7.45 (m, 2H), 5.08 (s, 2H), 3.54–3.42 (m, 2H), 2.85–2.69 (m, 2H). ^13^C NMR (101 MHz, CDCl_3_) δ: 142.1, 139.6, 138.4, 136.9, 133.6, 131.4, 131.0 (2C), 129.9 (2C), 129.8, 128.3 (d, *J* = 276.8 Hz), 125.5, 53.8, 47.4 (d, *J* = 2.9 Hz), 27.3 (q, *J* = 32.0 Hz), 1C missing (C-NO_2_). LC/MS ESI+ t_R_ 5.86, (*m*/*z*) [M+H]^+^ 545.74/547.81. HRMS (+ESI): 545.9571 [M+H]^+^. Calculated for C_17_H_13_Cl_2_F_3_N_3_O_6_S_2_: 545.9569.

#### 3.1.9. 6-Chloro-3-Nitro-8-(Pyridin-4-yl)-2-[(3,3,3-Trifluoropropylsulfonyl)methyl]imidazo[1,2-*a*]pyridine (**7**)

A mixture of 8-bromo-6-chloro-3-nitro-2-[(3,3,3-trifluoropropylsulfonyl)methyl]imidazo[1,2-*a*]pyridine (**2j**) (0.2 g, 0.44 mmol, 1 equivalent), [1,1′-bis(diphenylphosphino)ferrocene]dichloropalladium(II) (32 mg, 0.044 mmol, 0.1 equivalent), potassium carbonate (0.304 g, 2.20 mmol, 5 equivalent), and 4-pyridinylboronic acid (0.081 g, 0.66 mmol, 1.5 equivalent) in THF (7 mL) under N_2_ atmosphere was heated at 120 °C under microwave irradiation for 1 h. The solvent was then evaporated *in vacuo*. Compound **7** was obtained after purification by chromatography on neutral alumina gel (eluent: dichloromethane) as a beige solid in 80% yield (0.16 g). mp 184 °C. ^1^H NMR (400 MHz, DMSO-*d*_6_) δ: 9.48 (d, *J* = 1.9 Hz, 1H), 8.73 (dd, *J* = 4.5, 1.6 Hz, 2H), 8.35 (d, *J* = 1.9 Hz, 1H), 8.03 (dd, *J* = 4.5, 1.6 Hz, 2H), 5.29 (s, 2H), 3.72–3.62 (m, 2H), 2.88 (ddd, *J* = 11.0, 8.3, 5.1 Hz, 2H). ^13^C NMR (101 MHz, DMSO-*d*_6_) δ: 149.9 (2C), 141.1, 140.6, 139.5, 131.4, 127.6, 127.5, 126.1, 124.8, 124.3, 123.7 (2C), 52.5, 45.7, 26.4 (q, *J* = 30.3 Hz). LC/MS ESI+ t_R_ 4.87, (*m*/*z*) [M+H]^+^ 448.89/450.95, purity = 99%. HRMS (+ESI): 449.0289 [M+H]^+^. Calculated for C_16_H_13_ClF_3_N_4_O_4_S: 449.0293.

### 3.2. Electrochemistry

Voltammetric measurements were carried out with a potentiostat Autolab PGSTAT100 (ECO Chemie, Utrecht, The Netherlands) controlled by GPES 4.09 software (ECO Chemie, Utrecht, The Netherlands). Experiments were performed at room temperature in a homemade airtight three–electrode cell connected to a vacuum/argon line. The reference electrode consisted of a saturated calomel electrode (SCE) separated from the solution by a bridge compartment. The counter electrode was a platinum wire of 1 cm^2^ apparent surface. The working electrode was a GC microdisk (1.0 mm in diameter–Bio-logic SAS). The supporting electrolyte (nBu_4_N)[PF_6_] (Fluka, 99% puriss electrochemical grade) and the solvent DMSO (Sigma-Aldrich puriss p.a. dried <0.02% water) were used as received and simply degassed under argon. The solutions used during electrochemical analyses were typically 10^−3^ M in compound and 0.1 M in supporting electrolyte. Before each measurement, the solutions were degassed by bubbling Ar, and the working electrode was polished with a polishing machine (Presi P230, Eybens, France). Under the experimental conditions adopted in this work, the half-wave potential (E_1/2_) of the ferrocene Fc+/Fc couple in DMSO was E_1/2_ = 0.45 V vs. SCE. Experimental peak potentials were measured versus SCE and converted to NHE by adding 0.241 V.

### 3.3. Biology

#### 3.3.1. Antileishmanial Activity against *L. donovani* Promastigotes

The *Leishmania* species used in this study were *L. donovani* (MHOM/IN/00/DEVI) purchased from CNR Leishmania (Montpellier, France). *Leishmania* promastigote forms were grown in Schneider’s Drosophila medium (Life Technologies, Saint-Aubin, France) supplemented with 100 U/mL penicillin, 100 µg/mL streptomycin, 2 mM L-glutamine and 20% FCS (Life Technologies, Saint-Aubin, France) at 27 °C. The in vitro evaluation of the tested compound’s antileishmanial activity on promastigote forms was carried out by MTT assay according to the Mosmann protocol [29] with some modifications. Briefly, promastigotes in log-phase were incubated at an average density of 10^6^ parasites/mL in sterile 96-well plates with various concentrations of compound dissolved in DMSO (final concentration less than 0.5% *v*/*v*), in duplicate. Appropriate controls treated with DMSO, Miltefosine, Amphotericin B, Fexinidazole, and Fexinidazole sulfone (reference drugs purchased from Sigma-Aldrich, Saint-Louis, Missouri, USA) were added to each set of experiments. After a 72 h incubation period at 27 °C, parasitic metabolic activity was determined. Each plate-well was then microscope-analyzed to detect any precipitate formation. 20 µL of MTT (3-(4,5-dimethylthiazol-2-yl)-2,5-diphenyltetrazolium bromide) (Sigma-Aldrich, Saint-Louis, Missouri, USA) solution (5 mg/mL in PBS) were added to each well and incubated for 4 h at 27 °C. The enzyme reaction was then stopped by addition of 100 µL of 50% isopropanol–10% sodium dodecyl sulfate. Plates were shaken vigorously at 300 rpm for 10 min. The absorbance was measured at 570 nm in a BIO-TEK Elx808 (Biotek, Colmar, France) absorbance microplate reader. Inhibitory concentration 50% (EC_50_) was defined as the concentration of drug required to inhibit the metabolic activity of *Leishmanial* promastigote forms by 50% relative to the control. EC_50_ values were calculated by non-linear regression analysis processed dose-response curves, using TableCurve 2D V5.0 software (Systat Software Inc. (Palo Alto, CA, USA)). EC_50_ values represent the mean value calculated from at least three separate experiments.

#### 3.3.2. Antileishmanial Activity against *L. infantum* Promastigotes

*L*. *infantum* promastigotes (MHOM/MA/67/ITMAP-263, CNR Leishmania, Montpellier, France, expressing luciferase activity) were used in the following tests. The effects of the tested compounds on the growth of *L. infantum* promastigotes were assessed by Luciferase Assay. Briefly, promastigotes in log-phase in RPMI 1640 medium supplemented with 10% fetal calf serum (FCS), 2 mM L-glutamine and antibiotics (100U/mL penicillin, 100 µg/mL streptomycin and 50µg/mL geneticin), were incubated at an average density of 10^6^ parasites/mL (100 µL/well) in sterile 96-well plates with various concentrations of compounds dissolved in DMSO (final concentration less than 0.5% *v*/*v*), in duplicate. Appropriate controls (100 µL/well) treated by DMSO and reference drugs (purchased for the majority from Sigma Aldrich) were added to each set of experiments. After a 72 h incubation period at 24 °C, each plate-well was then microscope-examined for detecting possible precipitate formation. To estimate the luciferase activities of promastigotes, 80 µL of each well are transferred in white 96-well plates, Steady Glow reagent (Promega) was added according to manufacter’s instructions, and plates were incubated for 2 min. The luminescence was measured in a FLUOstar Omega (BMG Labtech, Champigny-sur-Marne, France). Efficient concentration 50% (EC_50_) was defined as the concentration of drug required to inhibit by 50% the metabolic activity of *L. infantum* promastigotes compared to the control. EC_50_ were calculated by non-linear regression analysis processed on dose–response curves, using TableCurve 2D V5 software.

#### 3.3.3. Antileishmanial Activity on *L. infantum* Axenic Amastigotes

*L*. *infantum* promastigotes were harvested in logarithmic phase of growth by centrifugation at 900× *g* for 10 min. The supernatant was removed carefully and was replaced by the same volume of RPMI 1640 complete medium at pH 5.4 and incubated for 24 h at 24 °C. The acidified promastigotes were then incubated for 24 h at 37 °C in a ventilated flask to transform them into axenic amastigotes. The amastigote stage was checked both by electron microscopy (short flagellum with small bulbous tip extending beyond a spherical cell body) and RT-PCR to confirm the overexpression of ATG8 and amastin genes in amastigotes, relative to promastigotes. The effects of the tested compounds on the growth of *L*. *infantum* axenic amastigotes were assessed as follows. *L*. *infantum* amastigotes were incubated at a density of 2 × 10^6^ parasites/mL in sterile 96-well plates with various concentrations of compounds dissolved in DMSO (final concentration less than 0.5% *v*/*v*), in duplicate. Appropriate controls treated with DMSO, amphotericin B, miltefosine, fexinidazole and fexinidazole sulfone were added to each set of experiments. After a 48 h incubation period at 37 °C, each plate-well was then microscopically examined to detect any precipitate formation.

*Luciferase activity and determination of* EC_50_ *were performed as above.*

#### 3.3.4. Antileishmanial Activity on *L. infantum* Intracellular Amastigotes

The undifferentiated THP1 human monocyte cells (acute monocytic leukemia cell line purchased from ATCC, ref TIB-202) were grown in RPMI 1640 medium (Life Technologies, Saint-Aubin, France) supplemented with 10% FCS (Life Technologies, Saint-Aubin, France), 100 U/mL penicillin, 100 µg/mL streptomycin and 2 mM L-glutamine at 37 °C, 5% CO_2_. The culture was maintained between 3.10^5^ and 1.10^6^ cells/mL. The in vitro evaluation of the antileishmanial activity on intracellular amastigote forms of the tested compound was assessed as below.

Briefly, 200 μL of THP-1 cells with Phorbol 12-Myristate 13-Acetate (final concentration 50 ng/mL) were seeded in 96-well plates at an average density of 0.77 × 10^5^ cells/mL and incubated for 96 h at 37 °C, 5% CO_2_. Promastigotes were centrifuged at 3000 rpm for 10 min and the supernatant replaced by the same volume of complete RPMI pH 5.4 and incubated for 24 h at 27 °C. Differentiated THP-1 cells were then infected by acidified promastigotes with an infection ratio of twenty parasites for one macrophage and incubated for 24 h at 37 °C, 5% CO_2_. Plates were rinsed three times and then, in duplicate, medium containing various concentrations of reference drugs and DMSO was added (final DMSO concentrations being inferior to 0.5% *v*/*v*) and plates were incubated for 120 h.

*Luciferase activity and determination of* EC_50_ *were performed as above.*

#### 3.3.5. Antitrypanosomal Evaluation on *T. b. brucei* BSF Trypomastigotes

The effects of the tested compounds on the growth of *T. b. brucei* were assessed by Alamar Blue^®^ assay described by Räz et al. [30] *T. b. brucei* AnTat 1.9 (IMTA, Antwerpen, Belgium) was cultured in MEM with Earle’s salts, supplemented according to the protocol of Baltz et al. [31] with the following modifications: 0.5 mM mercaptoethanol (Sigma Aldrich^®^, France), 1.5 mM L-cysteine (Sigma Aldrich^®^), 0.05 mM bathocuproïne sulfate (Sigma Aldrich^®^) and 20% heat-inactivated horse serum (Gibco^®^, France), at 37 °C and 5% CO_2_. They were incubated at an average density of 2000 parasites/100 µL in sterile 96-wells plates (Fisher^®^, France) with various concentrations of compounds dissolved in DMSO, in duplicate. Appropriate controls suramin, treated with sterile water, and fexinidazole, treated with DMSO on sterile water (reference drugs purchased from Sigma Aldrich, France and Fluorochem, UK) were added to each set of experiments. After a 69 h incubation period at 37 °C, 10 µL of the viability marker Alamar Blue^®^ (Fisher, France) were then added to each well, and the plates were incubated for 5 h. The plates were read in a ENSPIRE microplate reader (PerkinElmer, Waltham, MA, USA) using an excitation wavelength of 530 nm and an emission wavelength of 590 nm. EC_50_ was defined as the concentration of drug necessary to inhibit by 50% the activity of *T. b. brucei* relative to the control. EC_50_ were calculated by nonlinear regression analysis processed on dose-response curves, using GraphPad Prism software (USA). EC_50_ values were calculated from three separate experiments.

#### 3.3.6. Cytotoxicity Evaluation on HepG2 Cell Line

HepG2 cell line (hepatocarcinoma cell line purchased from ATCC, ref HB-8065) was maintained at 37 °C, 5% CO_2_ with 90% humidity in MEM supplemented with 10% fœtal bovine serum, 1% L-glutamine (200 mM) and penicillin (100 U/mL)/streptomycin (100 mg/mL) (complete MEM medium). The tested molecules’ cytotoxicity was assessed according to the method of Mosmann [25] with slight modifications. Briefly, 5 × 10^3^ cells in 100 µL of complete medium were inoculated into each well of 96-well plates and incubated at 37 °C in a humidified 5% CO_2_. After 24 h incubation, 100 µL of medium with various product concentrations dissolved in DMSO (final concentration less than 0.5% *v*/*v*) were added and the plates were incubated for 72 h at 37 °C. Triplicate assays were performed for each sample. Each plate-well was then microscope-examined to detect possible precipitate formation before the medium was aspirated from the wells. 100 µL of MTT (3-(4,5-dimethyl-2-thiazolyl)-2,5-diphenyl-2*H*-tetrazolium bromide) solution (0.5 mg/mL in medium without FCS) were then added to each well. Cells were incubated for 2 h at 37 °C, following which the MTT solution was removed and DMSO (100 µL) was added to dissolve the resulting blue formazan crystals. Plates were shaken vigorously (700 rpm) for 10 min. The absorbance was measured at 570 nm with 630 nm as reference wavelength using a BIO-TEK ELx808 Absorbance Microplate Reader. DMSO was used as blank and doxorubicin (purchased from Sigma Aldrich) as positive control. Cell viability was calculated as percentage of control (cells incubated without compound). The 50% cytotoxic concentration (CC_50_) was determined from the dose–response curve by using the TableCurve 2D v5.0 software (Systat Software Inc. (Palo Alto, CA, USA)). CC_50_ values represent the mean value calculated from three separate experiments.

#### 3.3.7. Cytotoxicity on THP-1 Cell Line

Cells in 100 µL of complete RPMI medium, were incubated at an average density of 5 × 10^4^ cells/mL in sterile 96-well plates with various concentrations of compounds dissolved in DMSO (final concentration less than 0.5% *v*/*v*), and references in duplicate. The plates were incubated for 72 h at 37 °C. Each well plate was then microscope-examined for detecting possible precipitate formation before the medium was aspirated from the wells. 100 µL of MTT solution (0.5 mg/mL in medium) were then added to each well. Cells were incubated for 2 h at 37 °C. After this time, the MTT solution was removed and DMSO (100 µL) was added to dissolve the resulting blue formazan crystals. Plates were shaken vigorously (300 rpm) for 10 min. The absorbance was measured at 570 nm using a BIO-TEK ELx808 Absorbance Microplate Reader. DMSO was used as blank and doxorubicin (purchased from Sigma Aldrich) as positive control. Cell viability was calculated as percentage of control (cells incubated with DMSO). The 50% cytotoxic concentration (CC_50_) was determined from the dose–response curve by using the TableCurve 2D V5 software Systat Software Inc. (Palo Alto, CA, USA). CC_50_ values represent the mean value calculated from three separate experiments.

#### 3.3.8. Plasma Protein Binding

Plasma doped with the tested compound was incubated at 37 °C in triplicate in one of the compartments of the insert, the other compartment containing a phosphate buffer solution at pH 7.2. After stirring for 4 h at 300 rpm, a 25 μL aliquot of each compartment was taken and diluted; the dilution solution was adapted so as to obtain an identical matrix for all the compartments after dilution. Parallel, reprocessing of a plasma doped but not incubated was used to assess recovery. The LC-MS used for this study was a Waters^®^ Acquity I-Class/Xevo TQD, equipped with a Waters^®^ Acquity BEH C18 column, 50 × 2.1 mm, 1.7 µM. The mobile phases were (A) ammonium acetate 10 mM and (B) acetonitrile with 0.1% formic acid. The injection volume was 1 µL and the flow rate 600 µL/min. Chromatographic analysis, total duration of 4 min, was performed along the following gradient: 0 < t < 0.2 min, 2% (B); 0.2 < t < 2 min, linear increase to 98% (B); 2 < t < 2.5 min, 98% (B); 2.5 < t < 2.6 min, linear decrease to 2% (B); 2.6 < t < 4 min, 2% (B). Carbamazepine, oxazepam, warfarin and diclofenac were used as reference drugs and propranolol as internal standard. The unbound fraction (f_u_) was calculated according to the following formula: $f_{u}=\frac{A_{\mathrm{Plasma},\;4h}-A_{\mathrm{PBS},4h}}{A_{\mathrm{Plasma},\;4h}}\times 100$. The percentage of recovery was calculated according to the following formula:

$$\%\;\mathrm{Recovery}=\frac{\left(V_{\mathrm{PBS}}\times A_{\mathrm{PBS},4h}\right)+\left(V_{\mathrm{Plasma}}\times A_{\mathrm{Plasma},4h}\right)}{\left(V_{\mathrm{Plasma}}\times A_{\mathrm{Plasma},0h}\right)}$$

where A is the ratio of the area under peak of the studied molecule and the area under peak of the internal standard (propranolol 200 nM). V is the volume of solution present in the compartments (V_PBS_ = 350 µL and V_plasma_ = 200 µL).

#### 3.3.9. Microsomal Stability

The tested product and propranolol, used as reference, were incubated in duplicate (reaction volume of 0.5 mL) with female mouse microsomes (CD-1, 20 mg/mL, BD Gentest™) at 37 °C in a 50 mM phosphate buffer, pH 7.4, in the presence of MgCl_2_ (5 mM), NADP (1 mM), glucose-6-phosphate dehydrogenase (0.4 U/mL) and glucose-6-phosphate (5 mM). To access intrinsic clearance, 50 µL aliquot at 0, 5, 10, 20, 30 and 40 min were collected and the reaction was stopped with 4 volumes of acetonitrile (ACN) containing the internal standard. After centrifugation at 10,000× *g*, 10 min, 4 °C, the supernatants were maintained at 4 °C for immediate analysis or placed at -80 °C for later analysis. Controls (t_0_ and t_final_) in triplicate were prepared by incubation of the internal standard with microsomes denatured by acetonitrile. The LC-MS used for this study was a Waters^®^ Acquity I-Class/Xevo TQD, equipped with a Waters^®^ Acquity BEH C18 column, 50 × 2.1 mm, 1.7 µm. The mobile phases were (A) ammonium acetate 10 mM and (B) acetonitrile with 0.1% formic acid. The injection volume was 1 µL and the flow rate 600 µL/min. Chromatographic analysis, total duration of 4 min, was perform along the following gradient: 0 < t < 0.2 min, 2% (B); 0.2 < t < 2 min, linear increase to 98% (B); 2 < t < 2.5 min, 98% (B); 2.5 < t < 2.6 min, linear decrease to 2% (B); 2.6 < t < 4 min, 2% (B). 8-Bromo-6-chloro-3-nitro-2-(phenylsulfonylmethyl)imidazo[1,2-*a*]pyridine Hit **A** is used as internal standard. Each compound was quantified by converting the average of the ratios of the analyte/internal standard surfaces to the percentage of product consumed. The ratio of the control at t_0_ corresponded to 0% of product consumed. The half-life (T_1/2_) of each compound in the presence of microsomes was calculated according to the equation: $t_{\frac{1}{2}}=\frac{\ln\left(2\right)}{k}$, where k is the first-order degradation constant (the slope of the logarithm of compound concentration versus incubation time). The intrinsic clearance in vitro (Cl_int_ expressed in μL/min/mg) is calculated according to the equation:

Clint=doseAUC∞[microsomes]

where “dose” is the initial concentration of product in the sample, AUC_∞_ is the area under the concentration-time curve extrapolated to infinity and [microsomes] is the microsome concentration expressed in mg/µL.

#### 3.3.10. Parallel Artificial Membrane Permeability Assay (PAMPA)

The Pampa-BBB experiments were conducted using the Pampa Explorer Kit (Pion Inc) according to manufacturer’s protocol. Briefly, the stock compound solution (20 mM in DMSO) was diluted in Prisma HT buffer pH 7.4 (pION) to 100 µM. 200 µL of this solution (*n* = 6) were added to the donor plate (P/N 110243). 5 µL of the BBB-1 Lipid (P/N 110672) was used to coat the membrane filter of the acceptor plate (P/N 110243). 200 µL of the Brain Sink Buffer (P/N 110674) were added to each well of the acceptor plate. The sandwich was incubated at room temperature for 4 h, without stirring. After incubation the UV-visible spectra were measured with the microplate reader (Tecan infinite M200) and the permeability value (P_e_) was calculated using PAMPA Explorer software v.3.7 (pION). Corticosterone (P_e_ = 138.6 ± 22.0 nm/s), and theophylline (P_e_ = 5.5 ± 0.3 nm/s) were used as high and low permeability standards, respectively. Each measurement was performed in sextuplicate.

#### 3.3.11. Thermodynamic Solubility at pH 7.4

Thermodynamic solubility at pH 7.4 of compounds was determined according to a miniaturized shake-flask method (Organisation for Economic Cooperation and Development guideline n°105) [32]. Phosphate Buffer solutions (pH 7.4, 10 µM, ionic strength 150 µM) were prepared from Na_2_HPO_4_, KH_2_PO_4_ and KCl (Sigma Aldrich, Saint Quentin Fallavier, France); 10 µL of 20 mM stock solution were added to 5 mL glass tubes containing 990 mL buffer (*n* = 3). The tubes were briefly sonicated and shacked by inversion during 24 h at 23 °C. Then, the tube contents were placed in a microtube which was centrifuged at 12,225× *g* for 10 min; 100 µL supernatant were mixed with 100 µL acetonitrile in a Greiner UV microplate. Standard solutions were prepared extemporaneously, diluting 20 mM DMSO stock solutions at 0, 5, 10 and 20 mM; 5 µL of each working solution were diluted with 995 µL buffer and 100 µL were then mixed in microplates with 100 µL acetonitrile so as to ensure that the final proportions of each solvent in standard solutions and samples remained unchanged. Solubility at pH 7.4 was determined with an Infinite M200Pro (Tecan, Lyon, France) microplate reader in spectrophotometric mode (230 to 450 nm) from the specific λ_max_ of each compound. The calibration curve was obtained from the three standard solutions of tested compounds at 0, 25, 50, and 100 µM in a 50:50 (vol/vol) mixture of buffer with acetonitrinile/DMSO (99:1; vol/vol). Calibration curves were linear with R^2^ > 0.99.

## 4. Conclusions

This antileishmanial SAR study, which focused on positions 2 and 8 of the imidazo[1,2-*a*]pyridine ring, was conducted through the synthesis and assessment of 22 new derivatives. Of these, compound (**7**), bearing a *gem*-trifluoropropylsulfonylmethyl group at position 2 and a pyridin-4-yl moiety at position 8, displayed low cytotoxicities on both HepG2 and THP1 cell lines (CC_50_ > 100 µM) associated with a good activity against the intracellular amastigote stage of *L. infantum* (EC_50_ = 3.7 µM), making it a new antileishmanial hit compound. Moreover, (**7**) showed greatly improved aqueous solubility (thermodynamic solubility = 31.1 µM), good mouse microsomal stability (T_1/2_ > 40 min) and high gastrointestinal permeability in a PAMPA model, making it an ideal candidate for further in vivo studies on an infectious mouse model.

## Figures and Tables

**Table 1:** Structures and biological profiles of previously identified antikinetoplastid hit compounds in 3-nitroimidazo[1,2-*a*]pyridine series.

	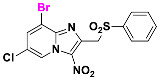	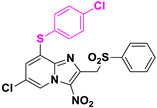
	Hit A	Hit B
EC_50_ *L. donovani* promastigotes (µM)	5.3 ± 1.1	1.0 ± 0.3
EC_50_ *L. infantum* promastigotes (µM)	8.7 ± 0.7	0.6 ± 0.1
EC_50_ *L. infantum* axenic amastigotes (µM)	16.4 ± 1.3	1.7 ± 0.3
EC_50_ *L. infantum* amastigotes intramacro. (µM)	2.1 ± 0.1	3.2 ± 0.1
CC_50_ HepG2 (µM)	>15.6	>100
CC_50_ THP1 (µM)	>100	>62.5
Microsomal stability: half-life (min)	11	3
Thermodynamic solubility at pH 7.4 (µM)	6.9	1.4

**Table 2:** In vitro evaluation of molecules **1c**-**7** on *L. donovani* promastigotes, *L. infantum* promastigotes, *L. infantum* axenic amastigotes, *L. infantum* intracellular amastigotes, the human hepatocyte HepG2 and human leukemia monocytic THP1 cell lines. Redox potentials (E_0_) of the nitro group were measured using cyclic voltammetry and corrected *versus* Normal Hydrogen Electrode (NHE). Weighted clogP were computed by the SwissADME online tool.

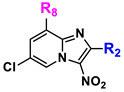			Cell Viability		Activity						
Compd	R_2_	R_8_	CC_50_ HepG2 (µM)	CC_50_ THP1 (µM)	EC_50_ *L. donovani* Pro. (µM)	EC_50_ *L. infantum* Pro. (µM)	EC_50_ *L. infantum* Axenic Ama. (µM)	EC_50_ *L. infantum* Intra. Ama. (µM)	SI HepG2/THP1 *L. infantum* Intra. Ama.	E_0_ (V)	ClogP
**2c**	-CH_2_-SO_2_-Ph-4-OH	-Br	>3.9 ^a^	>100 ^c^	>3.1 ^a^	>100 ^c^	>5.0 ^c^	-	-	-	2.4
**2d**	-CH_2_-SO_2_-Ph-4-Cl	-Br	>12.5 ^a^	>31.2 ^a^	3.0 ± 0.4	3.5 ± 0.4	2.1 ± 0.3	-	-	−0.62	3.28
**1c**	-CH_2_-SO_2_-Ph-4-F	-Cl	>7.8 ^a^	22.5 ± 1.1	5.8 ± 1.9	>100 ^c^	>1.6 ^a^	-	-	−0.6	2.98
**2e**	-CH_2_-SO_2_-Ph-4-F	-Br	>7.8 ^a^	33.3 ± 1.4	2.8 ± 0.5	>100 ^c^	3.3 ± 0.1	-	-	−0.64	3.06
**1d**	-CH_2_-SO_2_-Ph-4-CF_3_	-Cl	>15.6 ^a^	23.2 ± 1.9	4.1 ± 0.4	>100 ^c^	2.2 ± 0.1	-	-	−0.6	3.72
**2f**	-CH_2_-SO_2_-Ph-4-OCF_3_	-Br	>25 ^a^	>100 ^c^	4.4 ± 0.1	6.7 ± 0.8	2.7 ± 0.4	4.2 ± 0.3	>6.0/>23.8	−0.62	3.59
**2g**	-CH_2_-SO_2_-Ph-4-OCH_3_	-Br	3.9 ± 1.3	>100 ^c^	2.8 ± 0.9	9.3 ± 1.2	2.3 ± 0.3	2.2 ± 0.1	1.8/>45.5	−0.64	2.75
**2h**	-CH_2_-SO_2_-*i*Pr	-Br	>62.5	>100 ^c^	>25 ^c^	61.2 ± 3.4	>50 ^c^	-	-	−0.65	2.15
**2i**	-CH_2_-SO_2_-Cyclopropyl	-Br	>6.2 ^a^	>50	>25 ^c^	>50 ^c^	>50 ^c^	-	-	−0.63	1.97
**2j**	-CH_2_-SO_2_-*gem*-Trifluoropropyl	-Br	42.9 ± 12.3	26.0 ± 1.2	14.4 ± 1.6	1.9 ± 0.4	20.1 ± 5.6	-	-	−0.6	2.91
**3a**	-CH_2_-SO_2_-Ph-4-OH	4-Cl-Ph-S-	22.3 ± 2.5	28.2 ± 0.1	0.8 ± 0.2	5.6 ± 0.9	1.4 ± 0.3	2.7 ± 0.5	8.3/10.4	-	3.96
**3b**	-CH_2_-SO_2_-Ph-4-Cl	4-Cl-Ph-S-	8.9 ± 1.3	>15.6 ^a^	1.4 ± 0.1	1.2 ± 0.1	1.0 ± 0.1	0.7 ± 0.1	12.7/>22.3	−0.68	4.83
**3c**	-CH_2_-SO_2_-Ph-4-F	4-Cl-Ph-S-	>15.6 ^a^	>100 ^c^	0.9 ± 0.1	1.5 ± 0.1	0.2 ± 0.1	1.6 ± 0.1	>9.8/>62.5	−0.61	4.67
**3d**	-CH_2_-SO_2_-Ph-4-CF_3_	4-Cl-Ph-S-	>3.9 ^a^	>50	1.8 ± 0.2	3.3 ± 0.4	0.8 ± 0.1	4.4 ± 0.2	>0.9/>11.4	−0.56	5.28
**3e**	-CH_2_-SO_2_-Ph-4-OCF_3_	4-Cl-Ph-S-	>12.5 ^a^	>100 ^c^	2.6 ± 0.2	3.1 ± 0.2	0.8 ± 0.2	1.6 ± 0.1	>7.8/>62.5	−0.66	5.21
**3f**	-CH_2_-SO_2_-Ph-4-OCH_3_	4-Cl-Ph-S-	>3.9 ^a^	>50	1.2 ± 0.1	1.2 ± 0.1	0.5 ± 0.1	0.9 ± 0.1	>4.3/>55.6	−0.66	4.38
**3g**	-CH_2_-SO_2_-*i*Pr	4-Cl-Ph-S-	22.2 ± 5.5	>31.2 ^a^	2.2 ± 0.3	2.4 ± 0.6	3.2 ± 0.1	-	-	−0.63	3.73
**3h**	-CH_2_-SO_2_-Cyclopropyl	4-Cl-Ph-S-	22.3 ± 2.5	>62.5	2.2 ± 0.3	2.2 ± 0.3	2.4 ± 0.1	3.7 ± 0.2	6.0/>16.9	−0.65	3.63
**3i**	-CH_2_-SO_2_-*gem*-Trifluoropropyl	4-Cl-Ph-S-	>6.2 ^a^	>50	1.4 ± 0.2	1.4 ± 0.1	0.8 ± 0.3	1.2 ± 0.1	>5.2/>41.7	−0.64	4.4
**6a**	-CH_2_-SO_2_-*gem*-Trifluoropropyl	4-Cl-Ph-SO-	>5	>100	1.9 ± 0.3	7.4 ± 0.4	5.0 ± 0.1	3.3 ± 0.2	>1.5/>30.3	-	3.79
**6b**	-CH_2_-SO_2_-*gem*-Trifluoropropyl	4-Cl-Ph-SO2-	>10	>100	1.3 ± 0.2	13.7 ± 0.9	2.3 ± 0.3	8.3 ± 0.8	>1.2/>12.0	-	3.63
**7**	**-CH_2_-SO_2_-*gem*-Trifluoropropyl**	**4-pyridinyl**	**>100**	**>100**	**3.1 ± 0.6**	**14.0 ± 0.4**	**14.0 ± 0.9**	**3.7 ± 0.4**	**>27.0/>27.0**	**−0.61**	**2.93**
Ref. [1]	Hit A		>15.6 ^a^	>100 ^c^	5.3 ± 1.1	8.7 ± 0.7	16.4 ± 1.3	2.1 ± 0.1	>7.4/>47.6	−0.65	2.81
Ref. [2]	Hit B		>100	>62.5	1.0 ± 0.3	0.6 ± 0.1	1.7 ± 0.3	3.2 ± 0.1	>31.3/>19.5	−0.63	4.37
Ref. [3]	Doxorubicin ^b^		0.20 ± 0.02	0.8 ± 0.1	-	-	-	-	-	-	0.52
Ref. [4]	Amphotericin B ^d^		8.8 ± 0.3	12.1 ± 1.3	0.07 ± 0.01	0.04 ± 0.01	0.08 ± 0.02	0.06 ± 0.01	146.7/201.7	-	−0.26
Ref. [5]	Miltefosine ^d^		85.0 ± 8.8	28.1 ± 3.4	3.1 ± 0.2	18.2 ± 0.5	0.30 ± 0.02	0.40 ± 0.14	212.5/70.3	-	3.83
Ref. [6]	Fexinidazole ^d^		>100	>100	1.2 ± 0.2	1.0 ± 0.1	7.8 ± 2.3	15.9 ± 3.2	>6.3/>6.3	−0.83	1.61
Ref. [7]	Fexinidazole sulfone ^d^		-	>100	1.2 ± 0.2	1.1 ± 0.1	4.3 ± 0.1	17.8 ± 1.4	-/>5.6	-	0.66

^a^ The product could not be tested at higher concentrations due to a lack solubility in the culture medium. ^b^ Doxorubicin was used as a cytotoxic reference drug. ^c^ The EC_50_ or CC_50_ value was not reached at the highest tested concentration. ^d^ Amphotericin B, Miltefosine, Fexinidazole and Fexinidazole sulfone were used as antileishmanial reference drugs.

**Table 3:** In vitro evaluation of molecules **2c**, **3a**, **3c** and **3d** on *T. b. brucei* trypomastigotes (BSF).

	CC_50_ HepG2 (µM)	EC_50_ *T. b. brucei* BSF (µM)	SI *T. b. brucei* ^c^
**2c**	>3.9 ^a^	0.40 ± 0.03	>9.8
**3a**	22.3 ± 2.5	0.78 ± 0.05	28.6
**3c**	>15.6 ^a^	0.57 ± 0.02	>27.4
**3d**	>3.9 ^a^	0.89 ± 0.07	>4.4
**Hit A**	>15.6 ^a^	1.4 ± 0.4	>11.1
**Hit B**	>100	0.95 ± 0.05	>105.3
**Suramin** ^b^	>100	0.08 ± 0.04	>1250
**Fexinidazole** ^b^	>200	1.4 ± 0.5	>142.9

^a^ The product could not be tested at higher concentrations due to a lack solubility in the culture medium. ^b^ Suramin and Fexinidazole were used as anti-*Trypanosomoa brucei* reference drugs. ^c^ SI = CC_50_ HepG2/EC_50_ *T. brucei brucei*.

**Table 4:** In vitro physicochemical and pharmacokinetic properties of selected key compounds.

	Thermodynamic Solubility at pH 7.4 in PBS (µM)	Mouse Microsomal Stability T_1/2_ (min)	PAMPA GIT at pH 7.4 P_e_ (nm/s)	Albumin ^a^ Binding (%)
**2f**	8.6 ± 2.3	-	-	-
**2g**	5.7 ± 1.3	-	-	-
**3b**	<2	4.5	-	-
**3c**	<2	5.7	-	-
**3d**	2.5 ± 0.7	-	-	-
**3e**	**64.7 ± 7.3**	-	-	-
**3f**	<2	-	-	-
**3h**	3.0 ± 0.4	-	-	-
**3i**	12.4 ± 3.0	9.1	6.3 ± 0.8	99.76
**6b**	-	7.9	-	-
**7**	**31.1 ± 3.7**	**>40**	**314.0 ± 87.2**	**88.65**
**Hit A**	6.9 ± 1.5	11.0	173.2 ± 38.8	96.50
**Hit B**	1.4 ± 0.5	3.0	4.0 ± 0.6	99.95

^a^ Albumin from human plasma.

**Table 5:** Validation of key antileishmanial hit selection criteria by compound **7**.

Key Hit Selection Criteria	Compound 7
Demonstrate an EC_50_ < 10 µM against the intracellular amastigote form of *L. donovani* or *L. infantum*	EC_50_ *L. infantum* intra. ama. = 3.7 µM
Chemical structure should be confirmed by identification, re-synthesis or re-purification	✓
Primary screening data should be validated on a selection of hit compounds (>90% pure)	Purity > 90% for Hit **A**, Hit **B** and all synthesized compounds (checked by LC/MS analyses)
Acceptable in vitro response	✓
Preliminary knowledge of the structure-activity relationship is desirable	✓
No highly reactive or unstable moieties in the pharmacophore, be amenable to structural variation by chemical or biochemical synthesis, pass basic drug-like filters such as pan-assay interference filters (PAINS), to eliminate promiscuous hits that lack target specificity, and the conformity to the “rule of five” is preferred	PAINS = 0 ^a^ Rule of five = 0 violation ^a^
Demonstrate a greater than 10-fold selectivity window for cytotoxicity using a mammalian cell line	SI HepG2 > 27 SI THP1 > 27
Demonstrate adequate selectivity in a biochemical counter-assay	Only relevant for target-based screens, not applicable in this case
No serious intellectual property conflicts should exist	✓
No major synthesis or formulation issues should be anticipated (compounds should ideally be synthesized in ≤5 steps with an acceptable yield and acceptable solubility)	Synthesized in 4 steps

^a^ Computed by the SwissADME online tool.

## Data Availability

Data is contained within the article and supplementary material.

## Supplementary Materials

The following supporting information can be downloaded at: https://www.mdpi.com/article/10.3390/ph15080998/s1. ^1^H and ^13^C NMR spectra.
