# Supplementary material for: Improving Aqueous Solubility and In Vitro Pharmacokinetic Properties of the 3-Nitroimidazo[1,2-a]pyridine Antileishmanial Pharmacophore
Source: Pharmaceuticals (Basel). 2022 Aug 13;15(8):998. doi: 10.3390/ph15080998 (PMC9415646; doi:10.3390/ph15080998)
Supplement: Supplementary file 1 [file pharmaceuticals-15-00998-s001.zip › pharmaceuticals-1848870-supplementary.pdf]

Improving aqueous solubility and in vitro pharmacokinetic properties of the 3-nitroimidazo[1,2-*a*]pyridine antileishmanial pharmacophore

Romain Paoli-Lombardo [1], Nicolas Primas [1,2,*], Sandra Bourgeade-Delmas [3,*], Sébastien Hutter [4], Alix Sournia-Saquet [5], Clotilde Boudot [6], Emilie Brenot [6], Caroline Castera-Ducros [1], Sophie Corvaisier [7], Marc Since [7], Aurélie Malzert-Fréon [7], Bertrand Courtioux [6], Alexis Valentin [3], Pierre Verhaeghe [5,8], Nadine Azas [4], Pascal Rathelot [1,2] and Patrice Vanelle [1,2,*]

[1] Aix Marseille Univ, CNRS, ICR UMR 7273, Equipe Pharmaco-Chimie Radicalaire, Faculté de Pharmacie, 27 Boulevard Jean Moulin, CS30064, 13385, Marseille Cedex 05, France

[2] AP-HM, Service Central de la Qualité et de l'Information Pharmaceutiques, Hôpital de la Conception, 13005, Marseille, France

[3] UMR 152 PHARMA-DEV, Université de Toulouse, IRD, UPS, Toulouse, France

[4] Aix Marseille Univ, IHU Méditerranée Infection, UMR VITROME - Tropical Eukaryotic Pathogens, 19-21 Boulevard Jean Moulin, 13005, Marseille, France

[5] LCC-CNRS Université de Toulouse, CNRS, UPS, 31077, Toulouse, France

[6] Université de Limoges, UMR Inserm 1094, Neuroépidémiologie Tropicale, Faculté de Pharmacie, 2 Rue Du Dr Marcland, 87025, Limoges, France

[7] Normandie Univ, UNICAEN, CERMN, 14000, Caen, France

[8] CHU de Nîmes, Service de Pharmacie, 30029, Nîmes, France

* Correspondence: nicolas.primas@univ-amu.fr (N.P.), patrice.vanelle@univ-amu.fr (P.V.), sandra.bourgeade-delmas@ird.fr (S.B.-D)

Supplementary materials

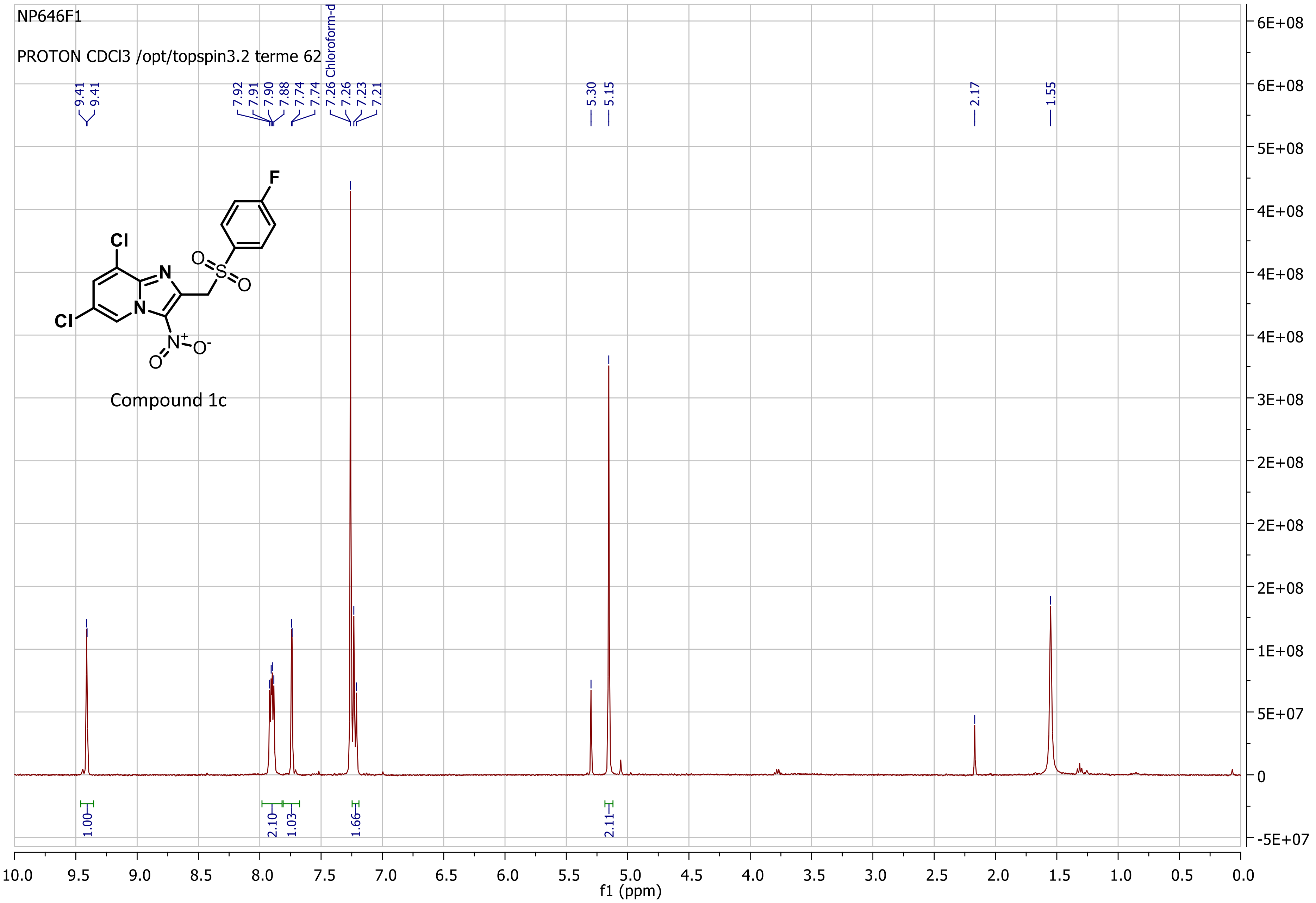
NP646F1
PROTON CDCl3 /opt/topspin3.2 terme 62
9.41
9.41
7.92
7.91
7.90
7.88
7.74
7.74
7.26 Chloroform-d
7.26
7.23
7.21
5.30
5.15
2.17
1.55
Cl
Cl
N
N
S
O
O
F
N+
O-
O
Compound 1c
1.00
2.10
1.03
1.66
2.11
f1 (ppm)

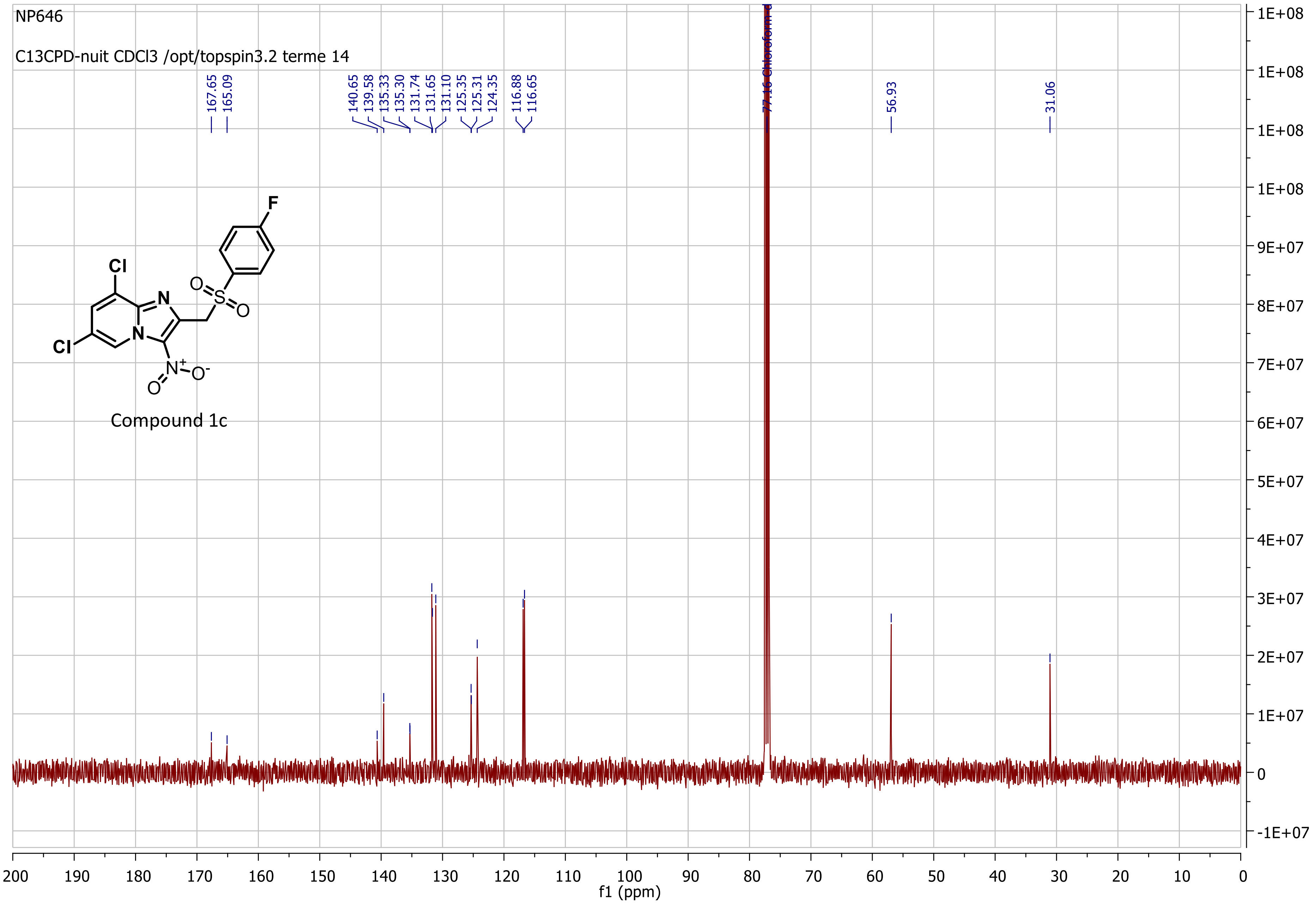
NP646
C13CPD-nuit CDCl3 /opt/topspin3.2 terme 14
167.65
165.09
140.65
139.58
135.33
135.30
131.74
131.65
131.10
125.35
125.31
124.35
116.88
116.65
77.16 Chloroform
56.93
31.06
F
Cl
O
S
O
N
N
Cl
N
O
O
Compound 1c
1E+08
9E+07
8E+07
7E+07
6E+07
5E+07
4E+07
3E+07
2E+07
1E+07
0
-1E+07
200 190 180 170 160 150 140 130 120 110 100 90 80 70 60 50 40 30 20 10 0
f1 (ppm)

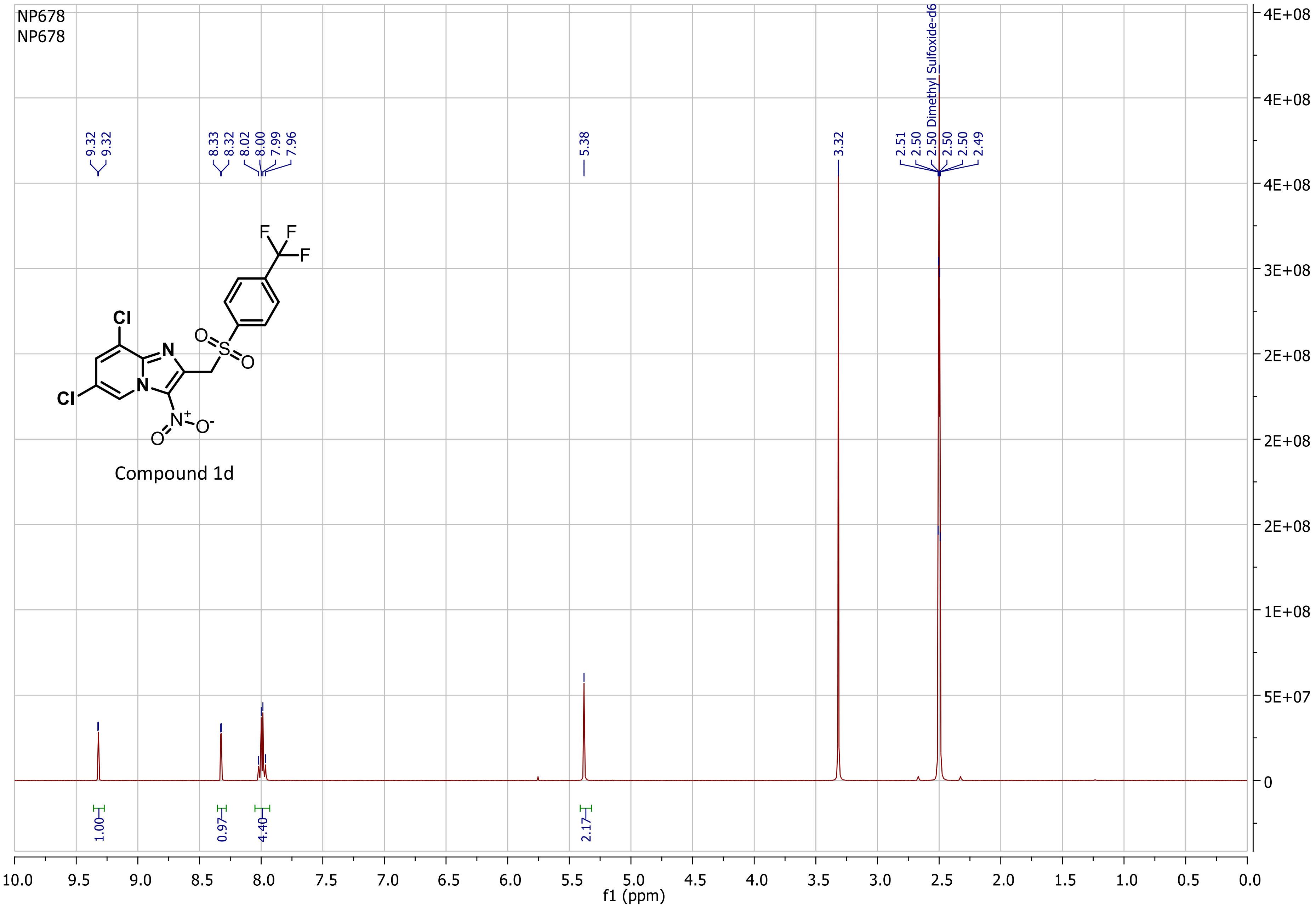
NP678
NP678
9.32
9.32
8.33
8.32
8.02
8.00
7.99
7.96
5.38
3.32
2.51
2.50
2.50 Dimethyl Sulfoxide-d6
2.50
2.50
2.49
Cl
Cl
N
N
O
S
O
F
F
F
N+
O
O-
Compound 1d
1.00
0.97
4.40
2.17
4E+08
4E+08
4E+08
3E+08
2E+08
2E+08
2E+08
1E+08
5E+07
0
10.0
9.5
9.0
8.5
8.0
7.5
7.0
6.5
6.0
5.5
5.0
4.5
4.0
3.5
3.0
2.5
2.0
1.5
1.0
0.5
0.0
f1 (ppm)

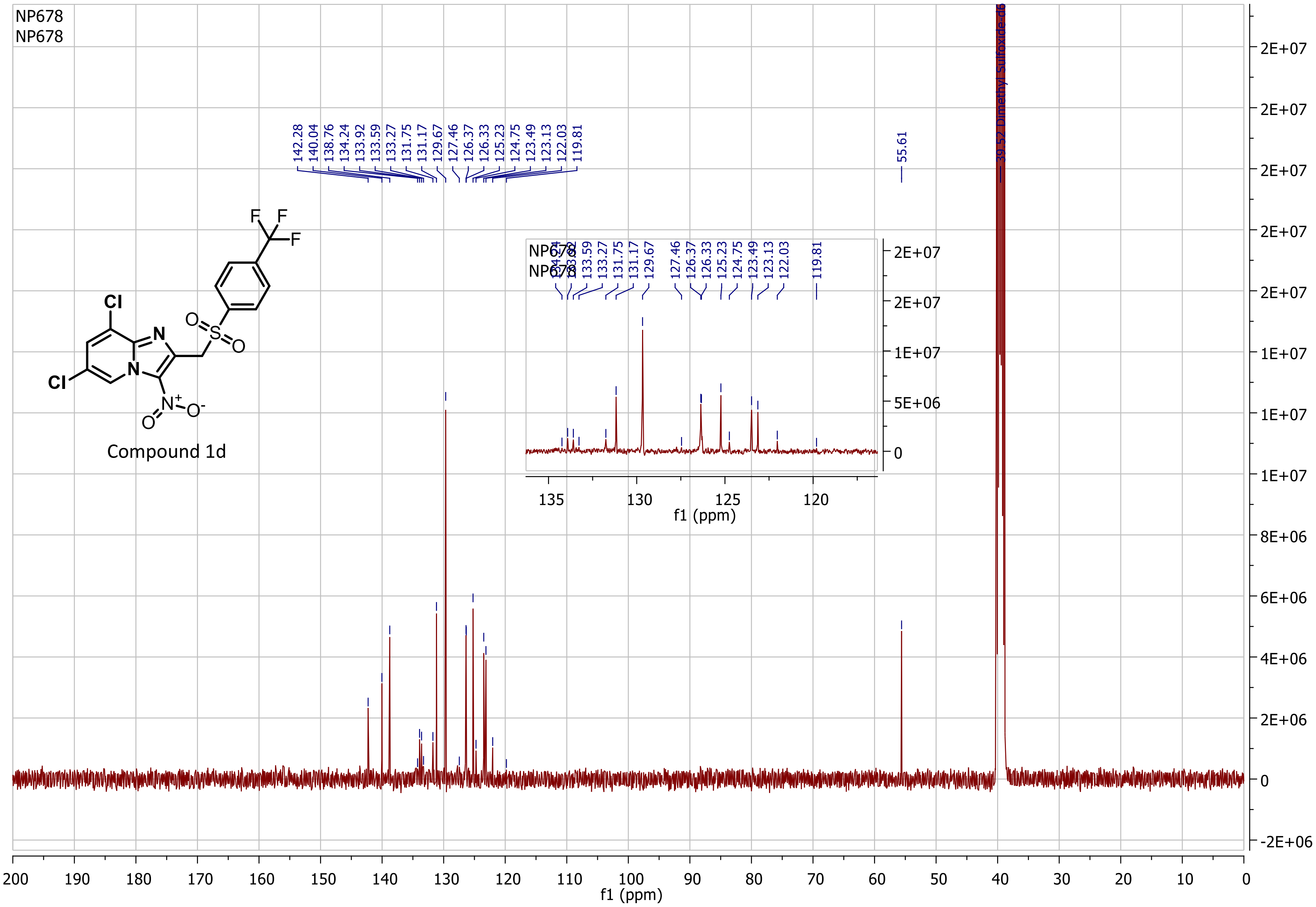
NP678
NP678
142.28
140.04
138.76
134.24
133.92
133.59
133.27
131.75
131.17
129.67
127.46
126.37
126.33
125.23
124.75
123.49
123.13
122.03
119.81
55.61
39.52 Dimethyl Sulfoxide-d6
Compound 1d
f1 (ppm)

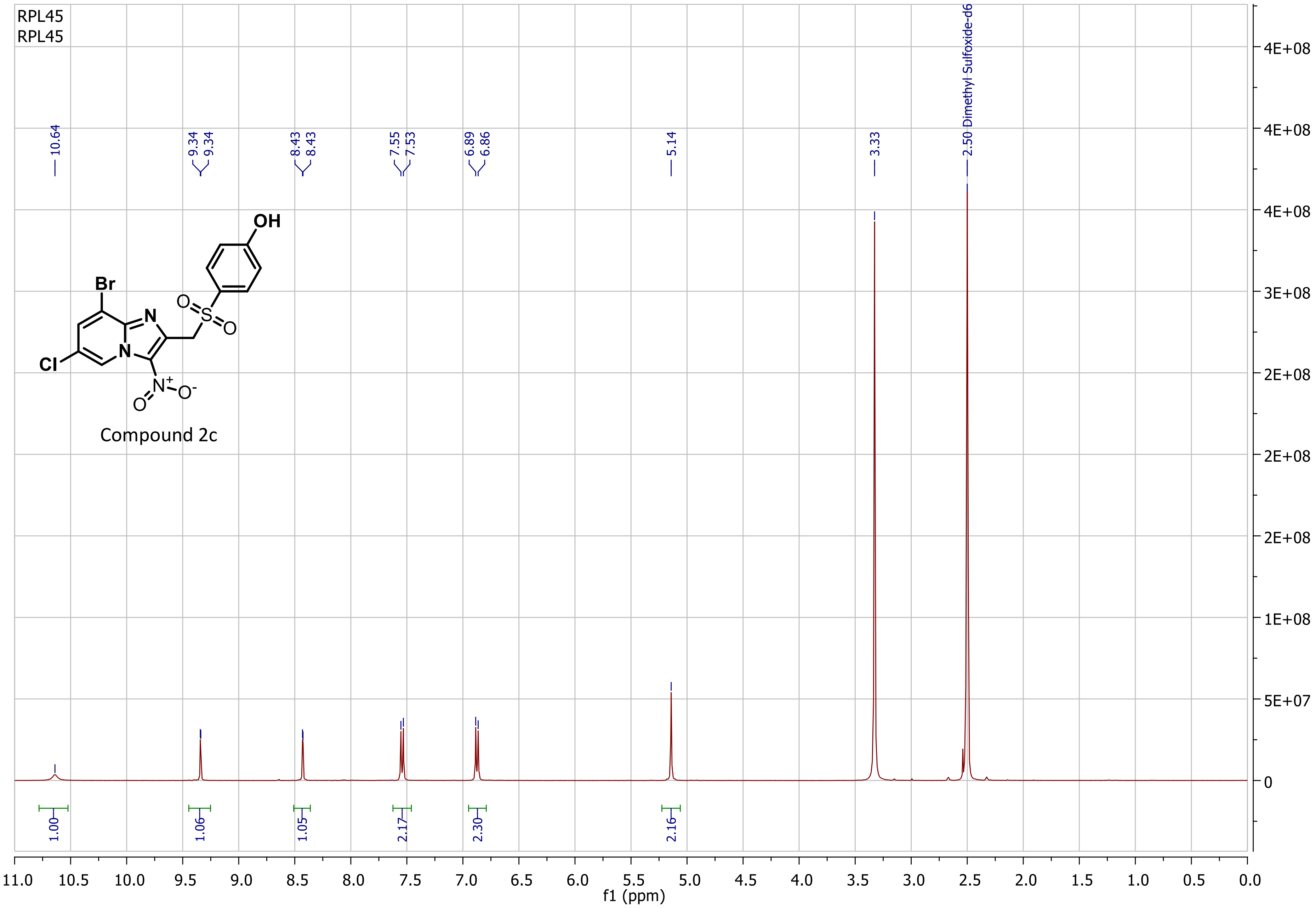
RPL45
RPL45
10.64
9.34
9.34
8.43
8.43
7.55
7.53
6.89
6.86
5.14
3.33
2.50 Dimethyl Sulfoxide-d6
OH
Br
O
S
O
N
N
Cl
N+
O-
O
Compound 2c
1.00
1.06
1.05
2.17
2.30
2.16
4E+08
4E+08
4E+08
3E+08
2E+08
2E+08
2E+08
1E+08
5E+07
0
11.0
10.5
10.0
9.5
9.0
8.5
8.0
7.5
7.0
6.5
6.0
5.5
5.0
4.5
4.0
3.5
3.0
2.5
2.0
1.5
1.0
0.5
0.0
f1 (ppm)

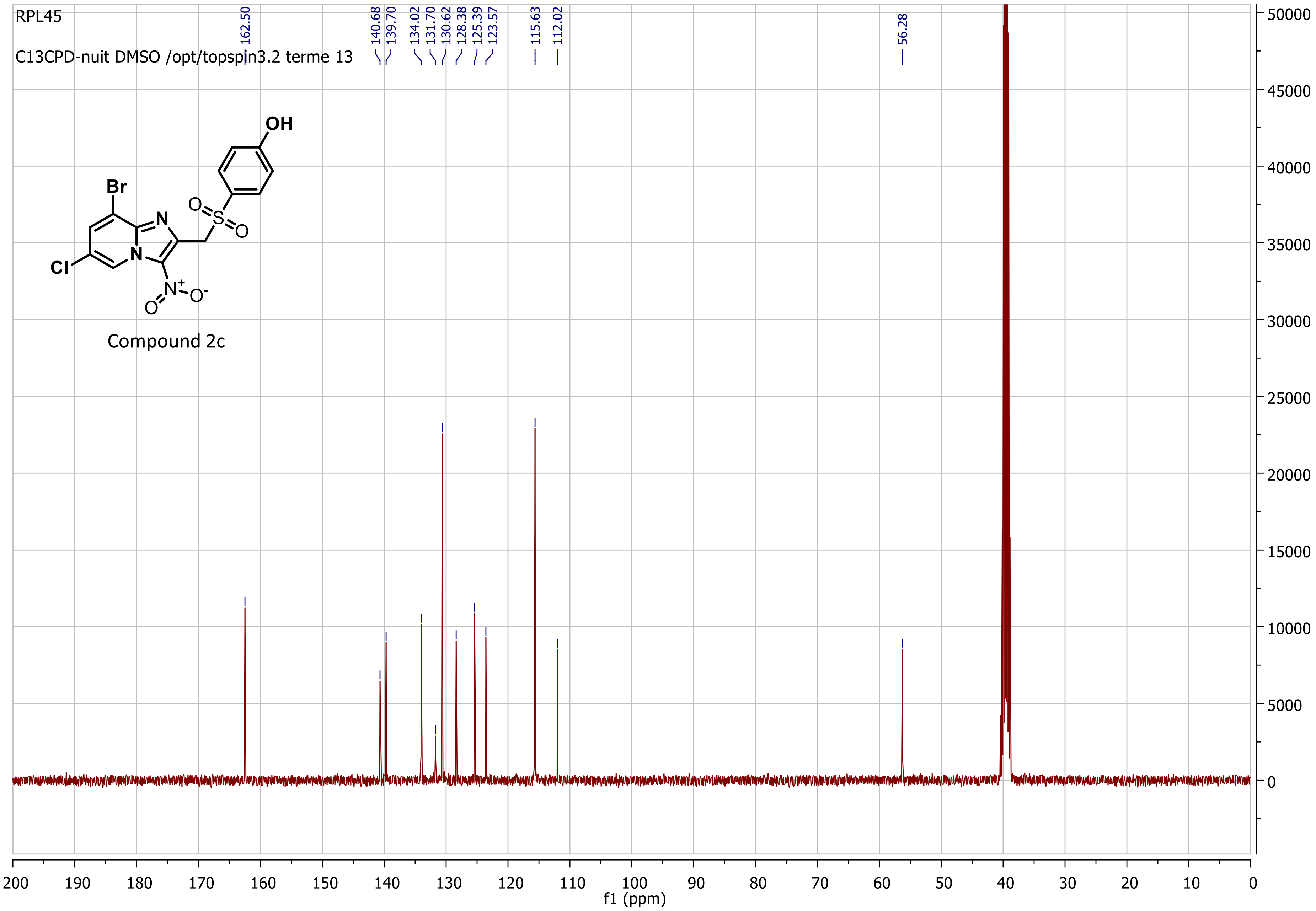
RPL45
C13CPD-nuit DMSO /opt/topspin3.2 terme 13
162.50
140.68
139.70
134.02
131.70
130.62
128.38
125.39
123.57
115.63
112.02
56.28
OH
Br
N
O
S
O
Cl
N
N+
O-
O
Compound 2c
f1 (ppm)
200 190 180 170 160 150 140 130 120 110 100 90 80 70 60 50 40 30 20 10 0
50000 45000 40000 35000 30000 25000 20000 15000 10000 5000 0

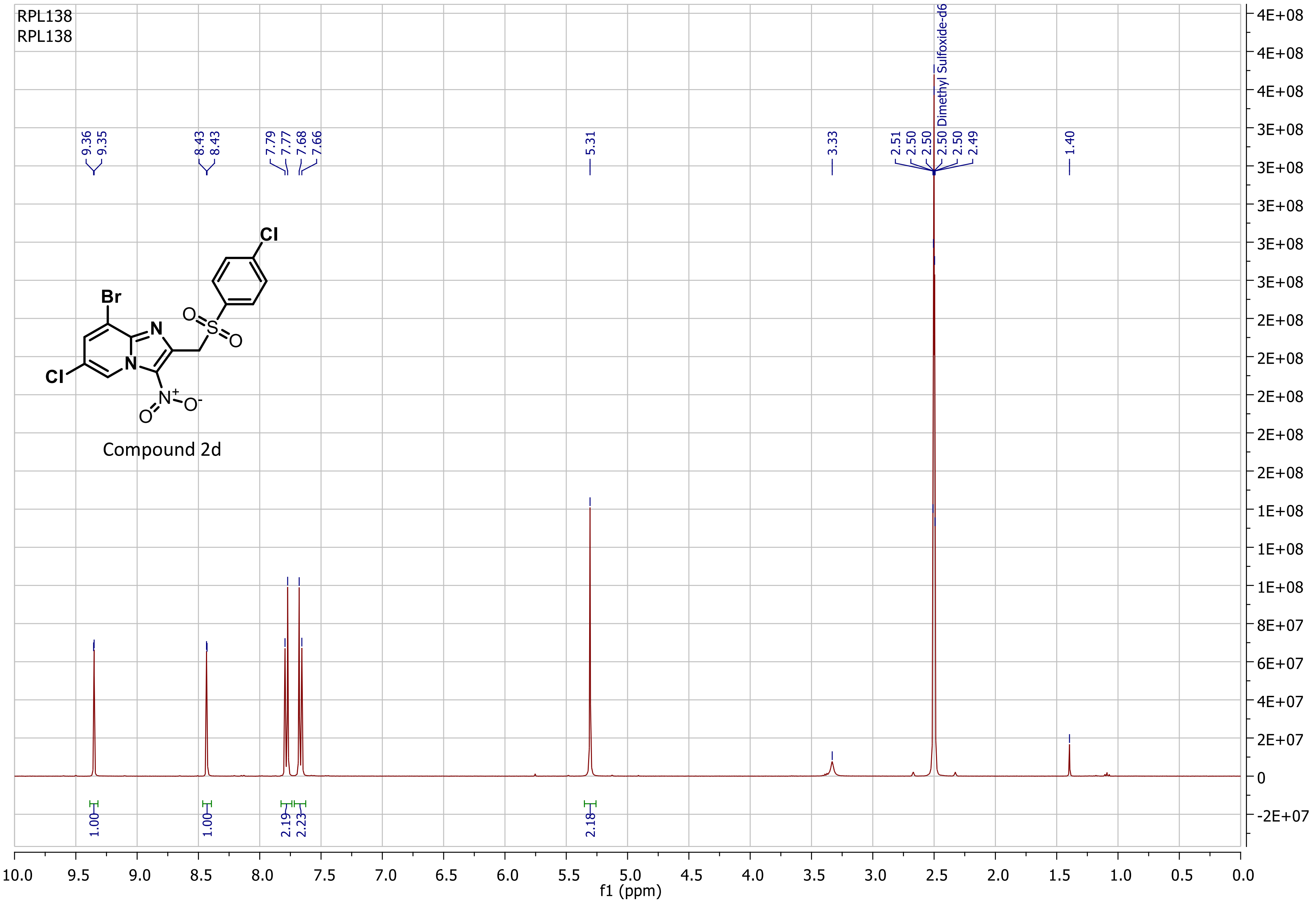
RPL138
RPL138
9.36
9.35
8.43
8.43
7.79
7.77
7.68
7.66
5.31
3.33
2.51
2.50
2.50
2.50 Dimethyl Sulfoxide-d6
2.50
2.49
1.40
Br
Cl
Cl
N
N
O
S
O
N+
O
O-
Compound 2d
1.00
1.00
2.19
2.23
2.18
f1 (ppm)

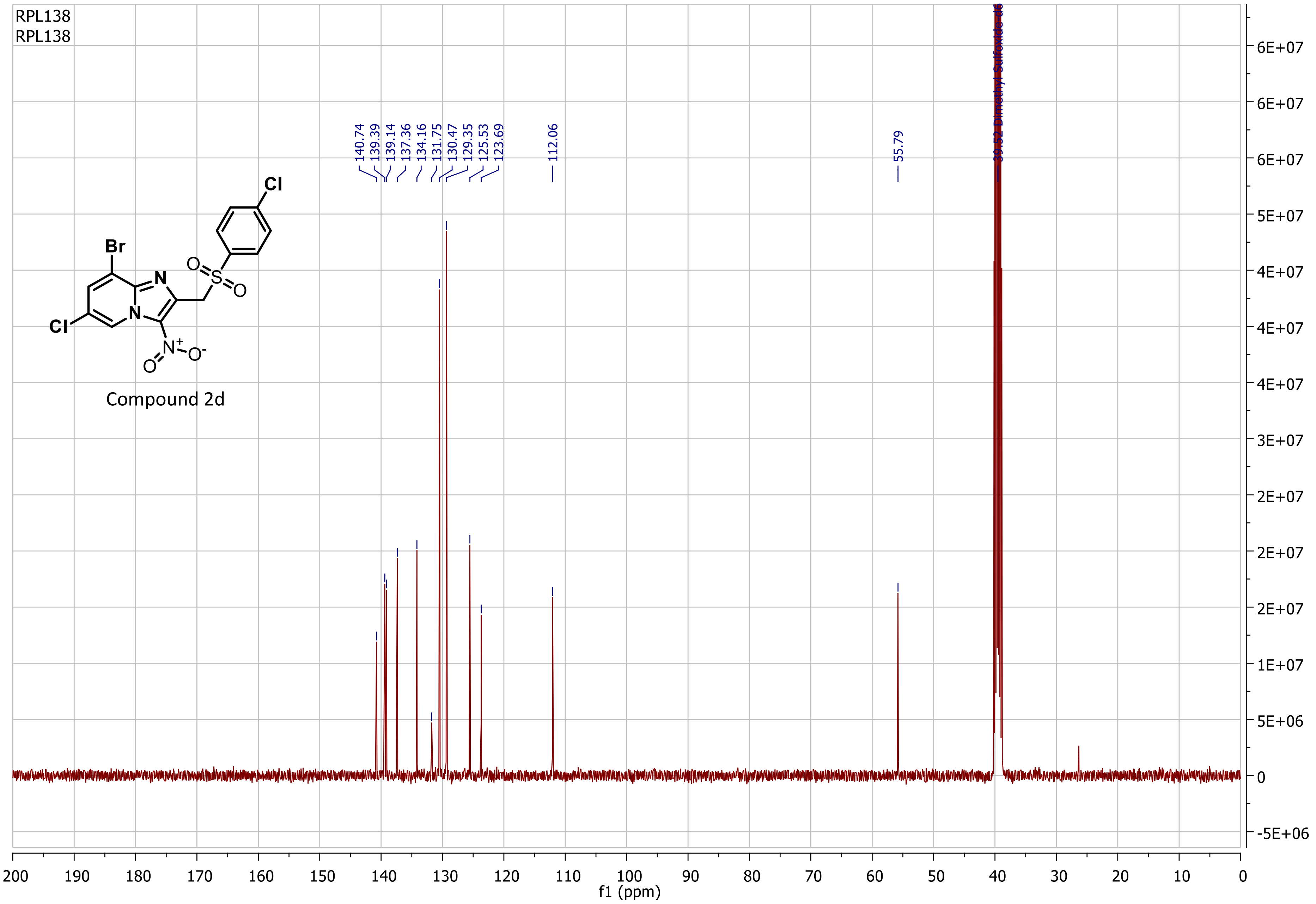

RPL138
RPL138
140.74
139.39
139.14
137.36
134.16
131.75
130.47
129.35
125.53
123.69
112.06
55.79
39.52 Dimethyl Sulfoxide-d6
Br
Cl
N
N
O
S
O
Cl
N+
O-
O
Compound 2d
6E+07
6E+07
6E+07
5E+07
4E+07
4E+07
4E+07
3E+07
2E+07
2E+07
2E+07
1E+07
5E+06
0
-5E+06
200
190
180
170
160
150
140
130
120
110
100
90
80
70
60
50
40
30
20
10
0
f1 (ppm)

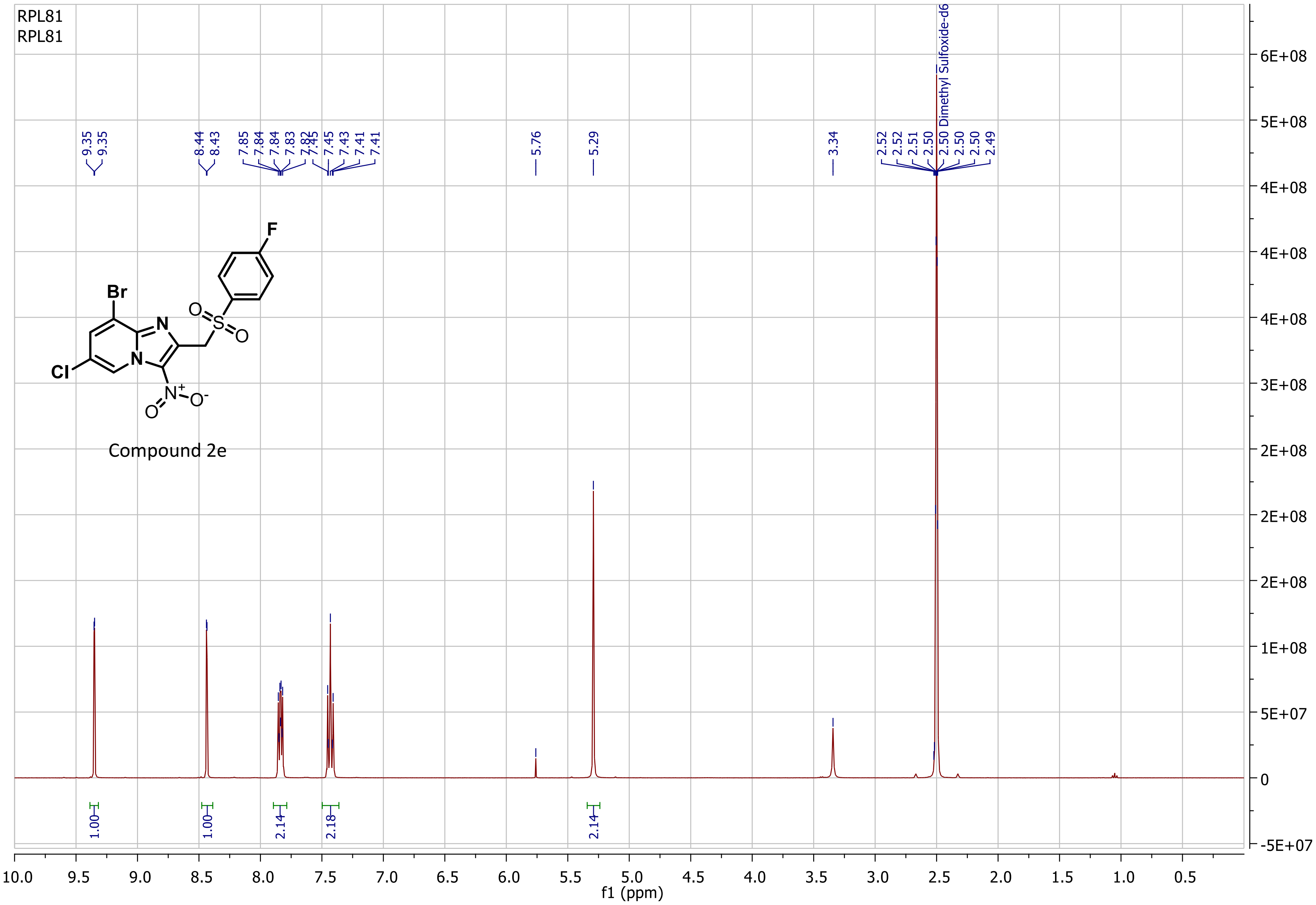
RPL81
RPL81
9.35
9.35
8.44
8.43
7.85
7.84
7.84
7.83
7.82
7.45
7.45
7.43
7.41
7.41
5.76
5.29
3.34
2.52
2.52
2.51
2.50
2.50 Dimethyl Sulfoxide-d6
2.50
2.50
2.49
Br
N
N
F
O
S
O
Cl
N+
O-
O
Compound 2e
1.00
1.00
2.14
2.18
2.14
6E+08
5E+08
4E+08
4E+08
4E+08
3E+08
2E+08
2E+08
2E+08
1E+08
5E+07
0
-5E+07
10.0
9.5
9.0
8.5
8.0
7.5
7.0
6.5
6.0
5.5
5.0
4.5
4.0
3.5
3.0
2.5
2.0
1.5
1.0
0.5
f1 (ppm)

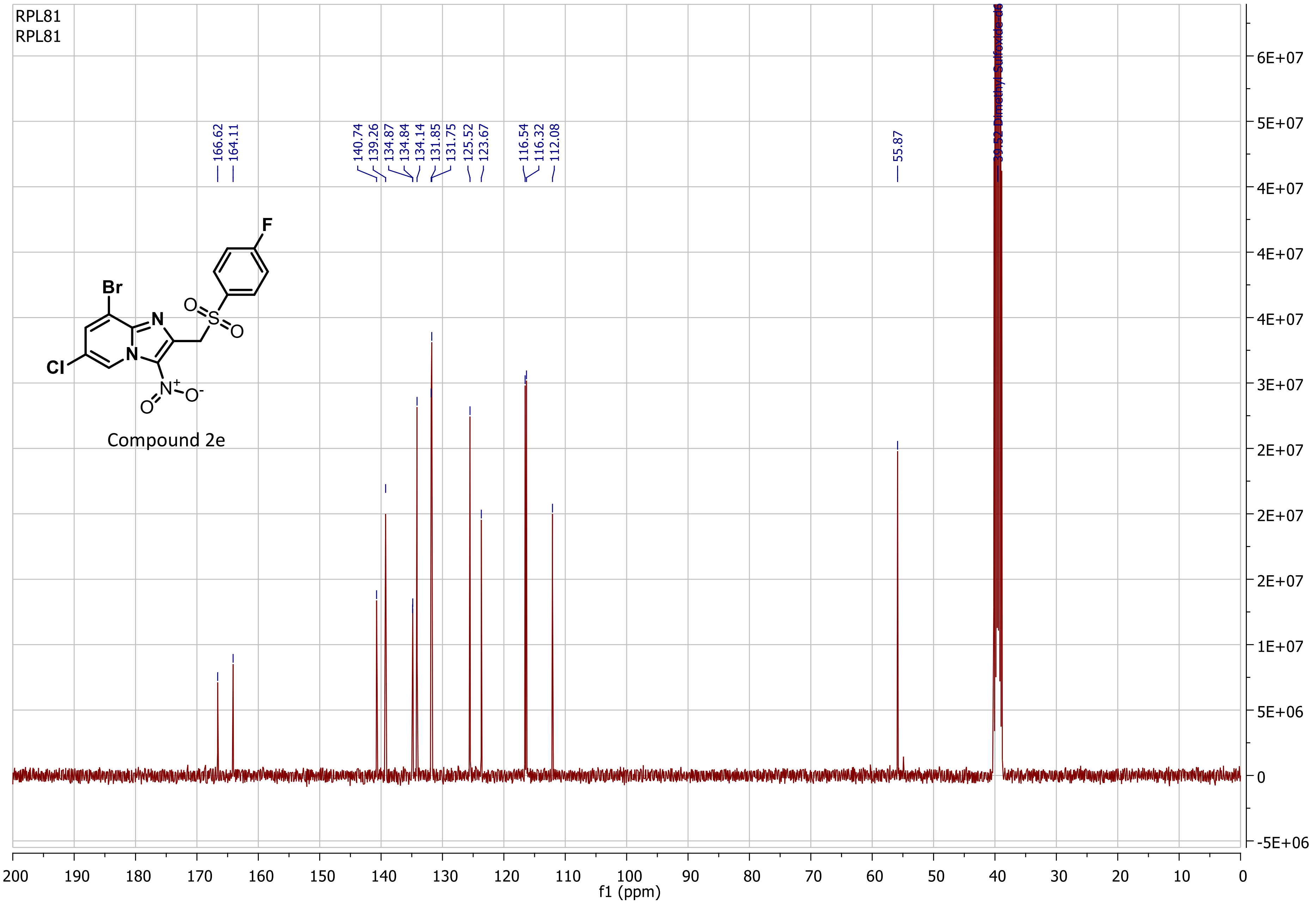
RPL81
RPL81
166.62
164.11
140.74
139.26
134.87
134.84
134.14
131.85
131.75
125.52
123.67
116.54
116.32
112.08
55.87
39.52 Dimethyl Sulfoxide-d6
Compound 2e
f1 (ppm)

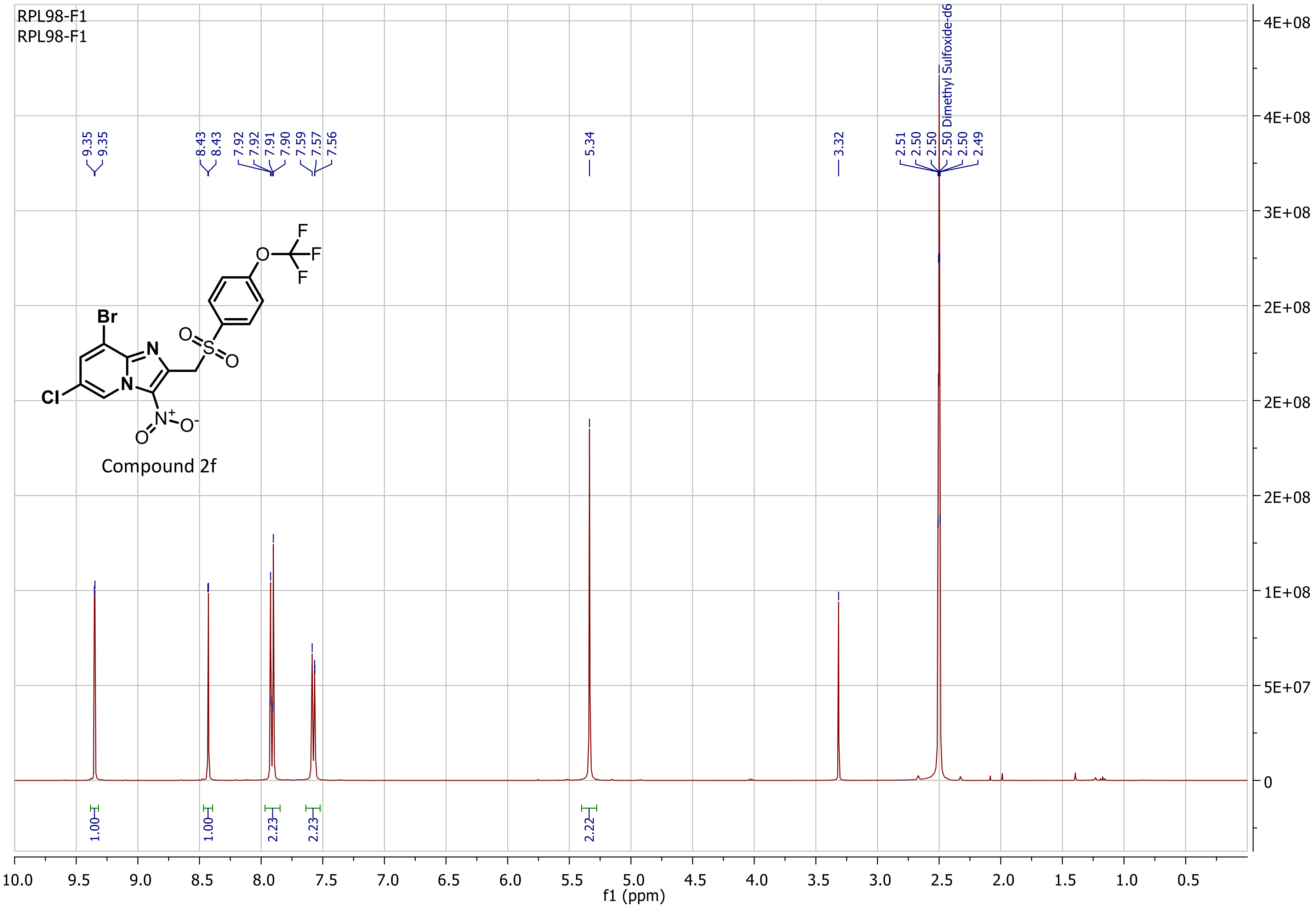
RPL98-F1
RPL98-F1
9.35
9.35
8.43
8.43
7.92
7.92
7.91
7.90
7.59
7.57
7.56
5.34
3.32
2.51
2.50
2.50
2.50 Dimethyl Sulfoxide-d6
2.50
2.49
Br
N
N
Cl
O
S
O
O
F
F
F
N+
O-
O
Compound 2f
1.00
1.00
2.23
2.23
2.22
4E+08
4E+08
3E+08
2E+08
2E+08
2E+08
1E+08
5E+07
0
10.0
9.5
9.0
8.5
8.0
7.5
7.0
6.5
6.0
5.5
5.0
4.5
4.0
3.5
3.0
2.5
2.0
1.5
1.0
0.5
f1 (ppm)

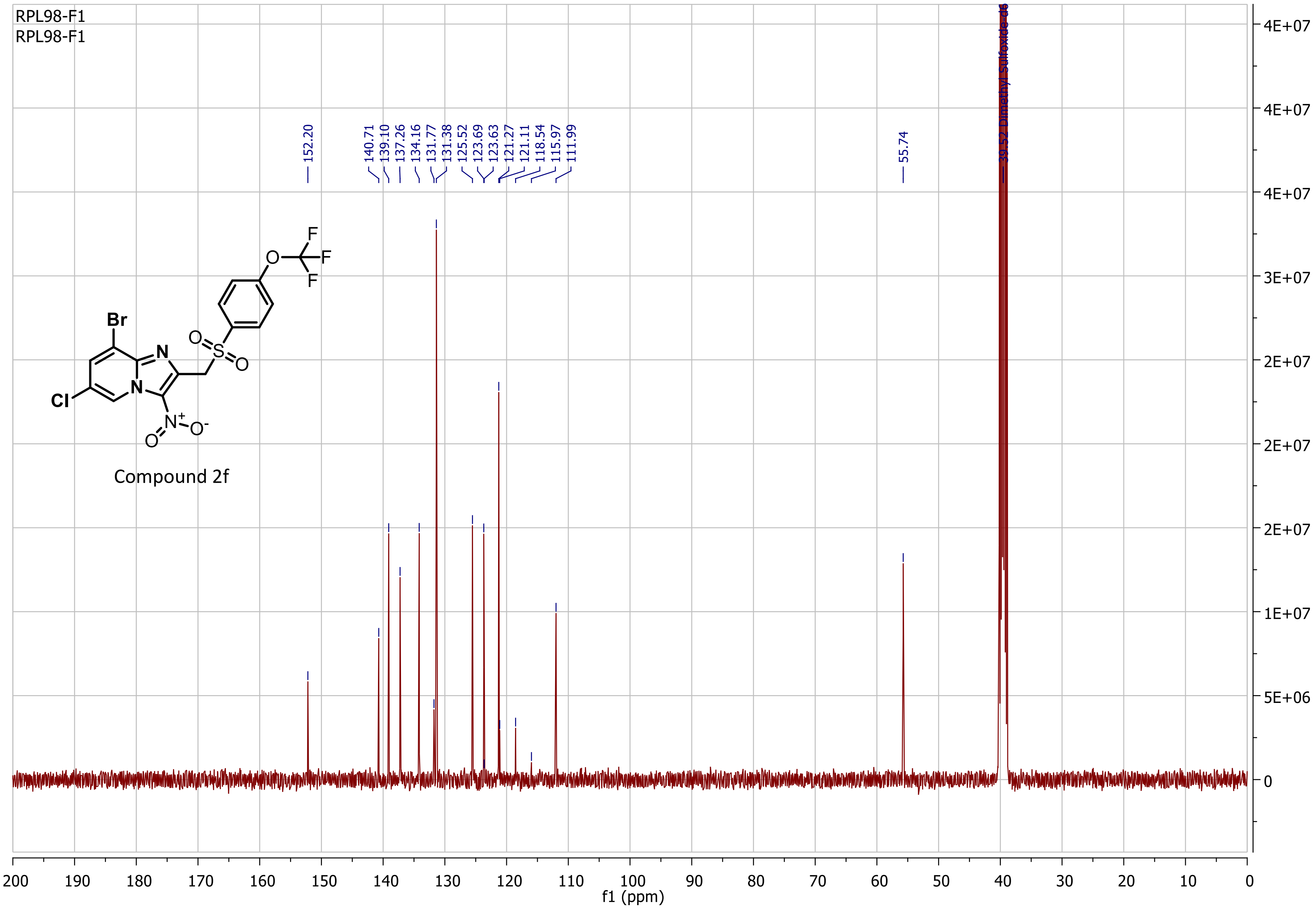
RPL98-F1
RPL98-F1
152.20
140.71
139.10
137.26
134.16
131.77
131.38
125.52
123.69
123.63
121.27
121.11
118.54
115.97
111.99
55.74
39.52 Dimethyl Sulfoxide-d6
4E+07
4E+07
4E+07
3E+07
2E+07
2E+07
2E+07
1E+07
5E+06
0
200 190 180 170 160 150 140 130 120 110 100 90 80 70 60 50 40 30 20 10 0
f1 (ppm)
Br
Cl
N
N
O
S
O
O
F
F
F
N+
O-
O

Compound 2f

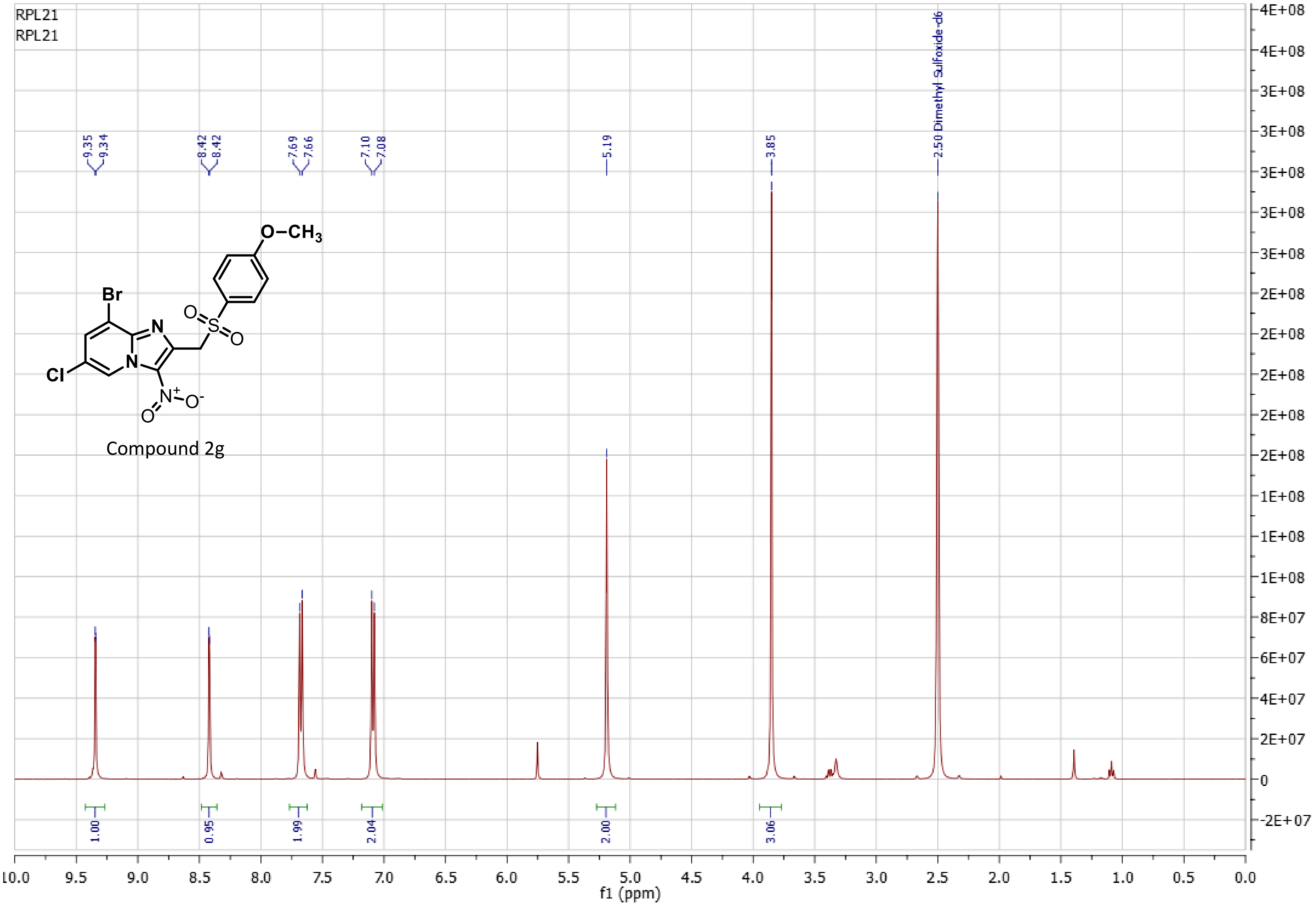
RPL21
RPL21
9.35
9.34
8.42
8.42
7.69
7.66
7.10
7.08
5.19
3.85
2.50 Dimethyl Sulfoxide-d6
O−CH3
Br
N
O
S
O
Cl
N
N+
O-
O
Compound 2g
1.00
0.95
1.99
2.04
2.00
3.06
f1 (ppm)

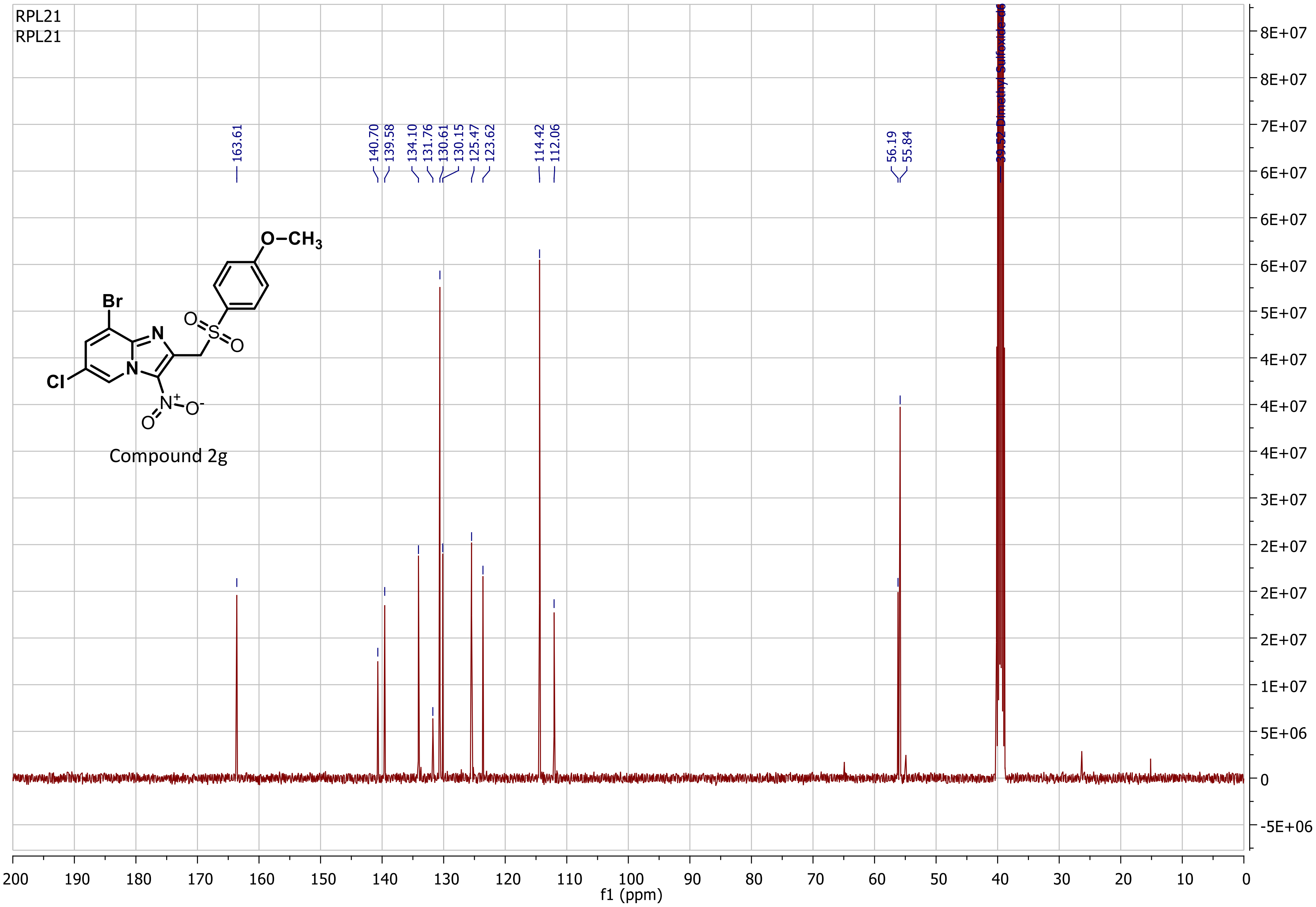
RPL21
RPL21
163.61
140.70
139.58
134.10
131.76
130.61
130.15
125.47
123.62
114.42
112.06
56.19
55.84
39.52 Dimethyl Sulfoxide-d6
O−CH3
Br
N
O
S
O
N
Cl
N+
O-
O
Compound 2g
8E+07
8E+07
7E+07
6E+07
6E+07
6E+07
5E+07
4E+07
4E+07
4E+07
3E+07
2E+07
2E+07
2E+07
1E+07
5E+06
0
-5E+06
200
190
180
170
160
150
140
130
120
110
100
90
80
70
60
50
40
30
20
10
0
f1 (ppm)

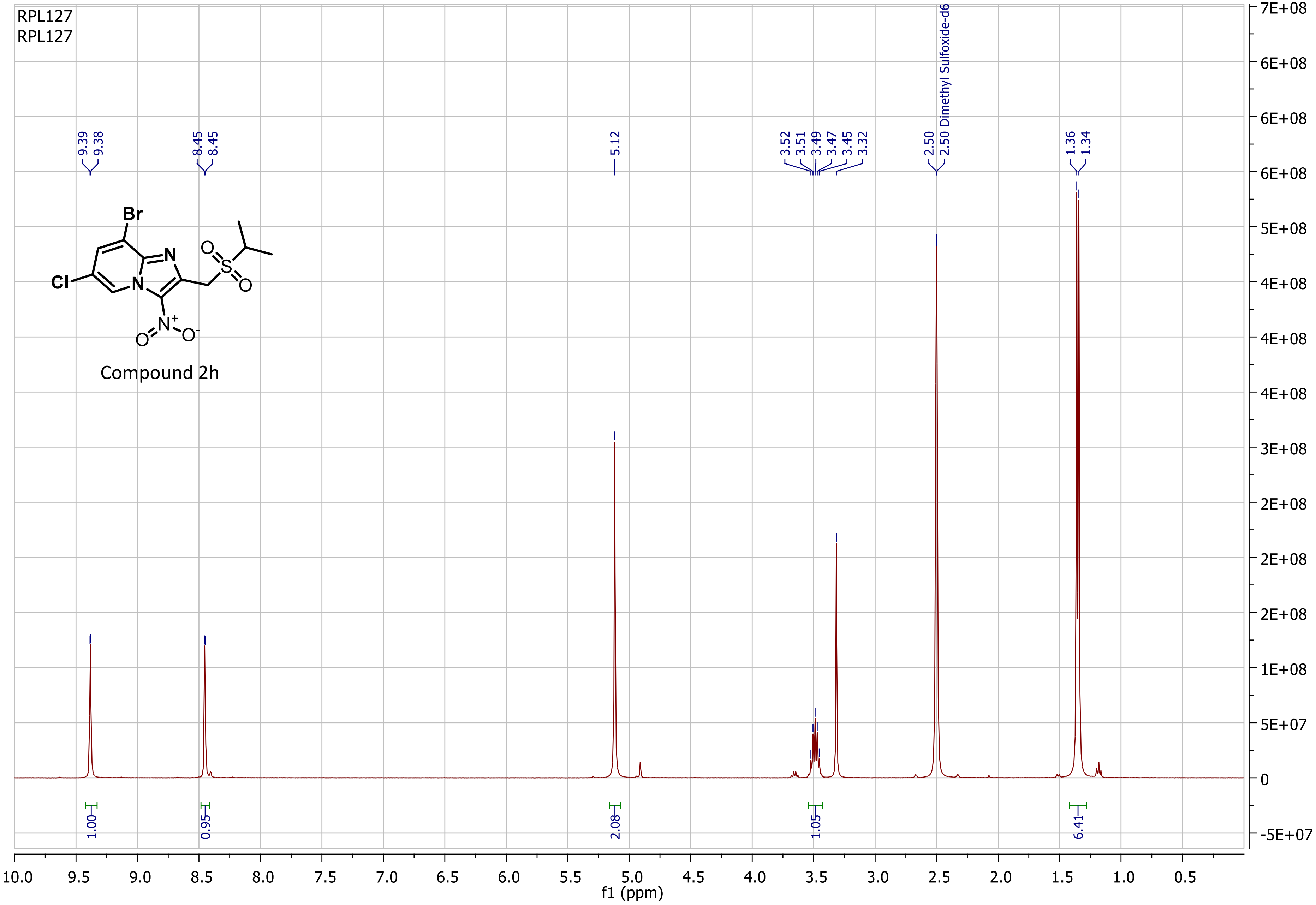
RPL127
RPL127
9.39
9.38
8.45
8.45
5.12
3.52
3.51
3.49
3.47
3.45
3.32
2.50
2.50 Dimethyl Sulfoxide-d6
1.36
1.34
Compound 2h
1.00
0.95
2.08
1.05
6.41
f1 (ppm)

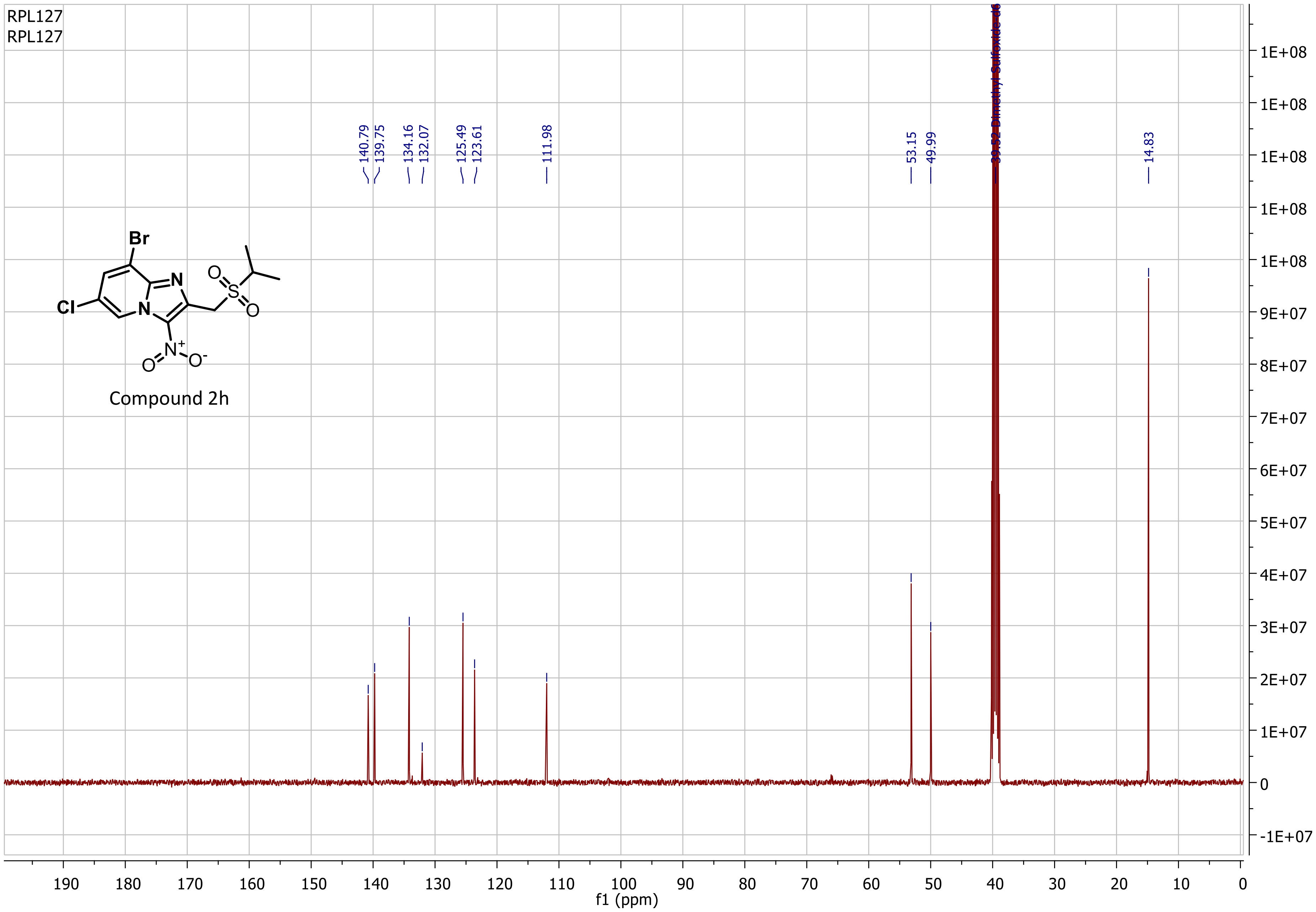

RPL127
RPL127
140.79
139.75
134.16
132.07
125.49
123.61
111.98
53.15
49.99
39.52 Dimethyl Sulfoxide-d6
14.83
Br
N
Cl
N
O
S
O
N+
O
O-
Compound 2h
f1 (ppm)

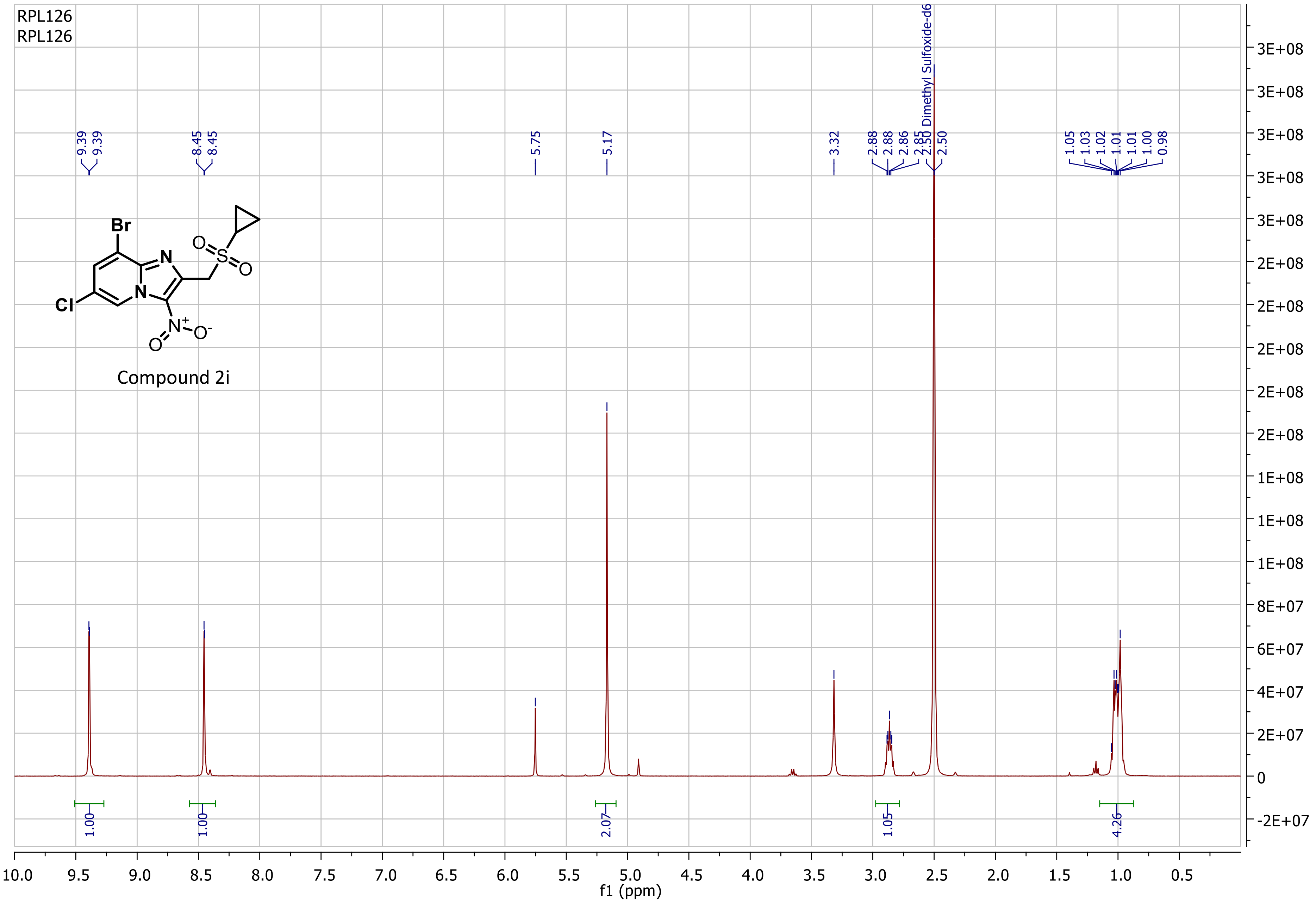
RPL126
RPL126
9.39
9.39
8.45
8.45
5.75
5.17
3.32
2.88
2.88
2.86
2.85
2.50 Dimethyl Sulfoxide-d6
2.50
1.05
1.03
1.02
1.01
1.01
1.00
0.98
Br
N
O
S
O
Cl
N
N+
O-
O
Compound 2i
1.00
1.00
2.07
1.05
4.26
f1 (ppm)

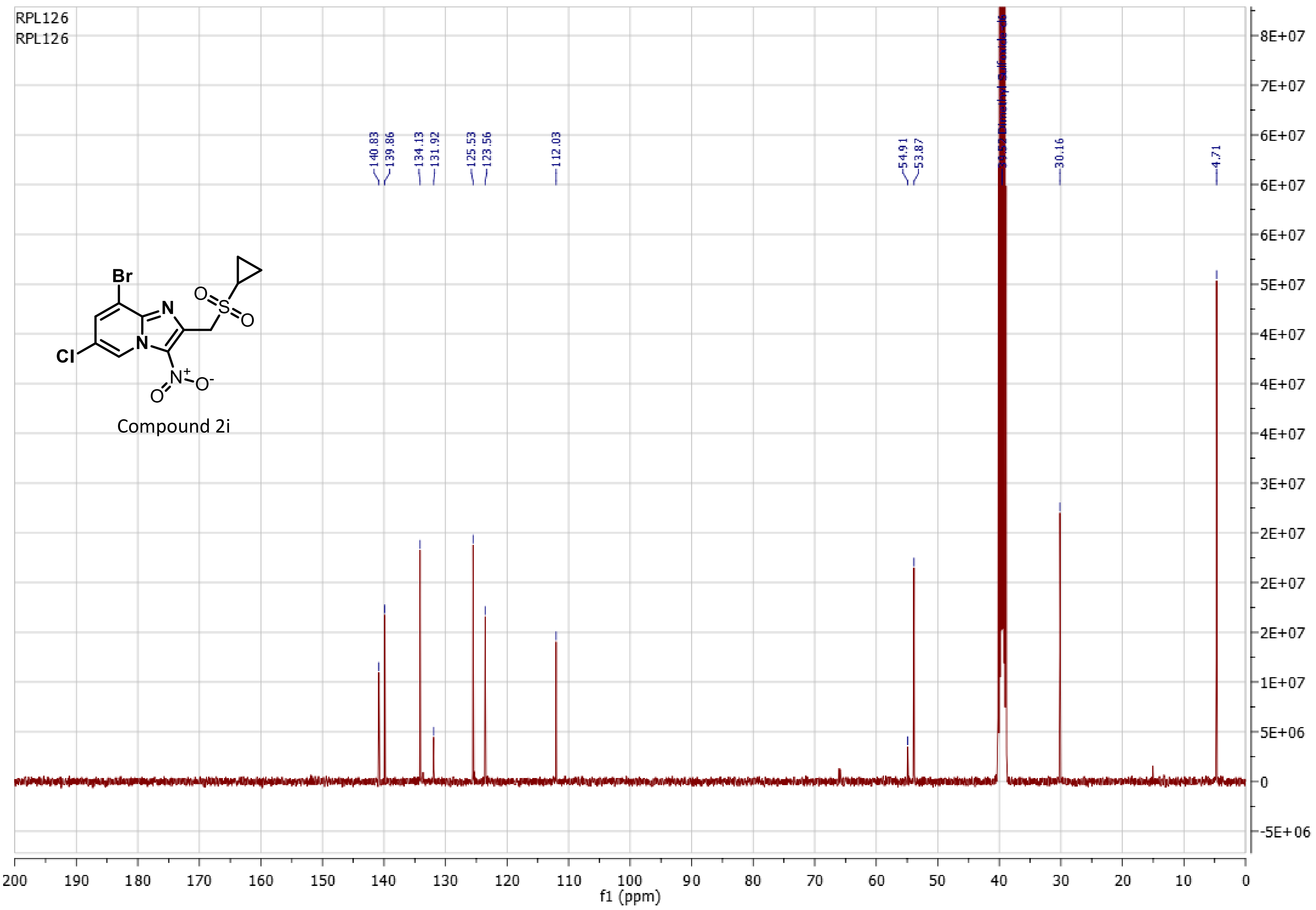
RPL126
RPL126
140.83
139.86
134.13
131.92
125.53
123.56
112.03
54.91
53.87
30.16
4.71
Br
Cl
N
N
O
S
O
N+
O-
O
f1 (ppm)

Compound 2i

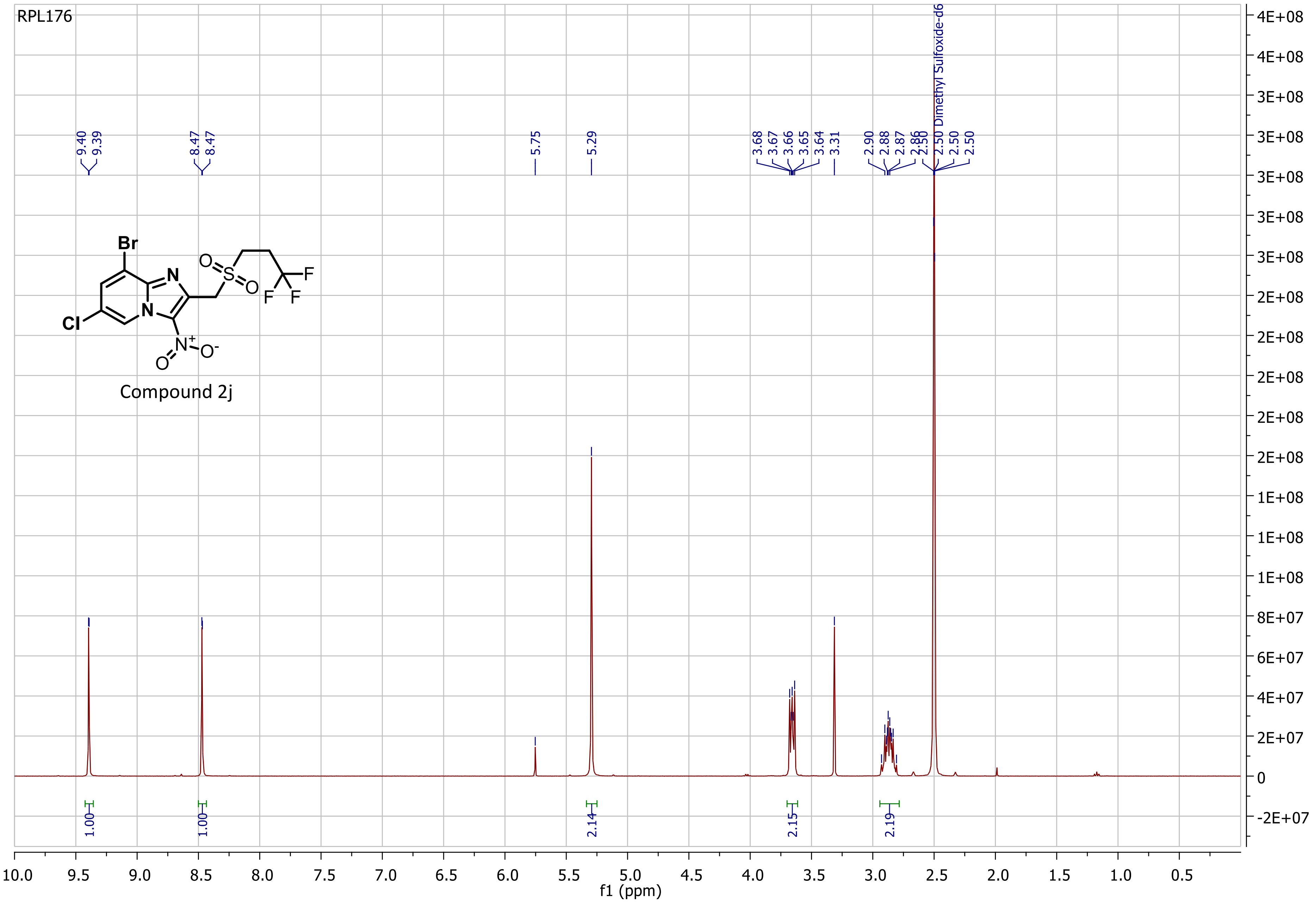

RPL176
9.40
9.39
8.47
8.47
5.75
5.29
3.68
3.67
3.66
3.65
3.64
3.31
2.90
2.88
2.87
2.86
2.50
2.50 Dimethyl Sulfoxide-d6
2.50
2.50
Br
N
O
S
O
F
F
F
Cl
N
N
O
O
Compound 2j
1.00
1.00
2.14
2.15
2.19
4E+08
3E+08
2E+08
1E+08
8E+07
6E+07
4E+07
2E+07
0
-2E+07
10.0
9.5
9.0
8.5
8.0
7.5
7.0
6.5
6.0
5.5
5.0
4.5
4.0
3.5
3.0
2.5
2.0
1.5
1.0
0.5
f1 (ppm)

RPL176
RPL176

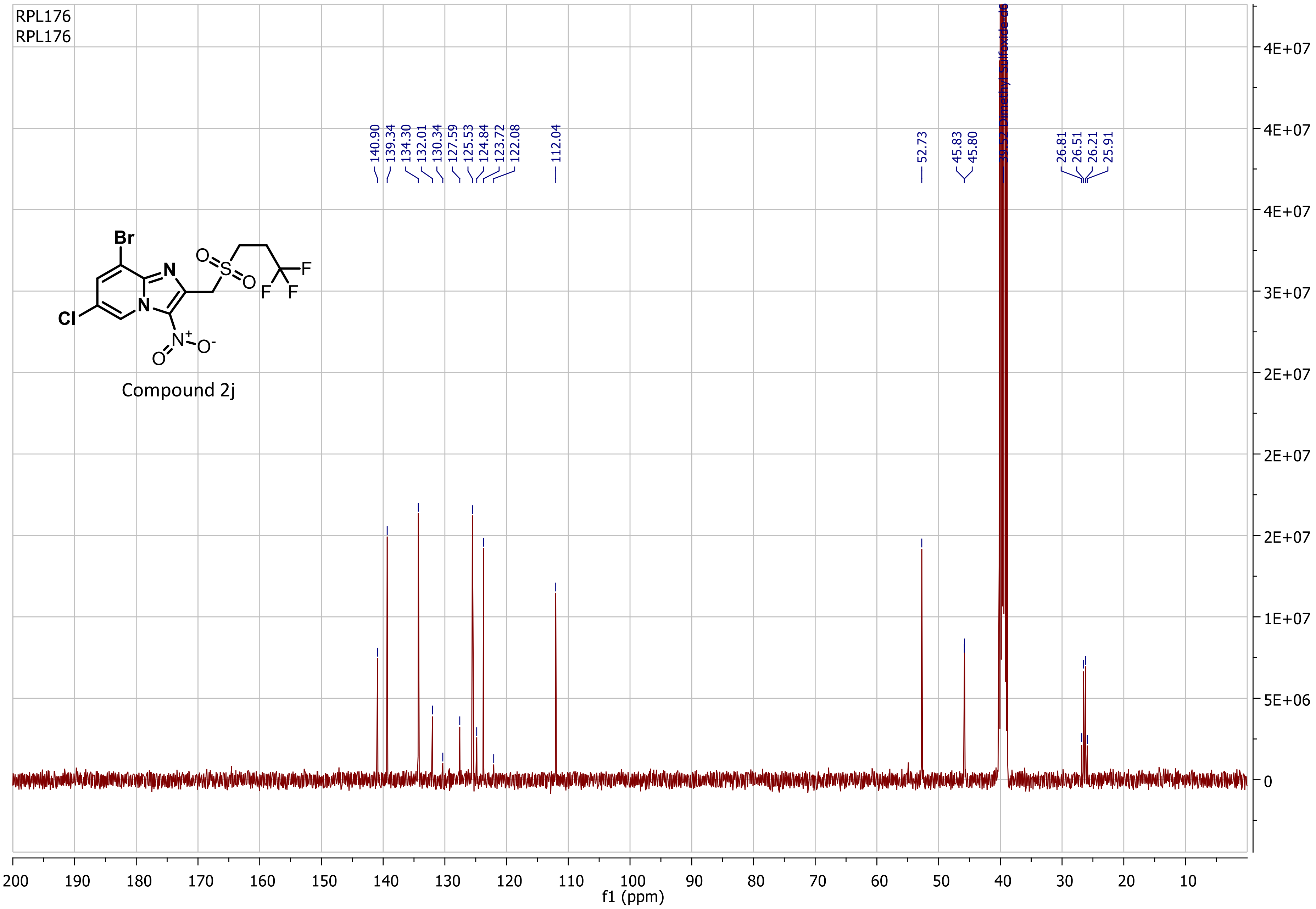

Compound 2j

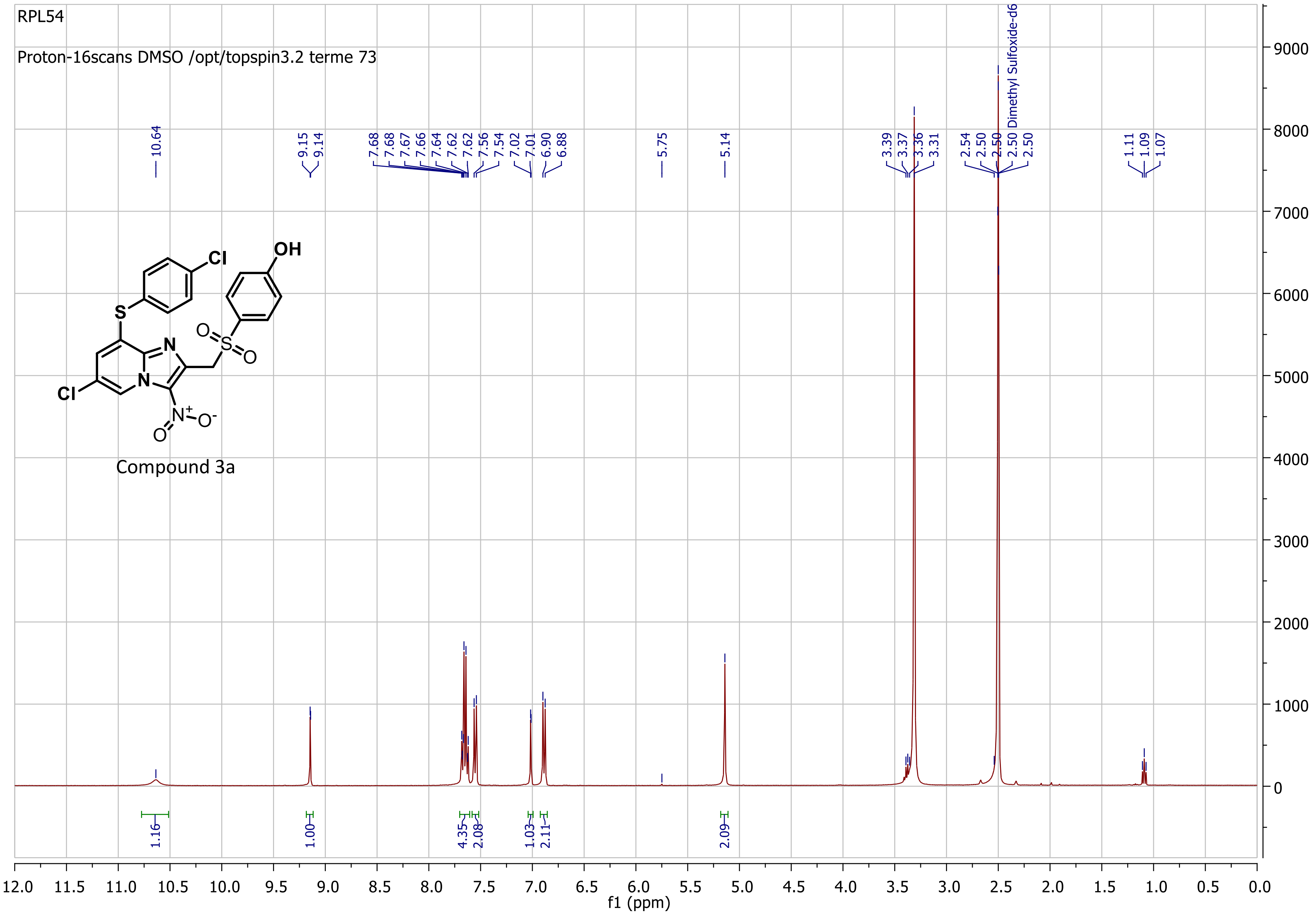
RPL54
Proton-16scans DMSO /opt/topspin3.2 terme 73
10.64
9.15 9.14
7.68 7.68 7.67 7.66 7.64 7.62 7.62 7.56 7.54 7.02 7.01 6.90 6.88
5.75
5.14
3.39 3.37 3.36 3.31
2.54 2.50 2.50 2.50 Dimethyl Sulfoxide-d6 2.50
1.11 1.09 1.07
OH
Cl
S
N
O
O
Cl
N
N
O
O
Compound 3a
1.16
1.00
4.35
2.08
1.03
2.11
2.09
f1 (ppm)

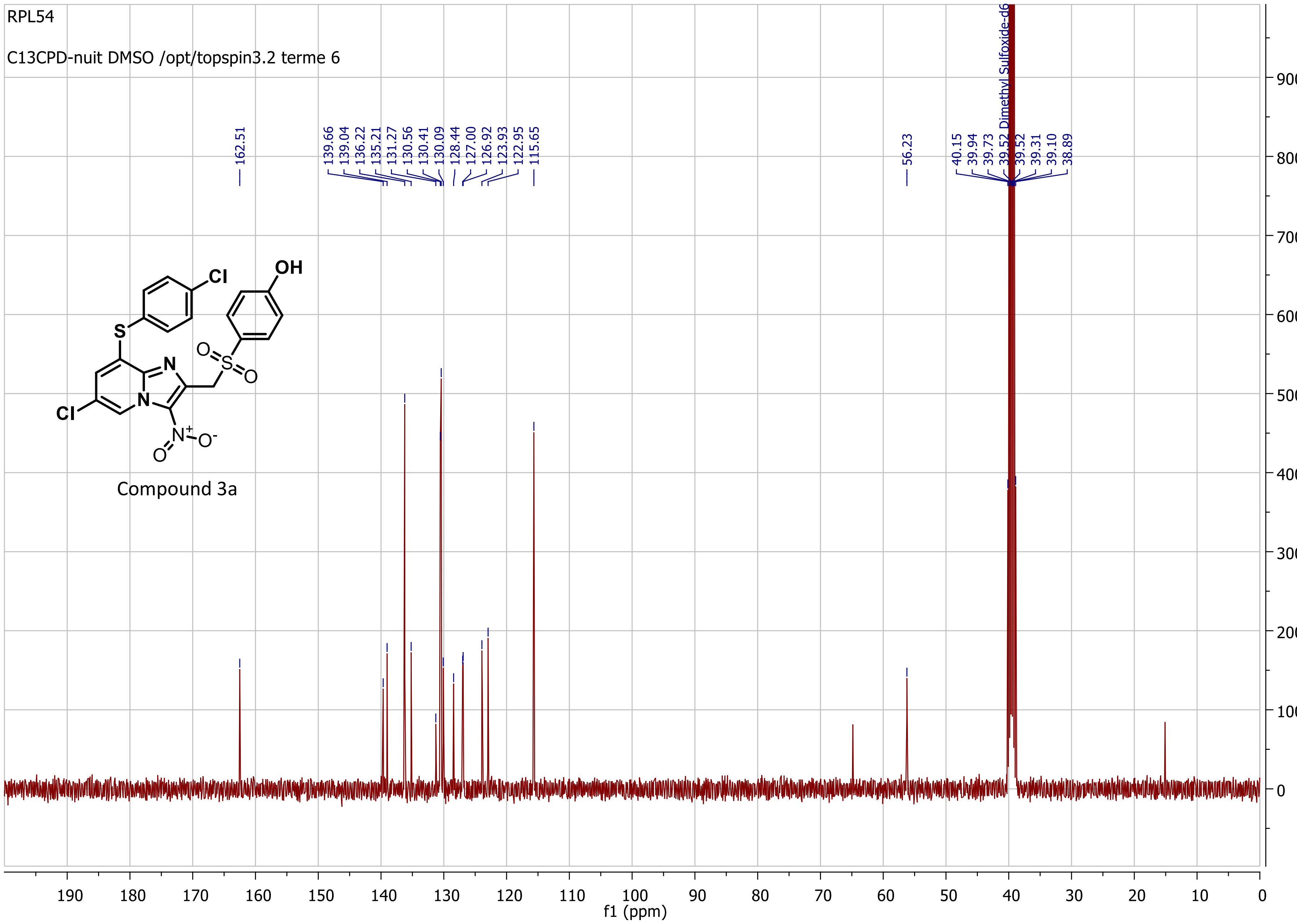
RPL54
C13CPD-nuit DMSO /opt/topspin3.2 terme 6
162.51
139.66
139.04
136.22
135.21
131.27
130.56
130.41
130.09
128.44
127.00
126.92
123.93
122.95
115.65
56.23
40.15
39.94
39.73
39.52 Dimethyl Sulfoxide-d6
39.52
39.31
39.10
38.89
Compound 3a
f1 (ppm)

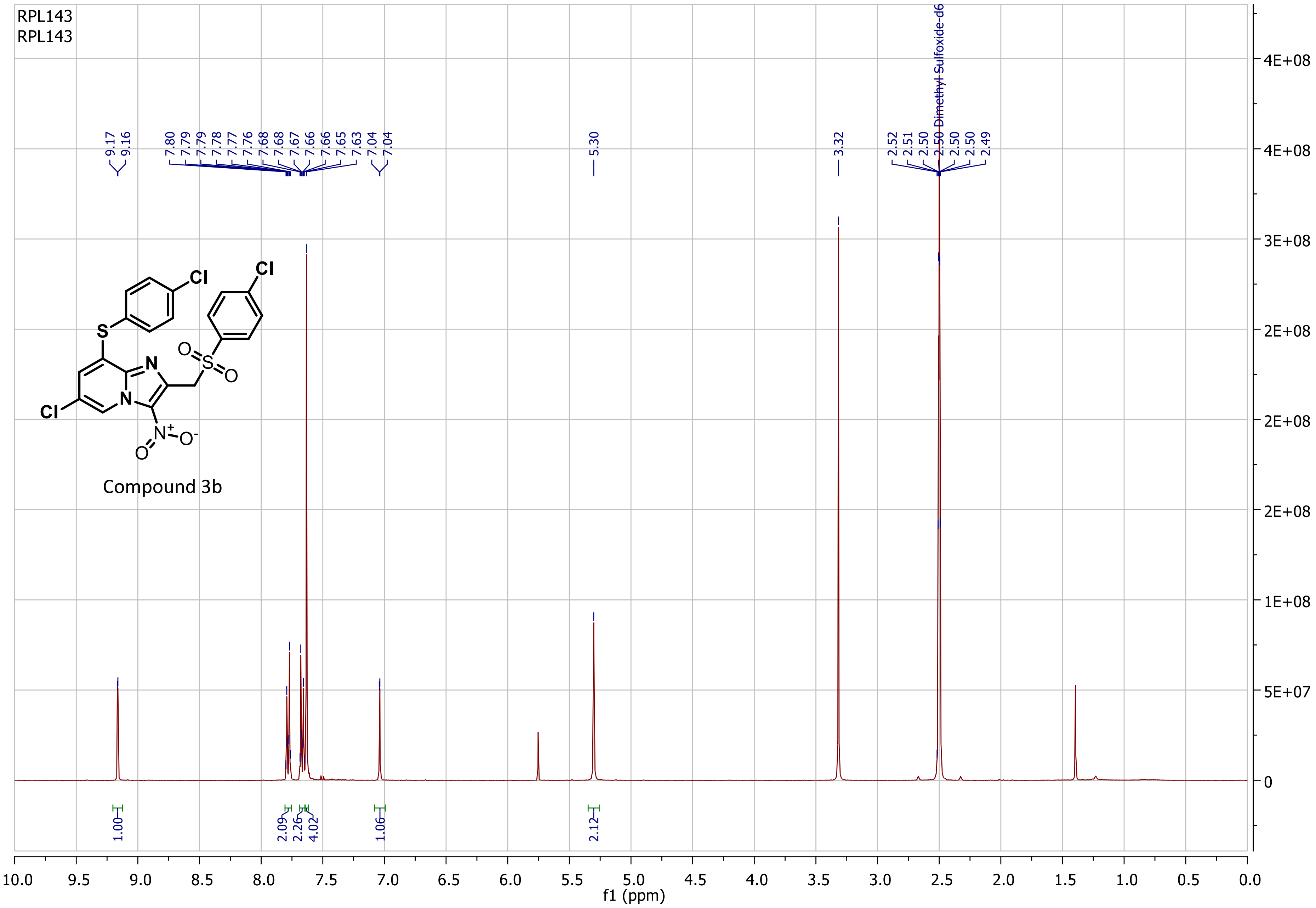

RPL143
RPL143
9.17
9.16
7.80
7.79
7.79
7.78
7.77
7.76
7.68
7.68
7.67
7.66
7.66
7.65
7.63
7.04
7.04
5.30
3.32
2.52
2.51
2.50
2.50 Dimethyl Sulfoxide-d6
2.50
2.50
2.49
Cl
Cl
S
O
S
O
N
Cl
N
N
O
O
Compound 3b
1.00
2.09
2.26
4.02
1.06
2.12
4E+08
4E+08
3E+08
2E+08
2E+08
2E+08
1E+08
5E+07
0
10.0
9.5
9.0
8.5
8.0
7.5
7.0
6.5
6.0
5.5
5.0
4.5
4.0
3.5
3.0
2.5
2.0
1.5
1.0
0.5
0.0
f1 (ppm)

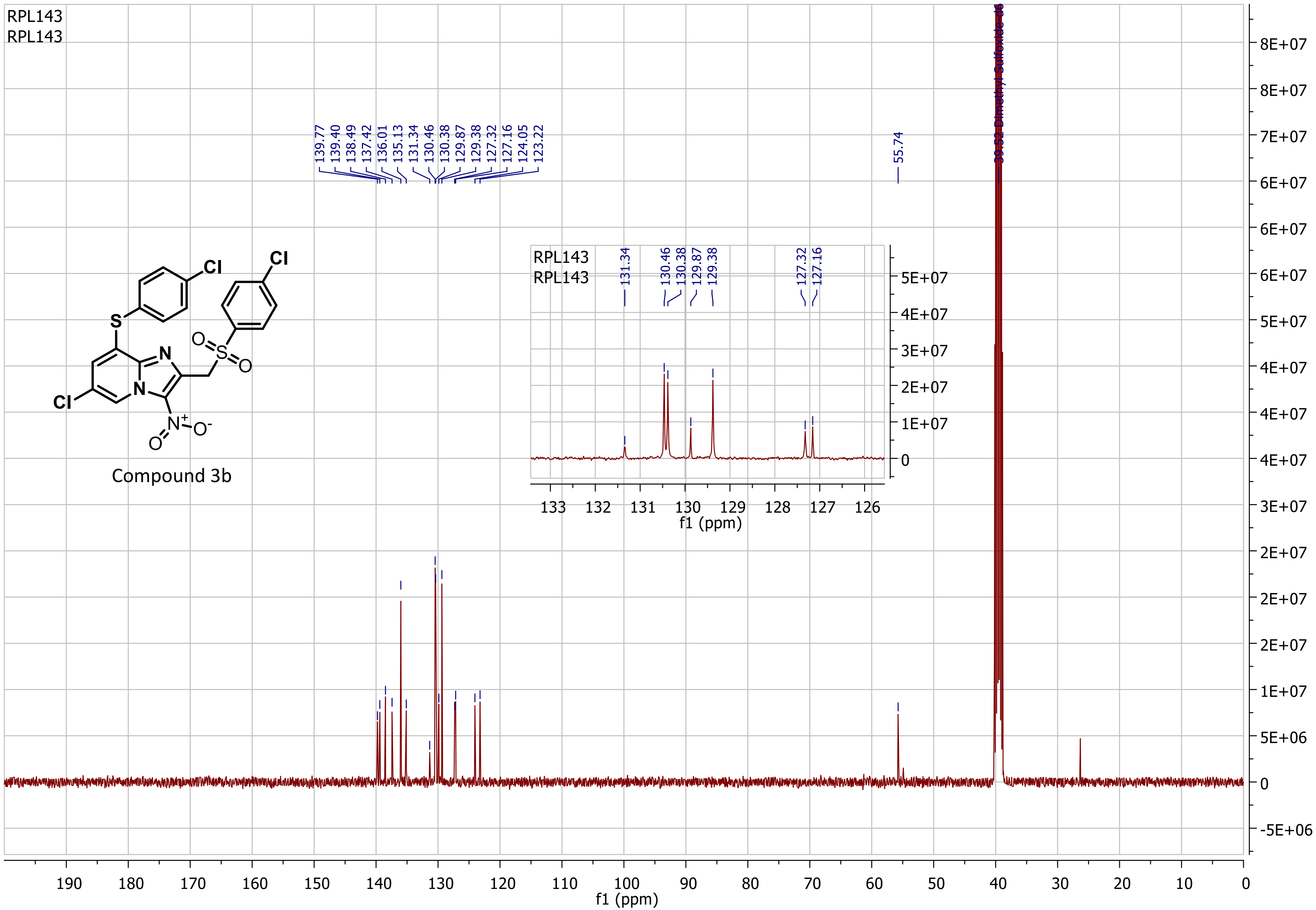
RPL143
RPL143
139.77
139.40
138.49
137.42
136.01
135.13
131.34
130.46
130.38
129.87
129.38
127.32
127.16
124.05
123.22
55.74
39.52 Dimethyl Sulfoxide-d6
Compound 3b
f1 (ppm)

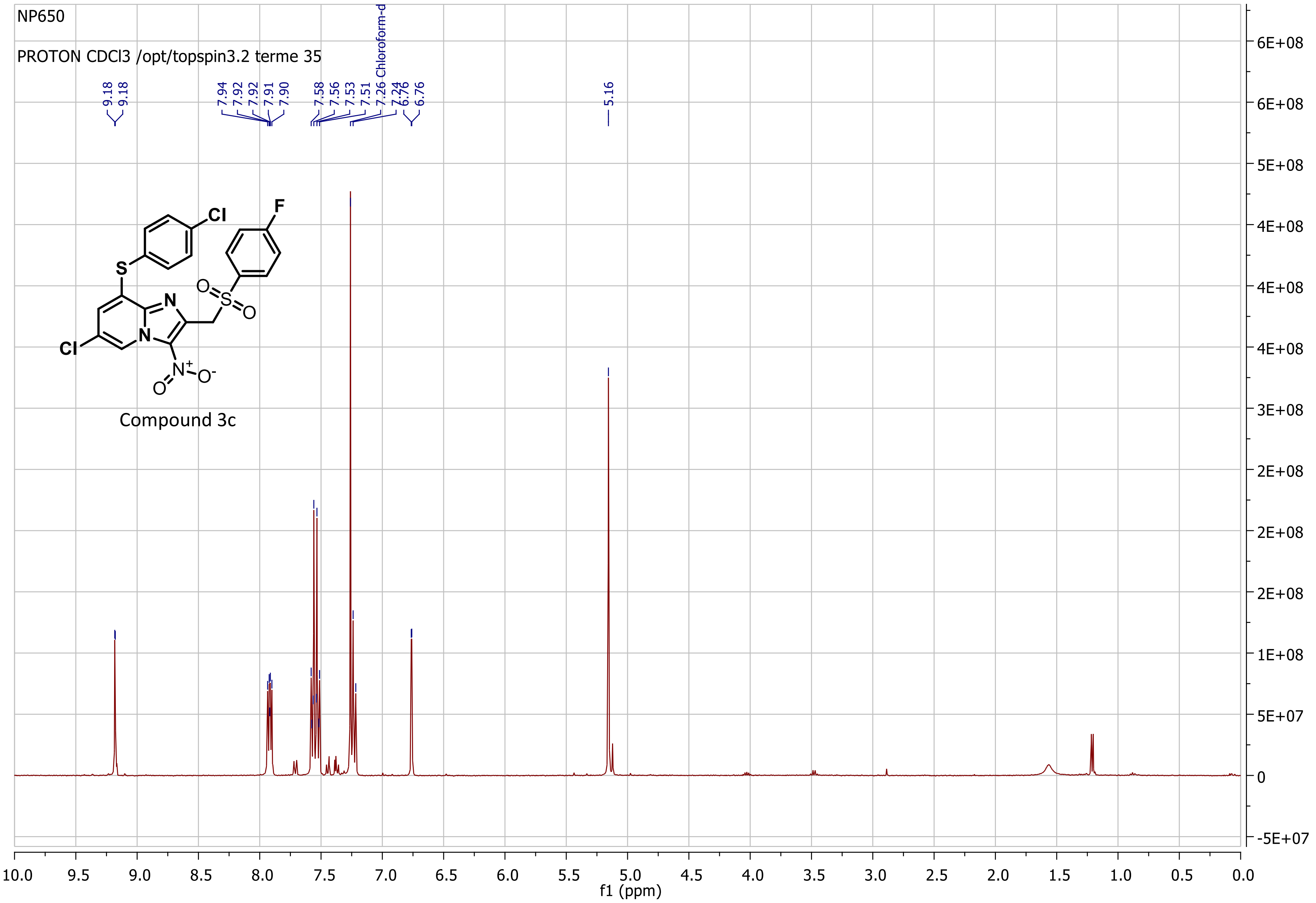

NP650
PROTON CDCl3 /opt/topspin3.2 terme 35
9.18
9.18
7.94
7.92
7.92
7.91
7.90
7.58
7.56
7.53
7.51
7.26 Chloroform-d
7.24
6.76
6.76
5.16
Cl
F
S
O
S
O
N
N
Cl
N+
O-
O
Compound 3c
6E+08
6E+08
5E+08
4E+08
4E+08
4E+08
3E+08
2E+08
2E+08
2E+08
1E+08
5E+07
0
-5E+07
10.0
9.5
9.0
8.5
8.0
7.5
7.0
6.5
6.0
5.5
5.0
4.5
4.0
3.5
3.0
2.5
2.0
1.5
1.0
0.5
0.0
f1 (ppm)

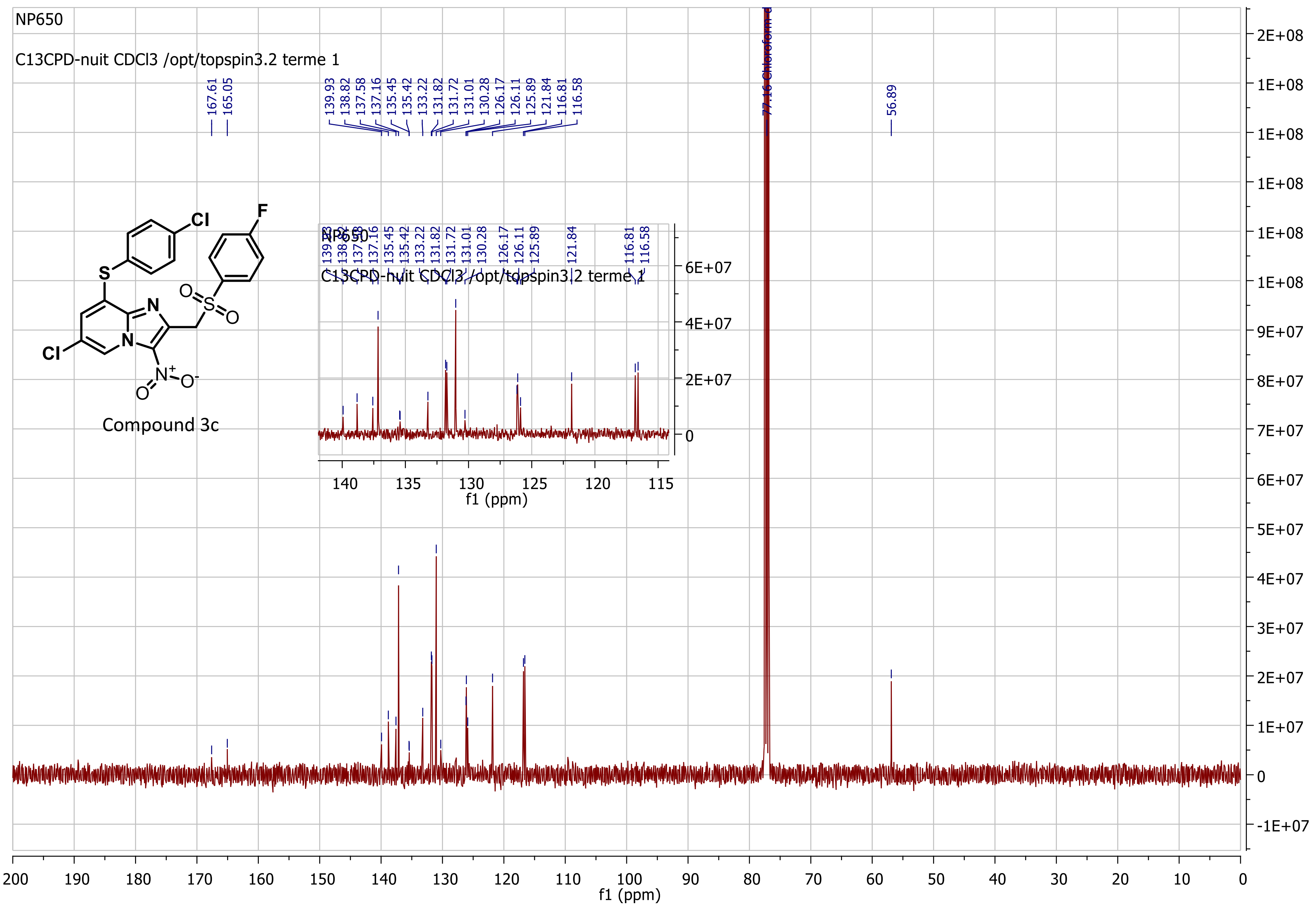
NP650
C13CPD-nuit CDCl3 /opt/topspin3.2 terme 1
167.61
165.05
139.93
138.82
137.58
137.16
135.45
135.42
133.22
131.82
131.72
131.01
130.28
126.17
126.11
125.89
121.84
116.81
116.58
77.16 Chloroform-d
56.89
Cl
F
S
N
O
S
O
N
Cl
N
O
O
Compound 3c
f1 (ppm)

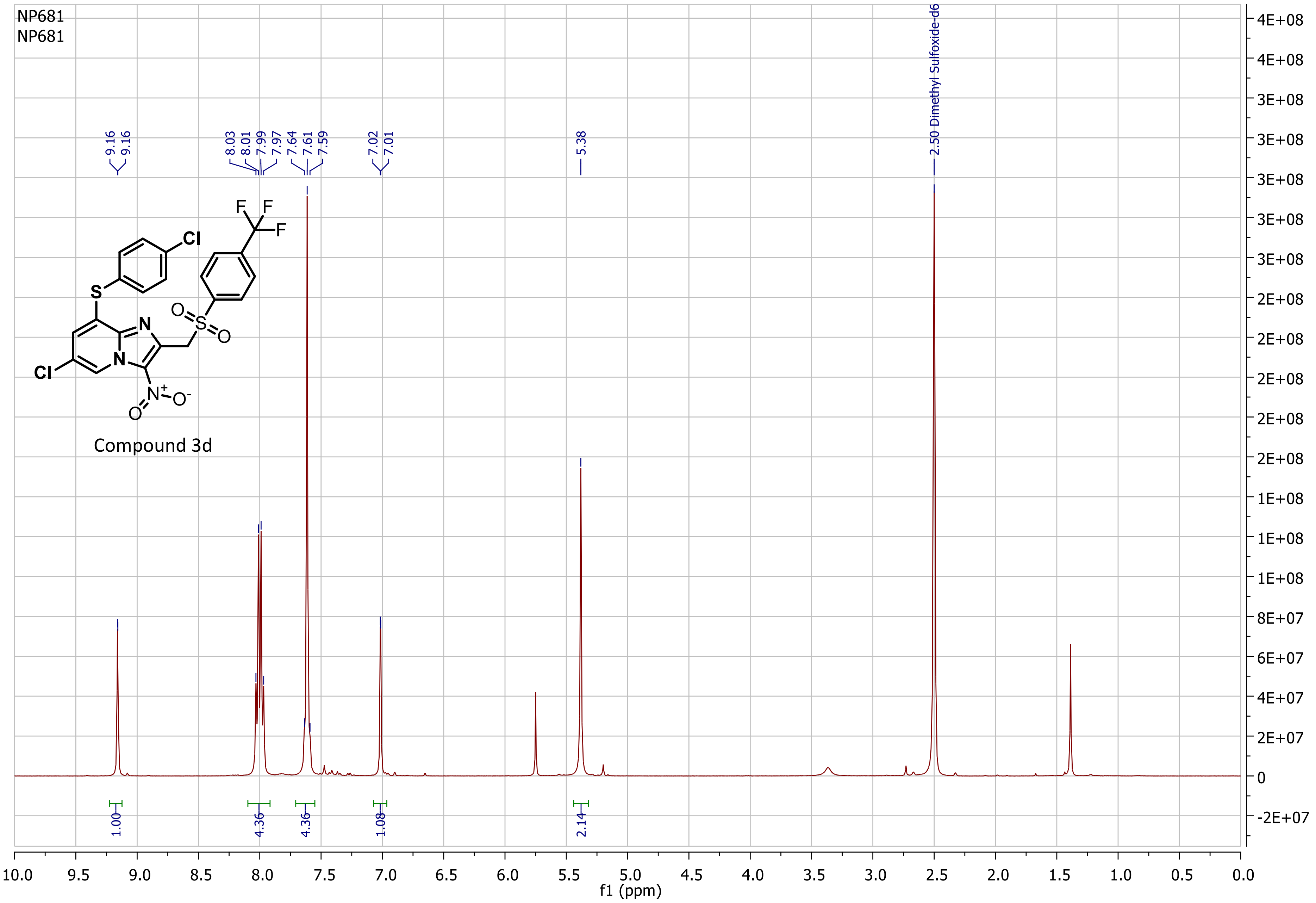
NP681
NP681
9.16
9.16
8.03
8.01
7.99
7.97
7.64
7.61
7.59
7.02
7.01
5.38
2.50 Dimethyl Sulfoxide-d6
Compound 3d
1.00
4.36
4.36
1.08
2.14
f1 (ppm)

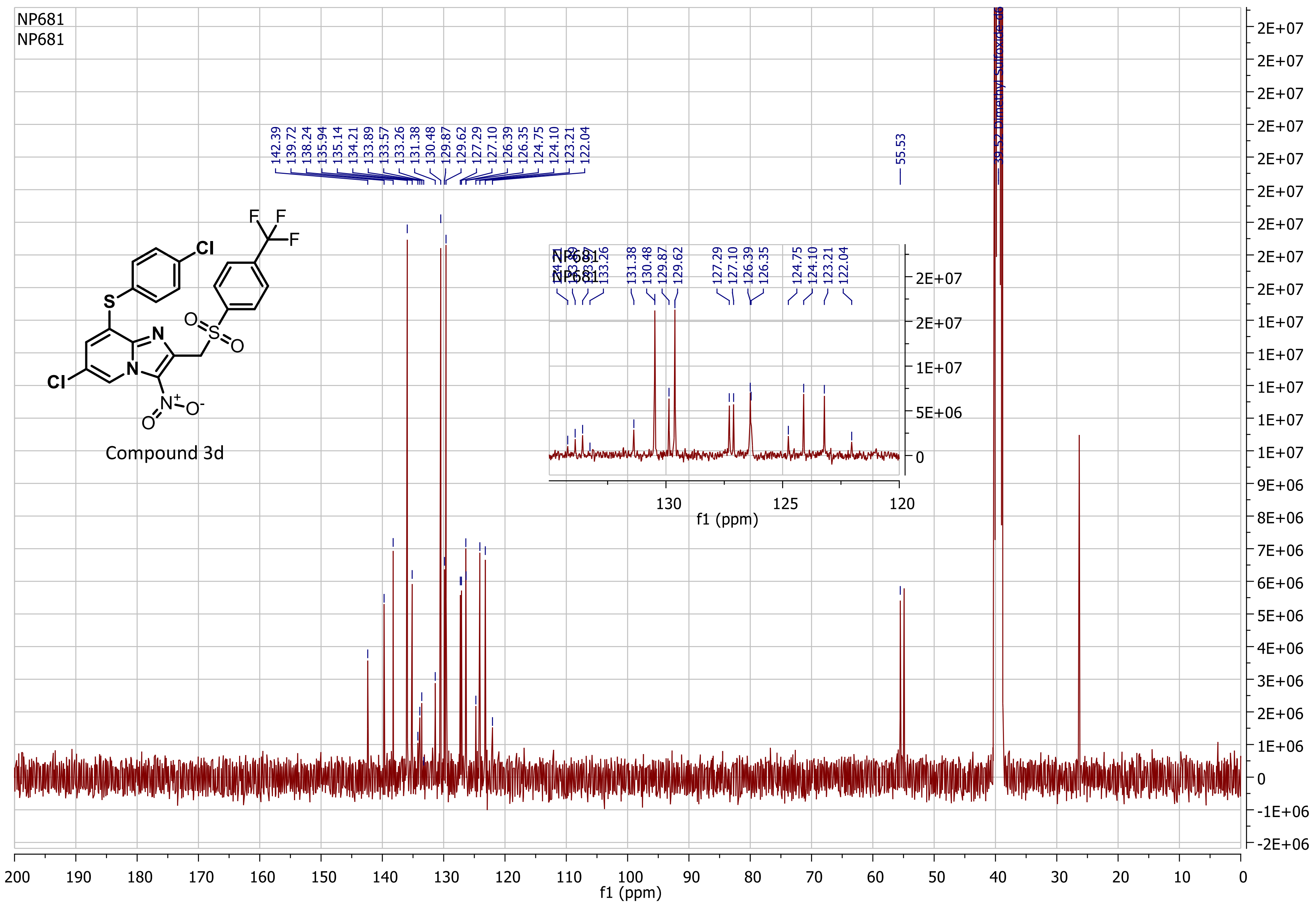

NP681
NP681
142.39
139.72
138.24
135.94
135.14
134.21
133.89
133.57
133.26
131.38
130.48
129.87
129.62
127.29
127.10
126.39
126.35
124.75
124.10
123.21
122.04
55.53
39.52 Dimethyl Sulfoxide-d6
Compound 3d
f1 (ppm)

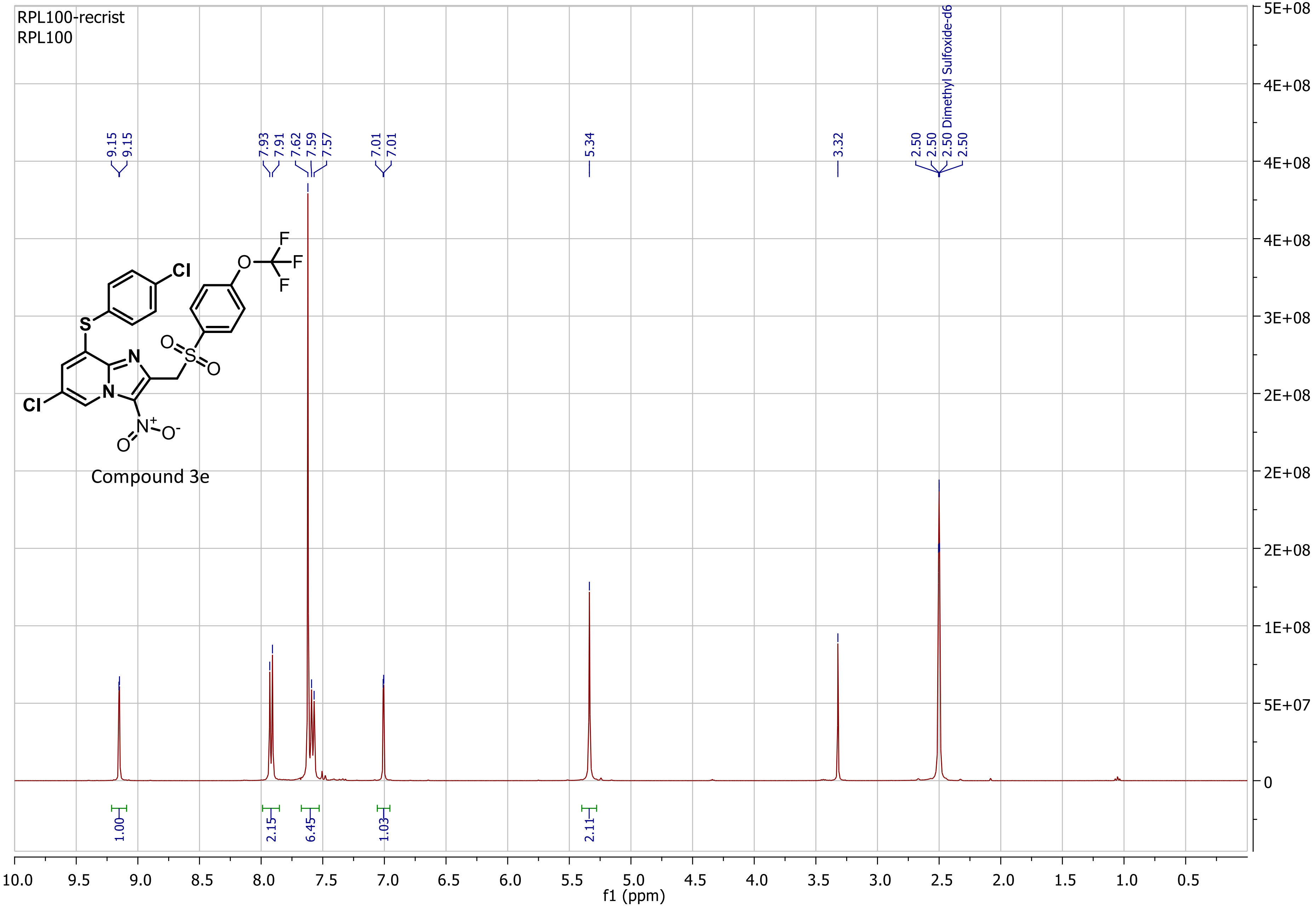
RPL100-recrist
RPL100
9.15
9.15
7.93
7.91
7.62
7.59
7.57
7.01
7.01
5.34
3.32
2.50
2.50
2.50 Dimethyl Sulfoxide-d6
2.50
Compound 3e
1.00
2.15
6.45
1.03
2.11
f1 (ppm)
5E+08
4E+08
3E+08
2E+08
1E+08
5E+07
0

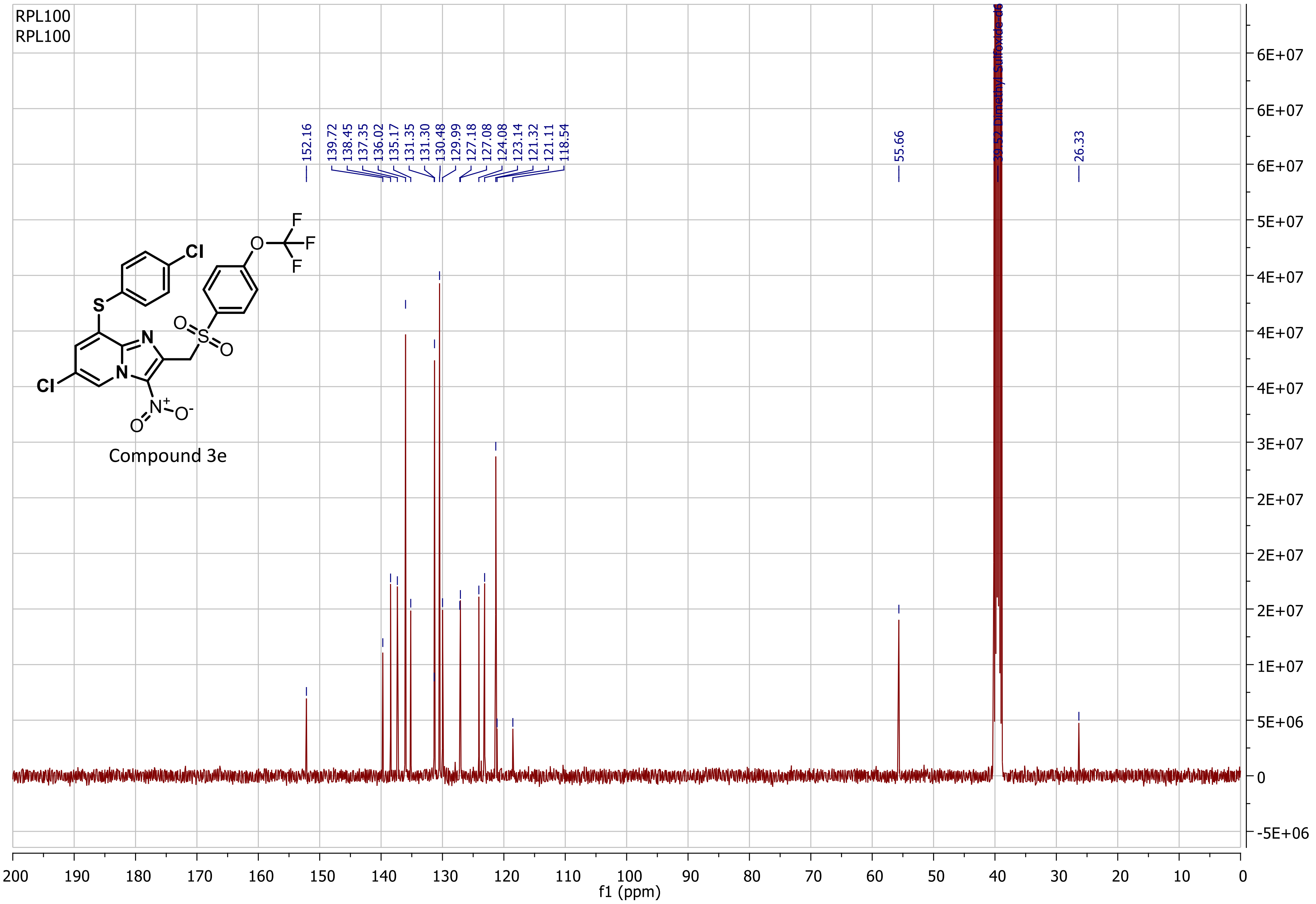

RPL100
RPL100
152.16
139.72
138.45
137.35
136.02
135.17
131.35
131.30
130.48
129.99
127.18
127.08
124.08
123.14
121.32
121.11
118.54
55.66
39.52 Dimethyl Sulfoxide-d6
26.33
Compound 3e
f1 (ppm)

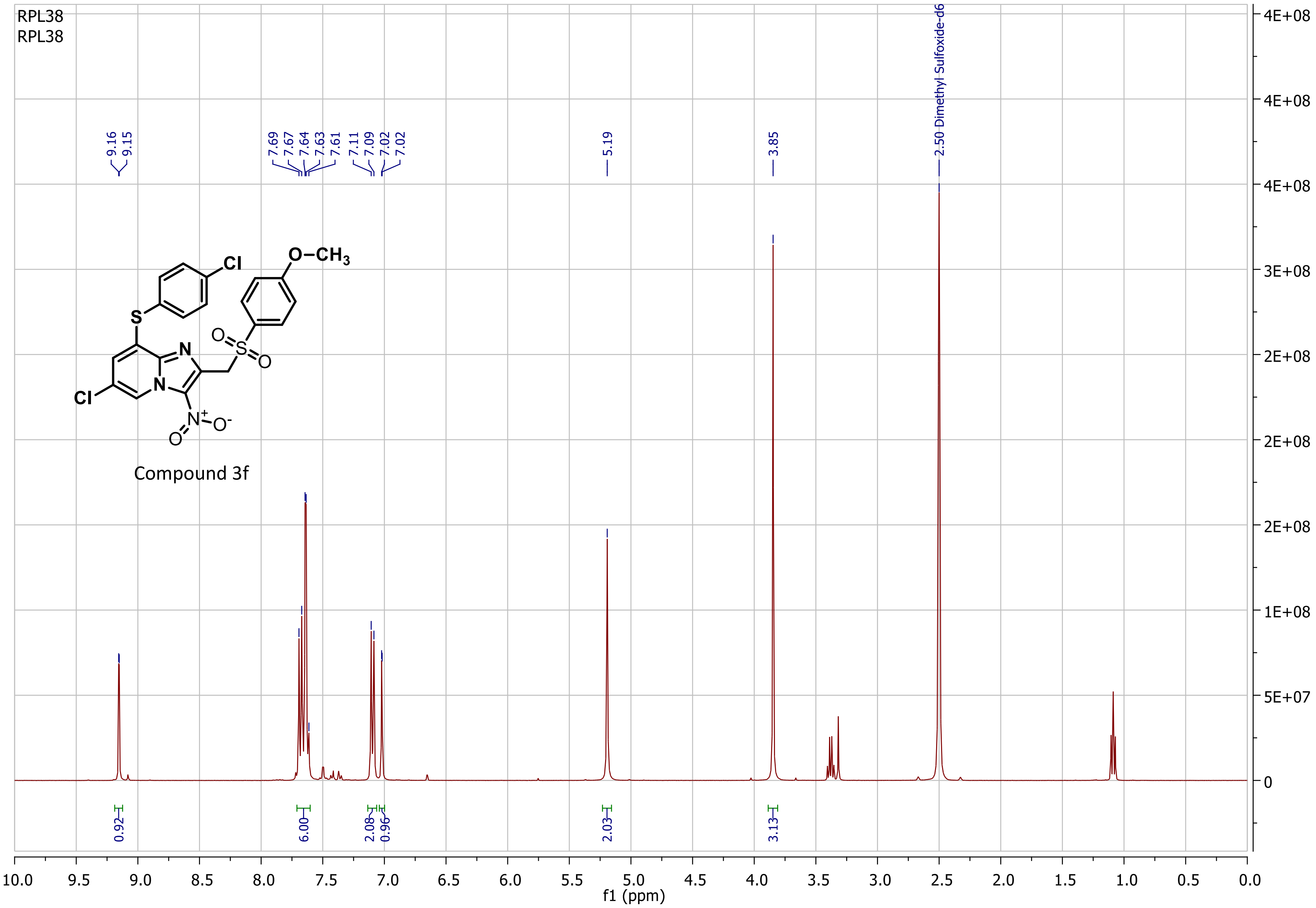
RPL38
RPL38
9.16
9.15
7.69
7.67
7.64
7.63
7.61
7.11
7.09
7.02
7.02
5.19
3.85
2.50 Dimethyl Sulfoxide-d6
Compound 3f
0.92
6.00
2.08
0.96
2.03
3.13
f1 (ppm)

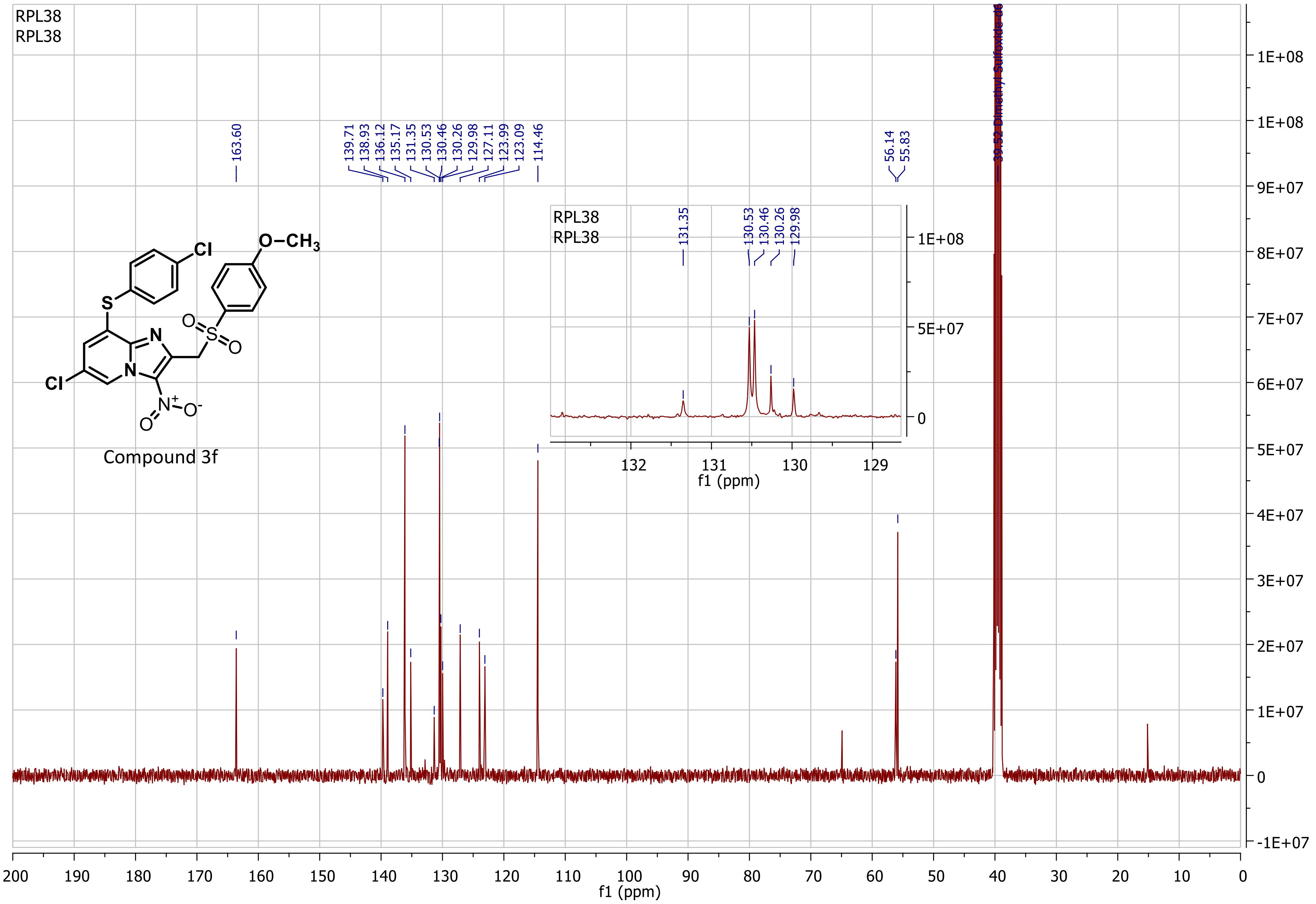
RPL38
RPL38
163.60
139.71
138.93
136.12
135.17
131.35
130.53
130.46
130.26
129.98
127.11
123.99
123.09
114.46
56.14
55.83
39.52 Dimethyl Sulfoxide-d6
O−CH3
Cl
S
O
S
O
N
N
Cl
N+
O-
O
Compound 3f
RPL38
RPL38
131.35
130.53
130.46
130.26
129.98
1E+08
5E+07
0
132
131
130
129
f1 (ppm)
1E+08
1E+08
9E+07
8E+07
7E+07
6E+07
5E+07
4E+07
3E+07
2E+07
1E+07
0
-1E+07
200
190
180
170
160
150
140
130
120
110
100
90
80
70
60
50
40
30
20
10
0
f1 (ppm)

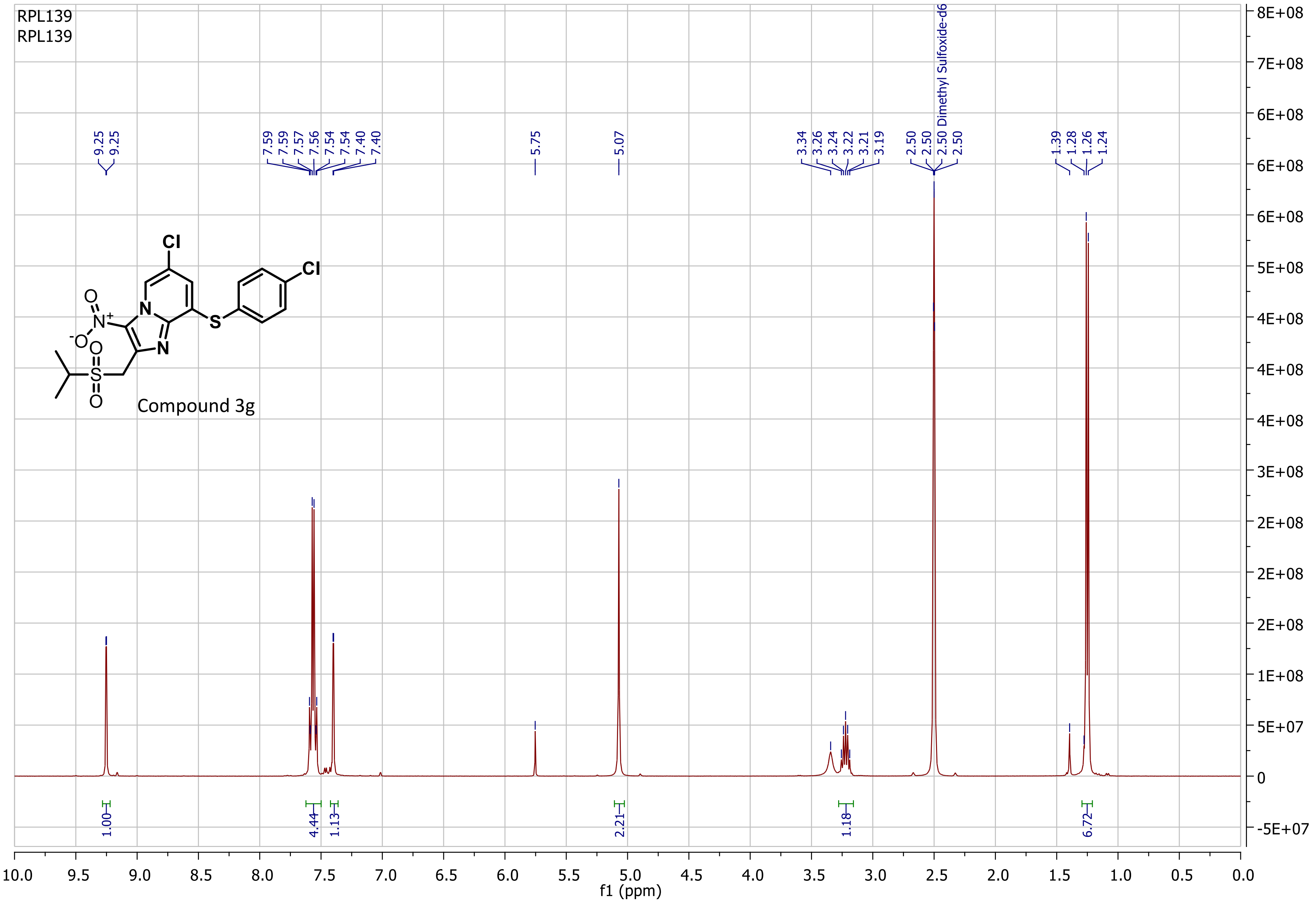
RPL139
RPL139
9.25
9.25
7.59
7.59
7.57
7.56
7.54
7.54
7.40
7.40
5.75
5.07
3.34
3.26
3.24
3.22
3.21
3.19
2.50
2.50
2.50 Dimethyl Sulfoxide-d6
2.50
1.39
1.28
1.26
1.24
Cl
Cl
S
N
N
O
N+
-O
O
S
O
Compound 3g
1.00
4.44
1.13
2.21
1.18
6.72
f1 (ppm)

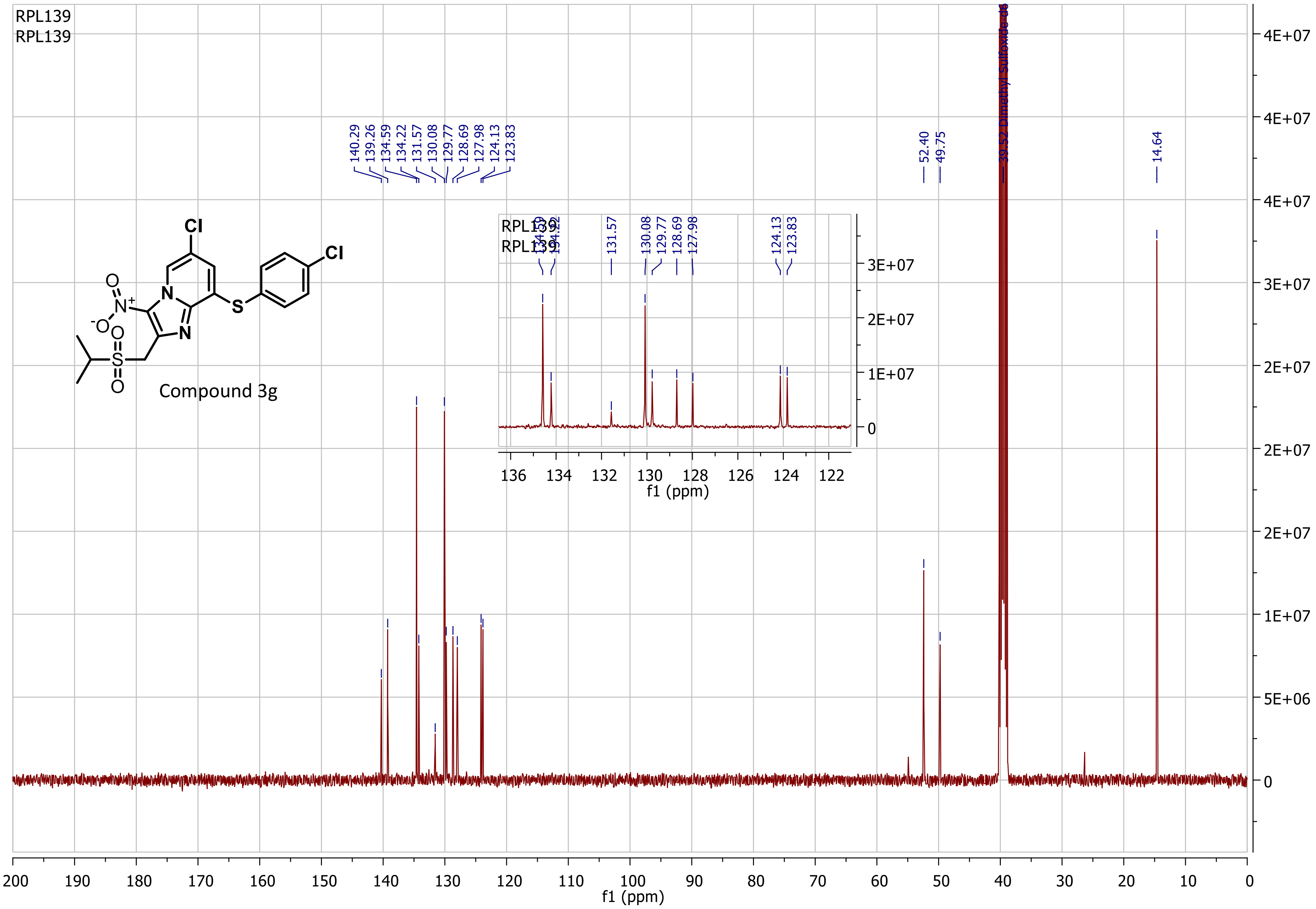

RPL139
RPL139
140.29
139.26
134.59
134.22
131.57
130.08
129.77
128.69
127.98
124.13
123.83
52.40
49.75
39.52 Dimethyl Sulfoxide-d6
14.64
Compound 3g
f1 (ppm)

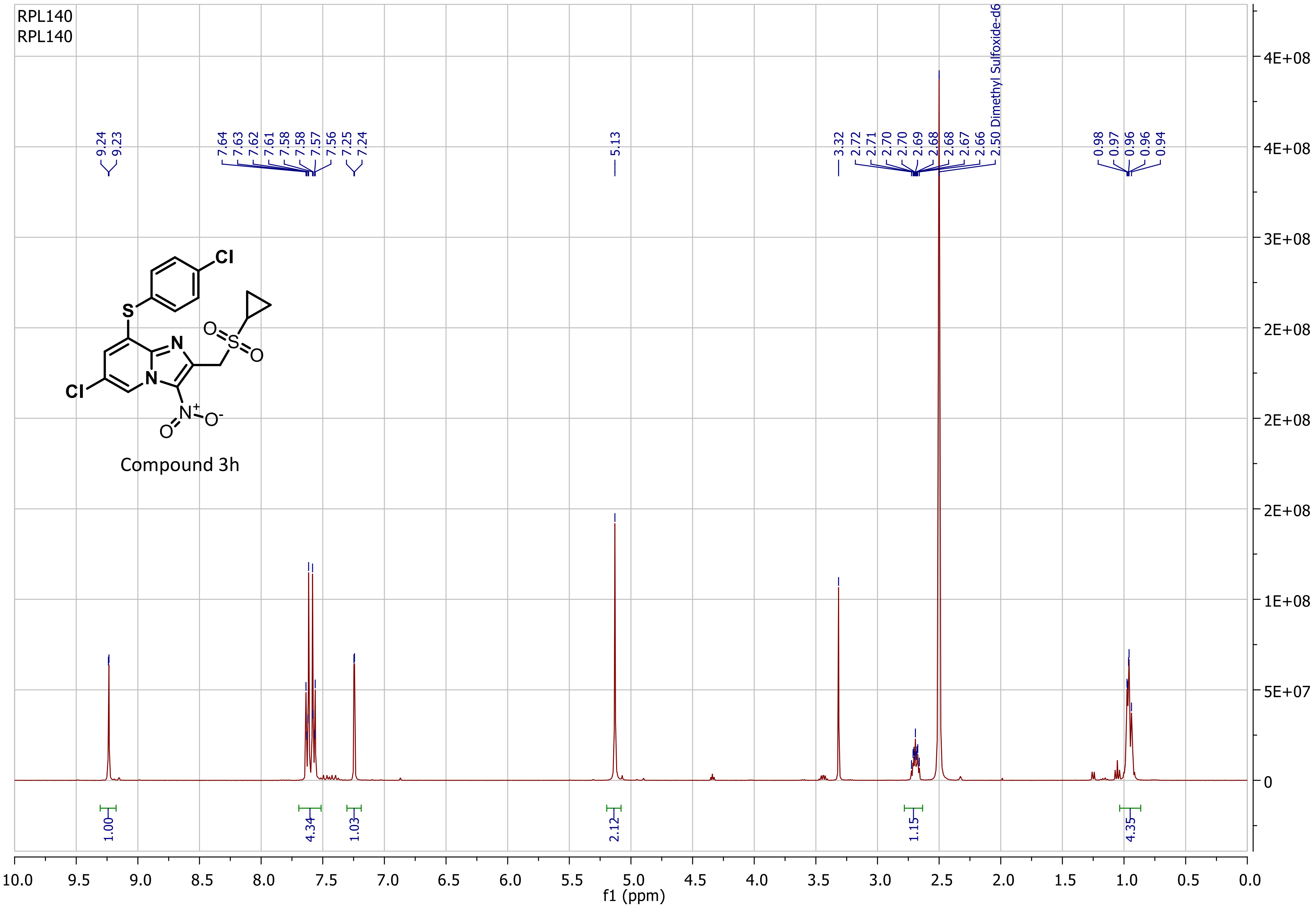

RPL140
RPL140
9.24
9.23
7.64
7.63
7.62
7.61
7.58
7.58
7.57
7.56
7.25
7.24
5.13
3.32
2.72
2.71
2.70
2.70
2.69
2.68
2.68
2.67
2.66
2.50 Dimethyl Sulfoxide-d6
0.98
0.97
0.96
0.96
0.94
Compound 3h
1.00
4.34
1.03
2.12
1.15
4.35
4E+08
4E+08
3E+08
2E+08
2E+08
2E+08
1E+08
5E+07
0
10.0
9.5
9.0
8.5
8.0
7.5
7.0
6.5
6.0
5.5
5.0
4.5
4.0
3.5
3.0
2.5
2.0
1.5
1.0
0.5
0.0
f1 (ppm)

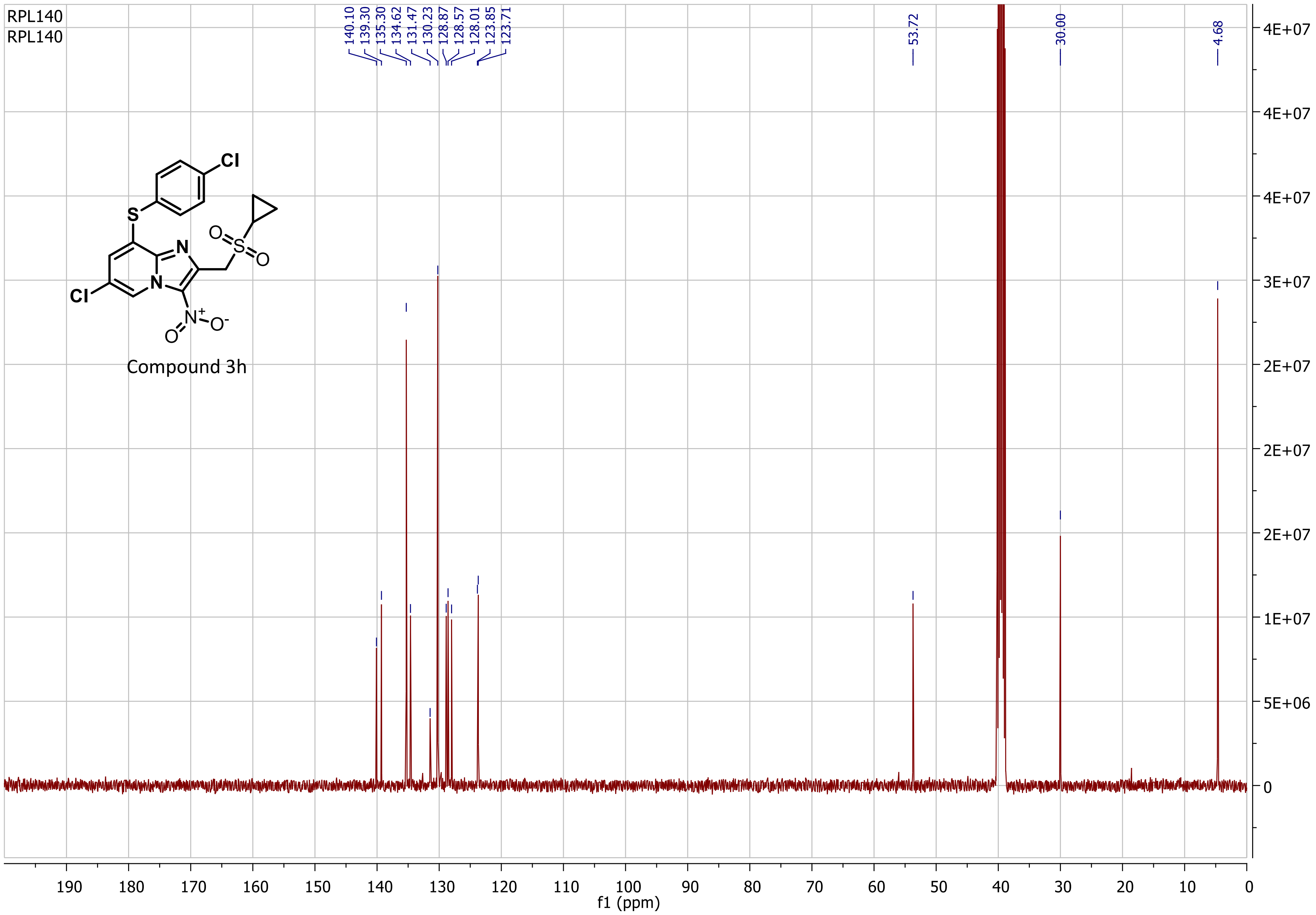
RPL140
RPL140
140.10
139.30
135.30
134.62
131.47
130.23
128.87
128.57
128.01
123.85
123.71
53.72
30.00
4.68
Compound 3h
f1 (ppm)

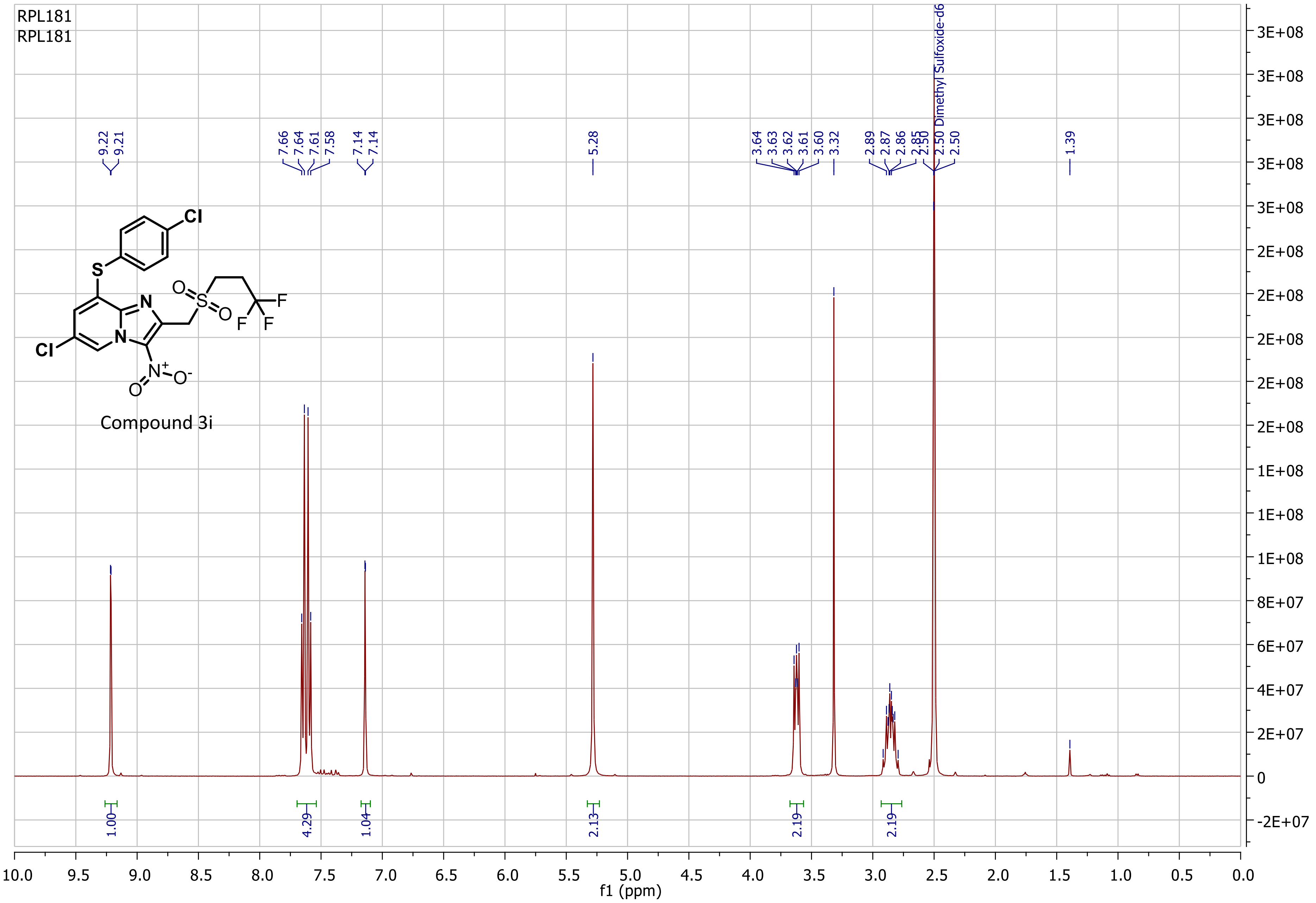

RPL181
RPL181
9.22
9.21
7.66
7.64
7.61
7.58
7.14
7.14
5.28
3.64
3.63
3.62
3.61
3.60
3.32
2.89
2.87
2.86
2.85
2.50
2.50 Dimethyl Sulfoxide-d6
2.50
1.39
Compound 3i
1.00
4.29
1.04
2.13
2.19
2.19
f1 (ppm)

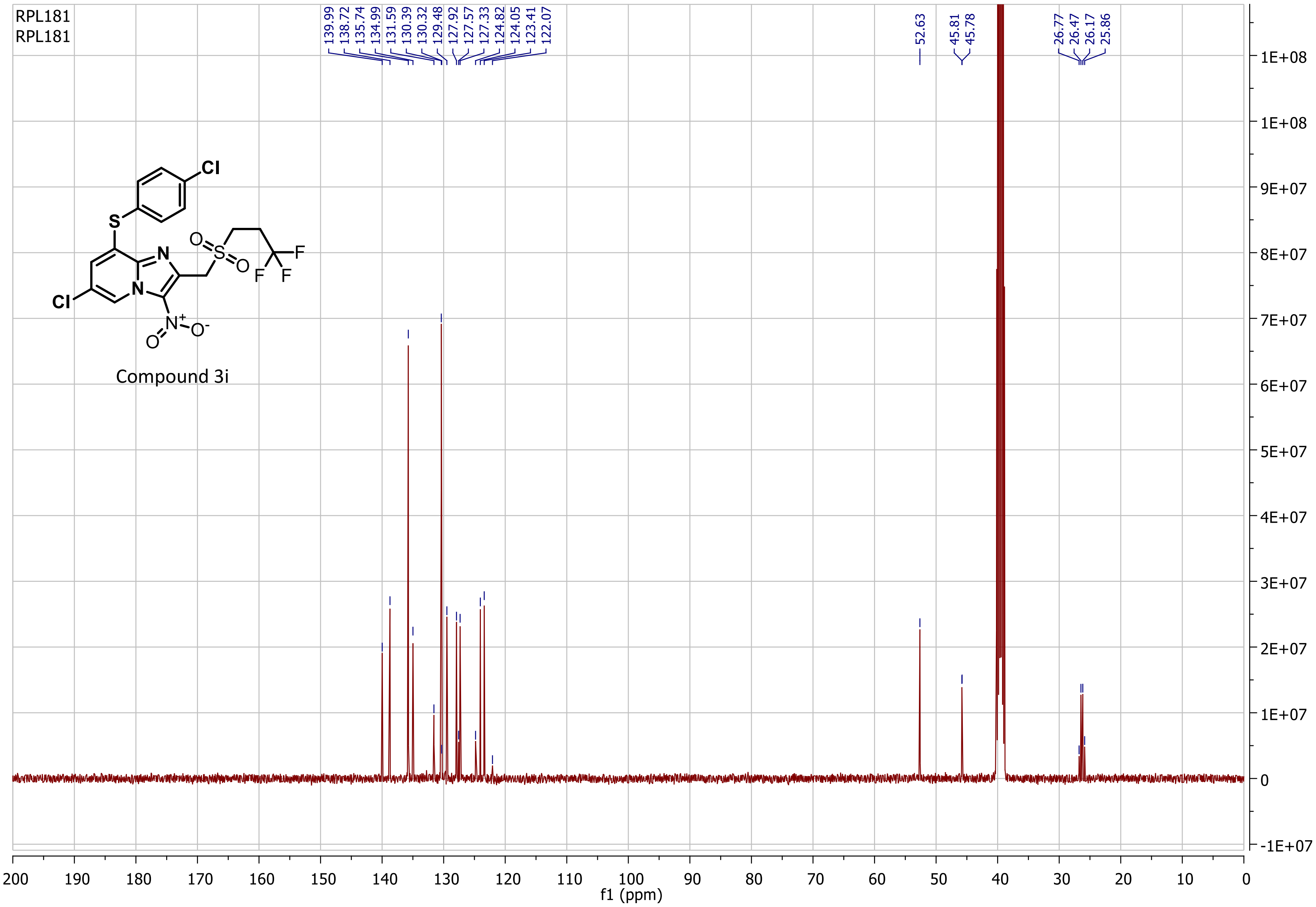

RPL181
RPL181
139.99
138.72
135.74
134.99
131.59
130.39
130.32
129.48
127.92
127.57
127.33
124.82
124.05
123.41
122.07
52.63
45.81
45.78
26.77
26.47
26.17
25.86
Cl
S
O
N
S
O
F
F
F
Cl
N
N+
O-
O
Compound 3i
1E+08
1E+08
9E+07
8E+07
7E+07
6E+07
5E+07
4E+07
3E+07
2E+07
1E+07
0
-1E+07
200
190
180
170
160
150
140
130
120
110
100
90
80
70
60
50
40
30
20
10
0
f1 (ppm)

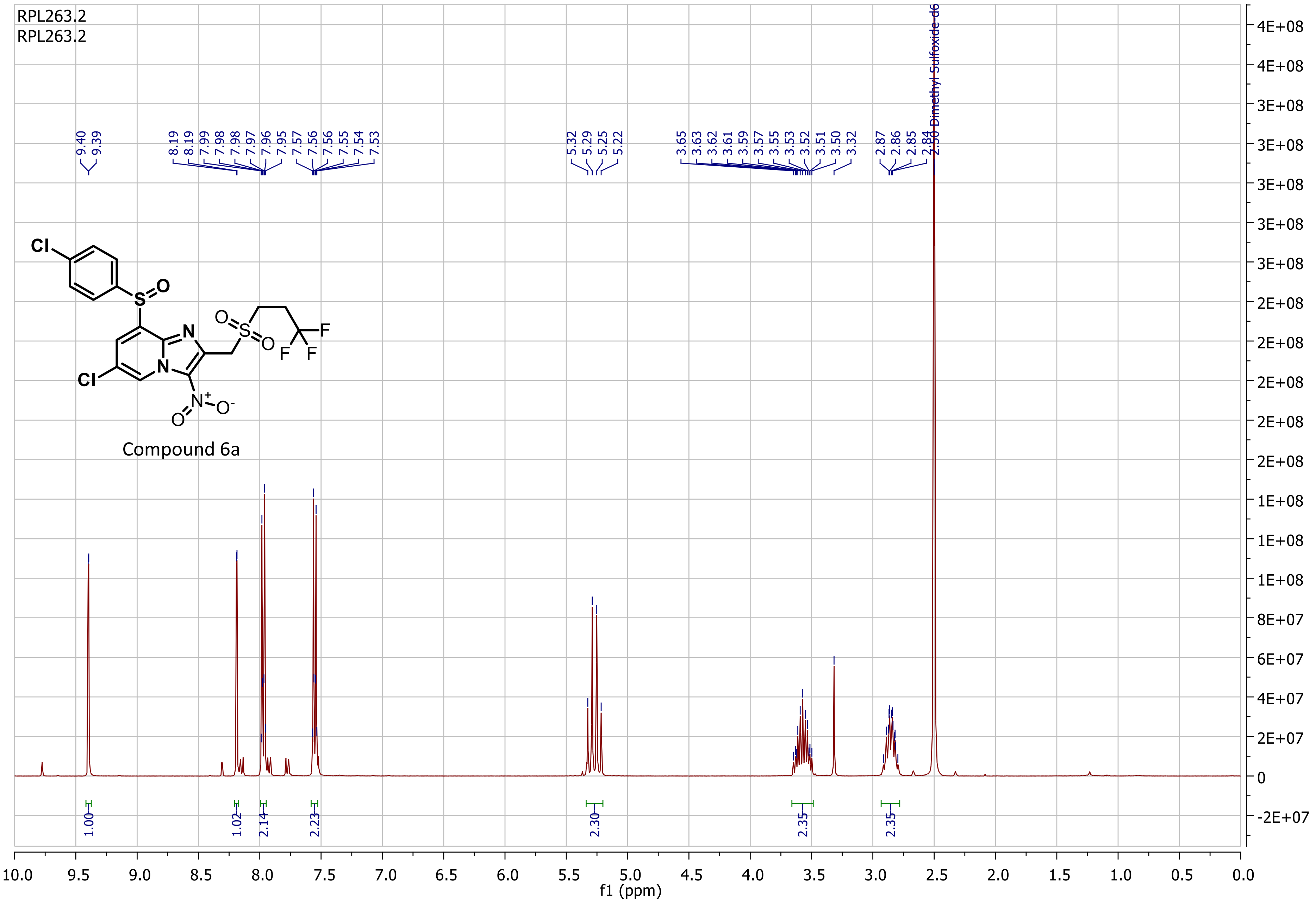

RPL263.2
RPL263.2
Compound 6a
f1 (ppm)
2.50 Dimethyl Sulfoxide-d6

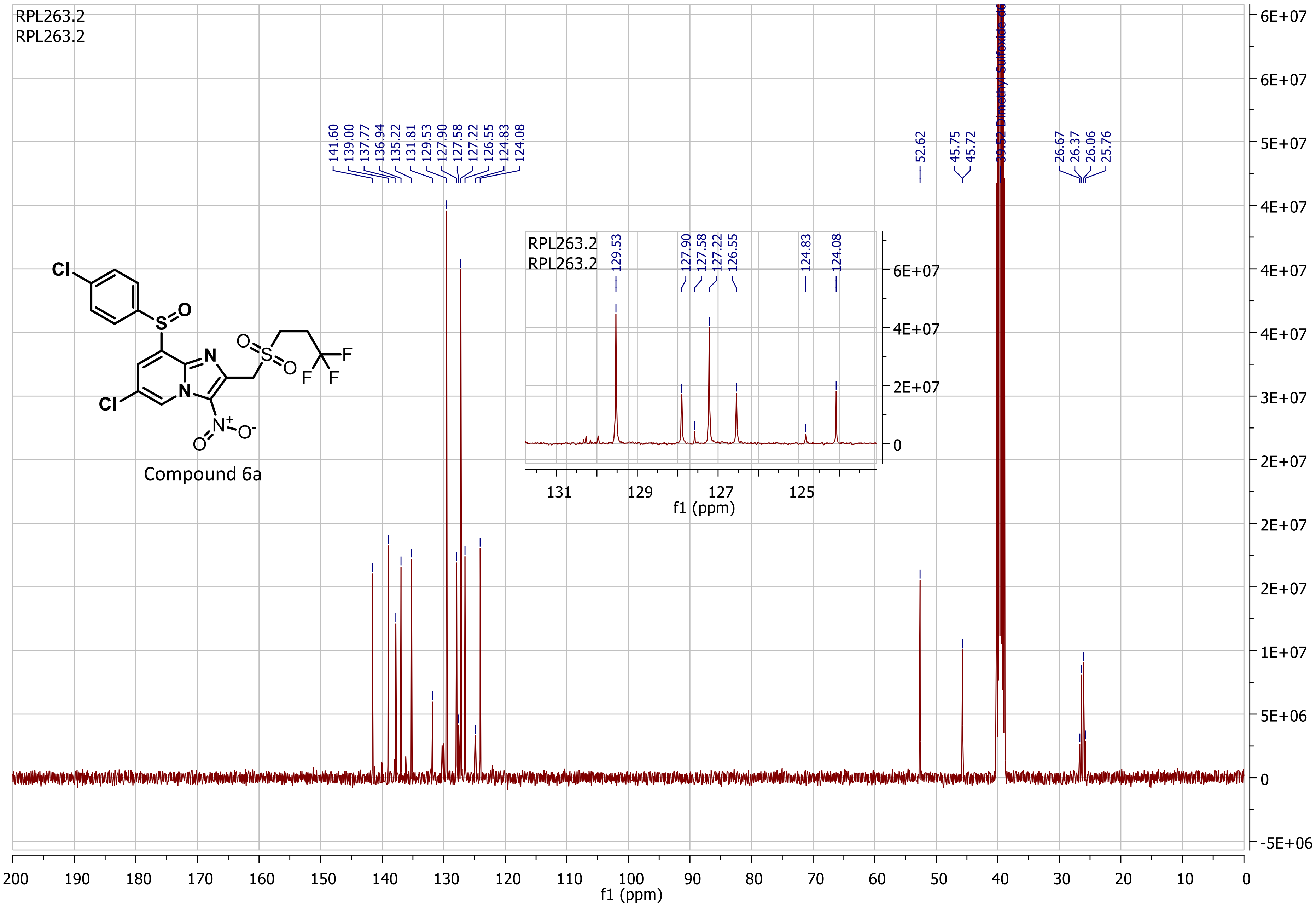
RPL263.2
RPL263.2
141.60
139.00
137.77
136.94
135.22
131.81
129.53
127.90
127.58
127.22
126.55
124.83
124.08
52.62
45.75
45.72
39.52 Dimethyl Sulfoxide-d6
26.67
26.37
26.06
25.76
Compound 6a
f1 (ppm)

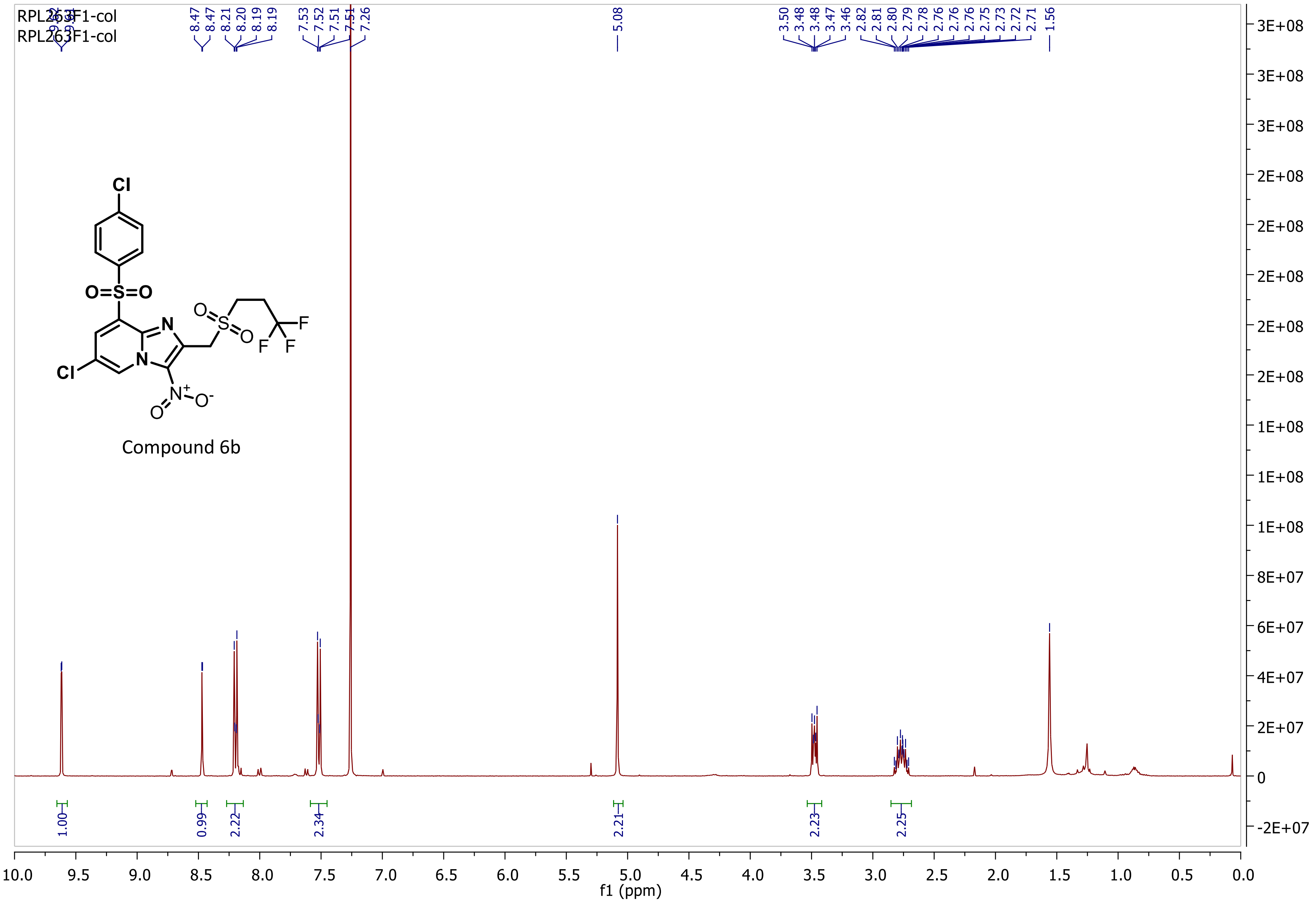
RPL263F1-col
RPL263F1-col
8.47
8.47
8.21
8.20
8.19
8.19
7.53
7.52
7.51
7.51
7.26
5.08
3.50
3.48
3.48
3.47
3.46
2.82
2.81
2.80
2.79
2.78
2.76
2.76
2.76
2.75
2.73
2.72
2.71
1.56
Compound 6b
1.00
0.99
2.22
2.34
2.21
2.23
2.25
f1 (ppm)

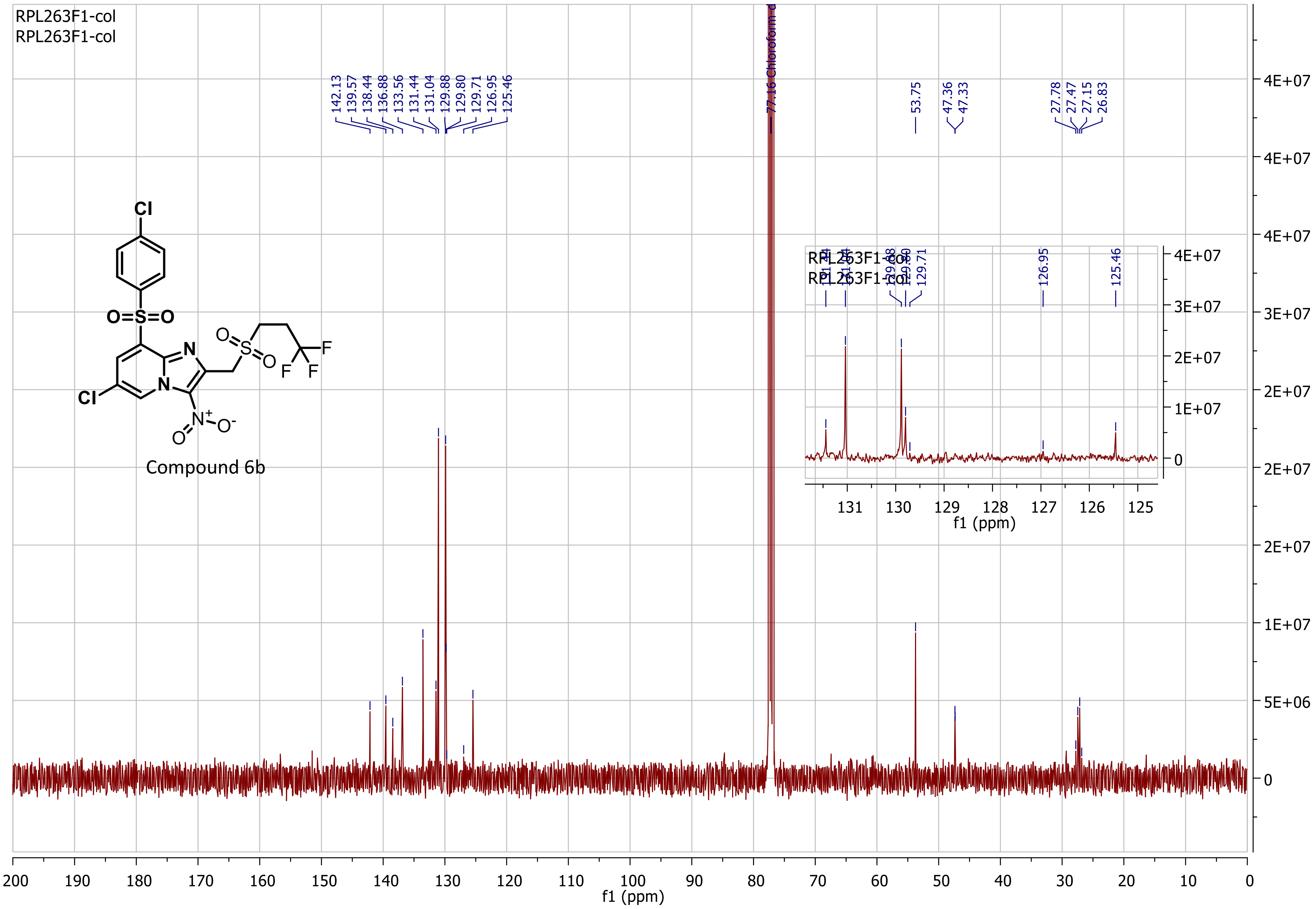
RPL263F1-col
RPL263F1-col
142.13
139.57
138.44
136.88
133.56
131.44
131.04
129.88
129.80
129.71
126.95
125.46
77.16 Chloroform-d
53.75
47.36
47.33
27.78
27.47
27.15
26.83
Compound 6b
129.71
126.95
125.46
f1 (ppm)
f1 (ppm)

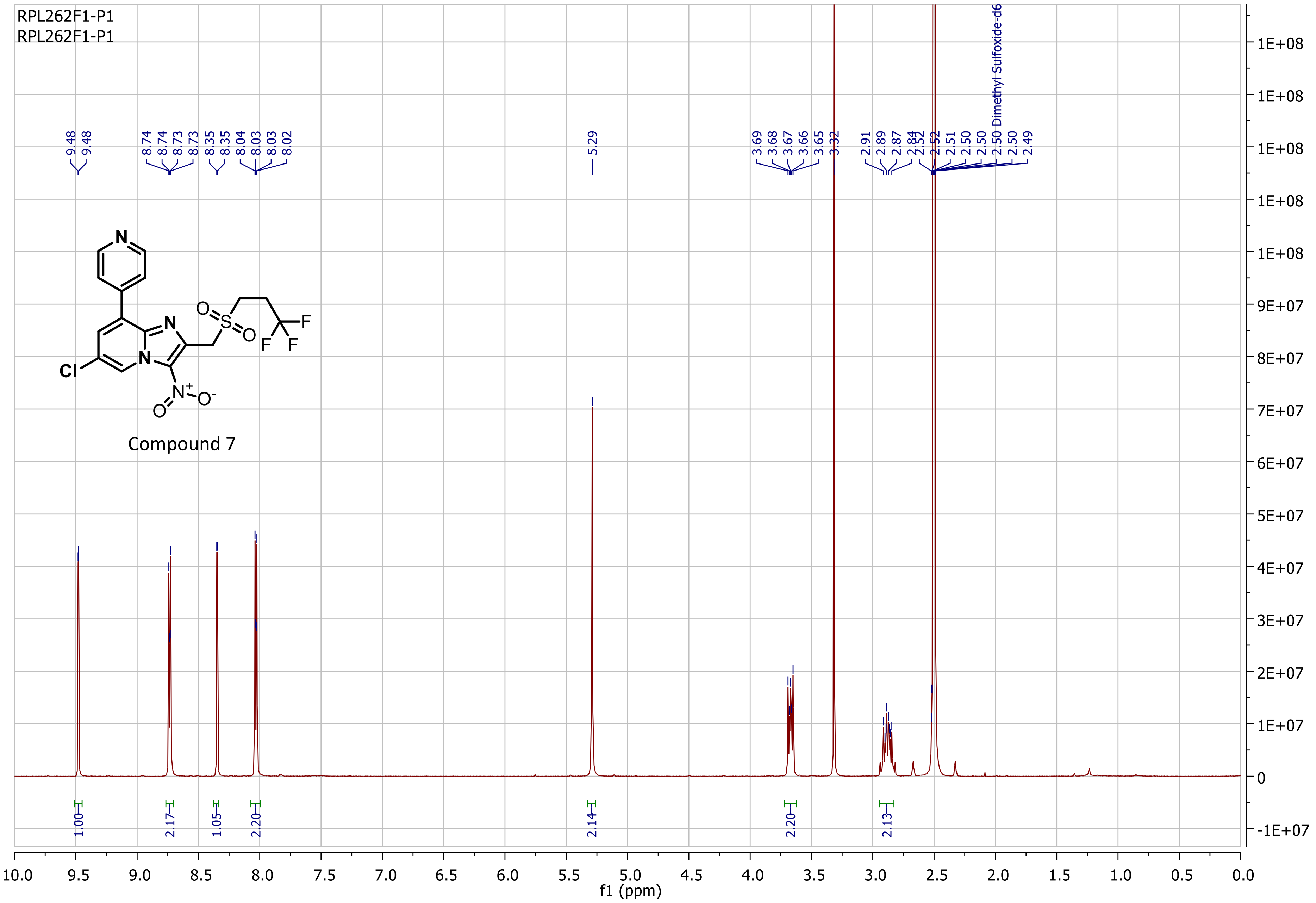
RPL262F1-P1
RPL262F1-P1
9.48
9.48
8.74
8.74
8.73
8.73
8.35
8.35
8.04
8.03
8.03
8.02
5.29
3.69
3.68
3.67
3.66
3.65
3.32
2.91
2.89
2.87
2.84
2.52
2.52
2.51
2.50
2.50
2.50 Dimethyl Sulfoxide-d6
2.50
2.49
N
O
S
O
F
F
F
N
N
Cl
N+
O
O-
Compound 7
1.00
2.17
1.05
2.20
2.14
2.20
2.13
f1 (ppm)

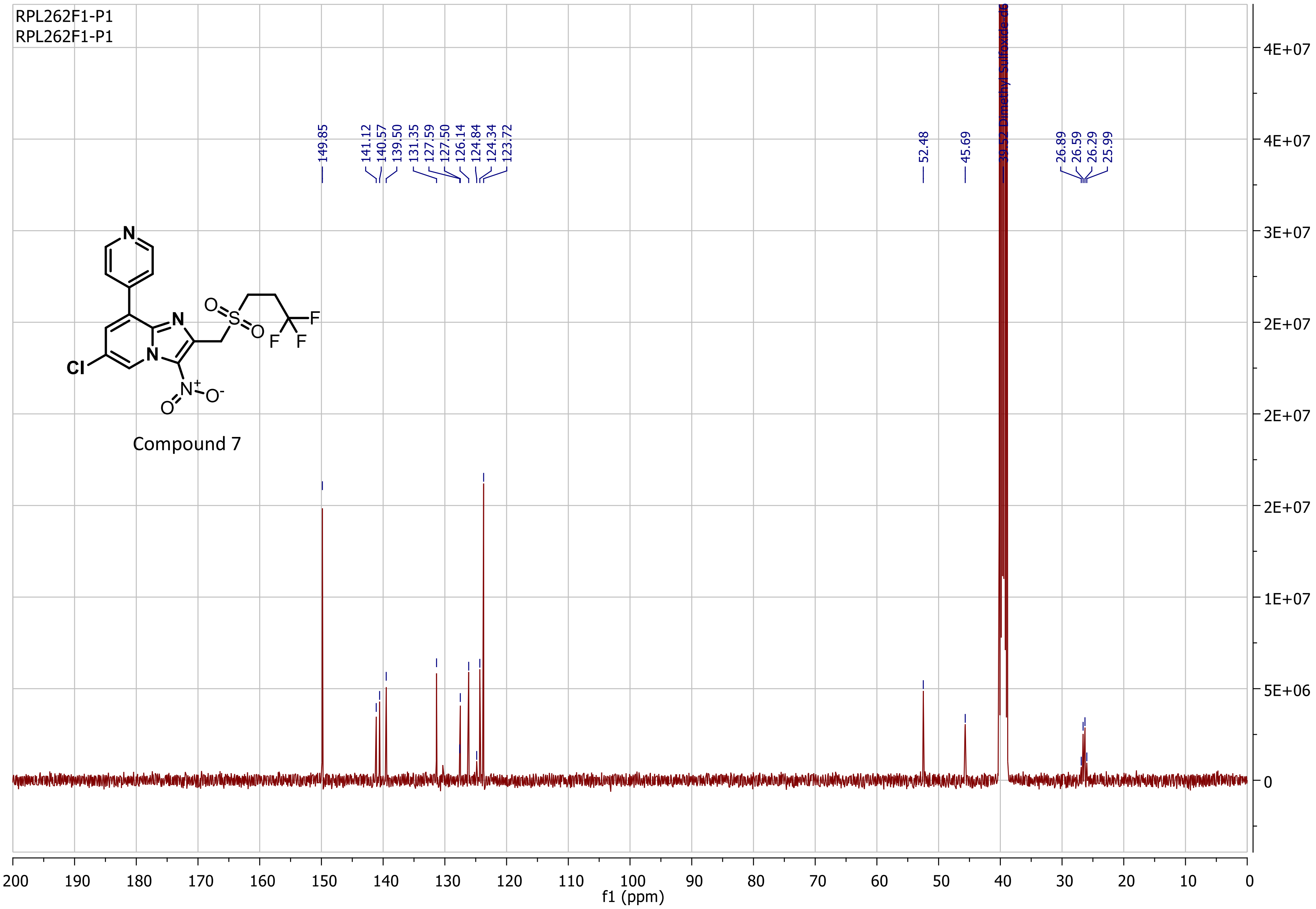
RPL262F1-P1
RPL262F1-P1
149.85
141.12
140.57
139.50
131.35
127.59
127.50
126.14
124.84
124.34
123.72
52.48
45.69
39.52 Dimethyl Sulfoxide-d6
26.89
26.59
26.29
25.99
4E+07
4E+07
3E+07
2E+07
2E+07
2E+07
1E+07
5E+06
0
200
190
180
170
160
150
140
130
120
110
100
90
80
70
60
50
40
30
20
10
0
f1 (ppm)
N
O
S
O
F
F
F
N
N
Cl
N+
O-
O

Compound 7
